# Toxic Cyanobacteria in Svalbard: Chemical Diversity of Microcystins Detected Using a Liquid Chromatography Mass Spectrometry Precursor Ion Screening Method

**DOI:** 10.3390/toxins10040147

**Published:** 2018-04-03

**Authors:** Julia Kleinteich, Jonathan Puddick, Susanna A. Wood, Falk Hildebrand, H. Dail Laughinghouse IV, David A. Pearce, Daniel R. Dietrich, Annick Wilmotte

**Affiliations:** 1 Center for Applied Geosciences, Eberhard Karls Universität Tübingen, Hölderlinstr. 12, 72074 Tübingen, Germany; 2 BCCM/ULC, University of Liege, In-Bios Centre for Protein Engineering, B6, 4000 Liege, Belgium; awilmotte@uliege.be; 3 Cawthron Institute, Halifax Street East, Nelson 7010, New Zealand; jonathan.puddick@cawthron.org.nz (J.P.), susie.wood@cawthron.org.nz (S.A.W.); 4 Structural and Computational Biology, European Molecular Biology Laboratory, Meyerhofstrasse 1, 69117 Heidelberg, Germany; falk.hildebrand@gmail.com; 5 Fort Lauderdale Research and Education Center, University of Florida, Davie, FL 33314, USA; hlaughinghouse@ufl.edu; 6 Department of Applied Sciences, Faculty of Health and Life Sciences, University of Northumbria at Newcastle, Newcastle NE1 8ST, UK; david.pearce@northumbria.ac.uk; 7 British Antarctic Survey, Cambridge CB3 0ET, UK; 8 Human and Environmental Toxicology, University of Konstanz, 78457 Konstanz, Germany; daniel.dietrich@uni-konstanz.de

**Keywords:** arctic, benthic mats, cyanotoxins, ELISA, 16S rRNA gene

## Abstract

Cyanobacteria synthesize a large variety of secondary metabolites including toxins. Microcystins (MCs) with hepato- and neurotoxic potential are well studied in bloom-forming planktonic species of temperate and tropical regions. Cyanobacterial biofilms thriving in the polar regions have recently emerged as a rich source for cyanobacterial secondary metabolites including previously undescribed congeners of microcystin. However, detection and detailed identification of these compounds is difficult due to unusual sample matrices and structural congeners produced. We here report a time-efficient liquid chromatography-mass spectrometry (LC-MS) precursor ion screening method that facilitates microcystin detection and identification. We applied this method to detect six different MC congeners in 8 out of 26 microbial mat samples of the Svalbard Archipelago in the Arctic. The congeners, of which [Asp^3^, ADMAdda^5^, Dhb^7^] MC-LR was most abundant, were similar to those reported in other polar habitats. Microcystins were also determined using an Adda-specific enzyme-linked immunosorbent assay (Adda-ELISA). *Nostoc* sp. was identified as a putative toxin producer using molecular methods that targeted 16S rRNA genes and genes involved in microcystin production. The *mcy* genes detected showed highest similarities to other Arctic or Antarctic sequences. The LC-MS precursor ion screening method could be useful for microcystin detection in unusual matrices such as benthic biofilms or lichen.

## 1. Introduction

Cyanobacteria are phototrophic prokaryotes that occur in a diverse range of terrestrial and aquatic ecosystems worldwide. They are most infamously known for their mass occurrence (blooms) in tropical and temperate freshwaters [1,2]. These blooms are becoming progressively more problematic as they are reinforced by increasing nutrient loads and elevated water temperature mediated by climate change [3,4]. Many of the bloom-forming cyanobacterial species produce toxic secondary metabolites that pose a threat to human and animal health [5,6]. The compounds include heptapeptides with hepato- and neurotoxic potential, as well as neurotoxic and cytotoxic alkaloids [5,6,7,8]. Planktonic cyanobacterial blooms and the associated toxins have a direct impact on drinking water quality, the usability of water for recreational activities and receive significant attention from the scientific community, media and the general public [6,9]. It is important to understand the potential chemical diversity of cyanobacterial toxins and identify cyanobacterial species producing them to assist in management and risk assessment of cyanobacterial blooms. This knowledge may also help in understanding the evolution and the ecological function of the secondary metabolites.

In contrast to planktonic species, far less scientific and public attention has been devoted to non-planktonic habitats. Cyanobacteria growing in benthic mats, however, may also provide a source for novel secondary metabolites. Quite recently, it has been shown that cyanobacterial species in benthic mats, lichen-associations or epilithic biofilms produce toxins [8,9,10] as well as novel toxin congeners [11,12] previously undescribed from typical planktonic blooms.

**Figure 1:**
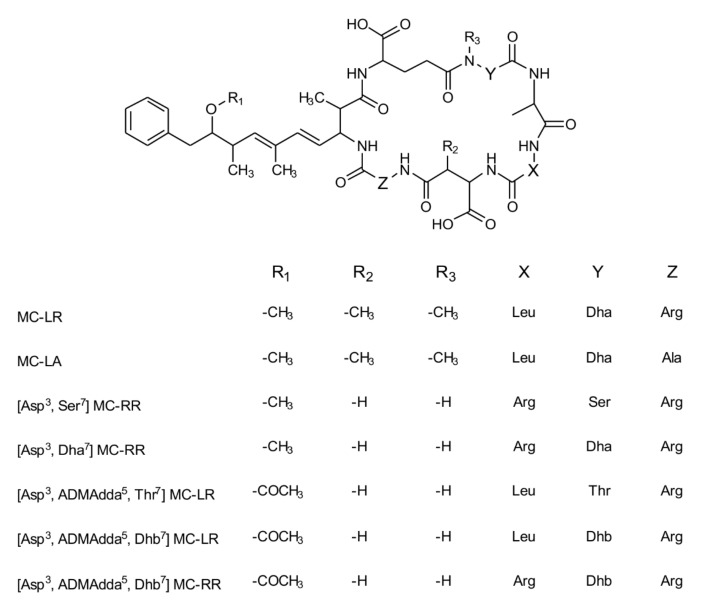
Structure of microcystin-LR and the 6 microcystin (MC) congeners identified in this study (ADMAdda = *O*-acetyl-*O*-demethyl 3-amino-9-methoxy-2,6,8-trimethyl-10-phenyldeca-4,6-dienoic acid, Ala = alanine, Arg = arginine, Asp = aspartic acid, Dha = dehydroalanine, Dhb = dehydrobutyrine, Leu = leucine, Ser = serine and Thr = threonine).

Freshwater habitats of the polar regions are inhabited by a large taxonomic diversity of benthic cyanobacterial species [13,14]. Recent studies show that 20–96% of screened polar samples contain cyanobacterial toxins [15,16,17,18,19,20]. Benthic cyanobacterial mats in polar meltwater ponds, cryoconite holes, wet soil and marshy moss cushions are therefore suitable candidates to discover potentially toxic cyanobacteria and new secondary metabolites [20]. The neurotoxic saxitoxin was detected in a benthic cyanobacterial community from the Arctic [18] and the cytotoxic cylindrospermopsin was found in a similar habitat in the Antarctic. However, no known toxin-producing organisms were observed in polar samples and these have yet to be identified [19]. Microcystins (MCs), the most commonly identified and widely distributed cyanotoxins, have also been detected in cyanobacterial mats from the Arctic and the Antarctic. Microcystins are cyclic heptapeptides (Figure 1) composed of seven d- and l- amino acids, including uncommon amino acids such as 3*S*-amino-9*S*-methoxy-2*S*,6,8*S*-trimethyl-10-phenyldeca-4*E*,6*E*-dienoic acid (Adda) or *N*-methyl dehydroalanine (Mdha). The number of known MC variants currently exceeds 250 [21]. This variety is mainly based on two variable amino acids and modifications of the amino acids, such as methylation [12]. Microcystins found in the polar regions include a range of uncommon or previously unknown variants [11,12,17,22]; e.g., congeners that contained the rare substitution of the position one amino acid, the usual d-alanine, to glycine [12,17]. Microcystins act as protein phosphatase inhibitors in eukaryotic cells, inducing a breakdown of the cellular cytoskeleton and eventually leading to cell death, but they require active transport/uptake into the cell via organic anion transporting polypeptides (OATPs) [23]. The structure of the MC congeners affects their protein phosphatase inhibition and cellular uptake characteristics, and thus their final toxicity [8]. Accurate identification of MC congeners is therefore vital for risk assessment and freshwater management.

However, the increasing number of structural toxin congeners complicates the identification of MCs. Additionally, unusual sample types, such as polar benthic microbial mats, have a complex matrix containing pigments, polysaccharides and secondary metabolites [18,24]. These and other compounds may interfere with certain detection methods, for example by cross-reactivity of antibodies in an ELISA [25,26]. Analytical tools such as high-performance liquid chromatography (HPLC) and liquid chromatography-tandem mass spectrometry (LC-MS/MS) are often used for MC detection in complex matrices. Identification of unusual MCs using standard HPLC and LC-MS/MS methods, though, requires comparison with costly reference standards or time-consuming identification and structural characterization by experienced personnel.

The aims of this study were to (1) develop an LC-MS precursor ion scanning method that would simplify the identification and characterization procedure by reducing the number of candidate compounds that need to be characterized, and (2) to use this methodology to screen environmental microbial mat samples with a complex sample matrix collected from the Arctic.

## 2. Results

**Figure 2:**
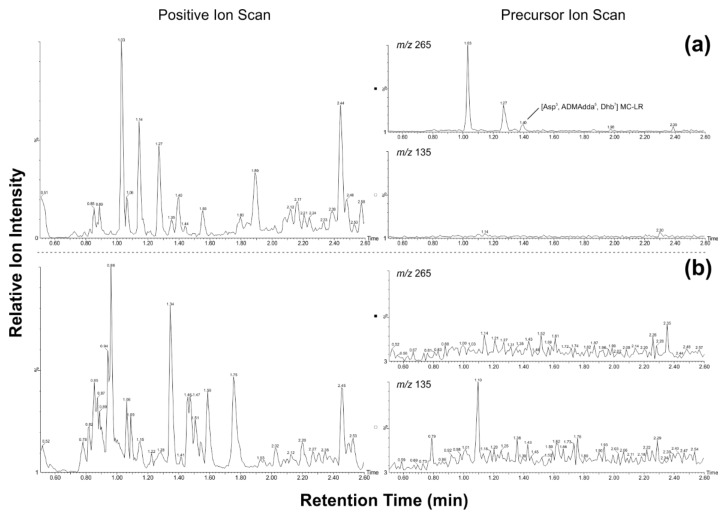
Base-peak chromatograms of positive ion scans (left; *m*/*z* 450-1150) and precursor ion scans (right; *m/z* 135 for Adda-containing compounds or *m/z* 265 for ADMAdda-containing compounds) for (**a**) SV-81 (Category 1) and (**b**) SV-02 (Category 3). See Table 1 for definition of categories.

For this study, 26 cyanobacteria dominated microbial mat samples from Svalbard were available (Supplementary Table S1 and Figures S1 and S2). Twenty of the samples were analyzed using an Adda-specific ELISA (Table 1). Of those 20 samples, 18 showed a signal above the detection limit in the ELISA, ranging between 2 and 54 µg of microcystin per liter extract. In three of these samples (SV-54, -74, and -75), the measured MC concentration exceeded the range of the standard curve despite several dilution steps and the MC concentration was therefore estimated to be above 50 µg/L. Twelve of the samples that were positive in the Adda-ELISA and six additional samples (SV-A, -B, -C, -D, -E and -81, not analyzed by ELISA) were analyzed using the MC congener precursor ion scanning method developed in this study (Table 1 and Figure 2). 

When the chromatograms from the precursor ion screen were compared to those acquired by collecting positive ion scan data (*m/z* 450-1,150; Figure 2), the data collected using the LC-MS precursor ion scanning method contained fewer candidate ions as expected. For the 18 samples analyzed during this study, there were 55% fewer peaks to further investigate when using the precursor ion scan searching for Adda product ions (Supplementary Table S2). 

For the purpose of identifying candidate MCs, compounds that eluted between 1–1.25 min with *m/z* 500–575 were assumed to be doubly-protonated ions of MCs containing two arginine residues. This premise was further strengthened by the presence of the corresponding singly-protonated ion between *m/z* 1000–1150. Compounds that eluted between 1.25–1.55 min with *m/z* 850–1150 were assumed to be MCs containing one arginine residue in position two (Figure 1). Compounds in the same mass region that eluted between 1.55–1.9 min were assumed to be MCs that contained one arginine residue in position four [27]. Finally, compounds that eluted between 1.9–2.35 min with an *m/z* 850–1150 were assumed to be hydrophobic MC congeners containing no arginine residues. These retention times were determined using available microcystin reference standards such as MCs -RR, -YR, -LR and -LF, and using an extract *Microcystis* CAWBG11 that produces a wide array of microcystins and has been well characterized in our laboratory [27]. From this analysis, the LC-MS precursor ion screens were classified in three categories: (1) MCs likely to be present in the sample; (2) MCs possibly present in the sample, and; (3) MCs absent from the sample. The classification between Categories 1 and 2 took into account whether MCs with the same precursor ion mass had been reported in the past [28]. Seven of the 18 samples analyzed using the precursor ion screen were classified in Category 1, two in Category 2 and the remaining nine in Category 3 (Table 1).

When the Category 1 and 2 samples were investigated further by MS/MS, all seven category 1 samples were positive for MC congeners. Six known MCs and an unidentified MC congener were detected (Figure 1, Table 1). The unidentified congener, detected in sample SV-83, had a mass of 1052 Da and contained dehydrobutyrine (Dhb), but its structure could not be elucidated at this point in time due to insufficient structural information from the product ions acquired. The most commonly observed MC congener in the samples was [Asp^3^, ADMAdda^5^, Dhb^7^] MC-LR (Figure 1), identified in SV-D, SV-49, SV-75, SV-80, SV-81 and SV-83.

The genes for non-ribosomal peptide synthesis (NRPS) and polyketide synthetases (PKS), involved in general secondary metabolite production were detected in 13 samples, irrespective of their category in the pre-cursor scan (Table 1). The *mcyB* and *mcy*E genes were shown to be present in four samples (SV-D, SV-75, SV-80 and SV-81), whereas only *mcyE* was detected in sample SV-E (Table 1). All samples containing *mcyE* or *mcyB* genes, except sample SV-E, were Category 1 in the LC-MS precursor ion scanning method and contained MC congeners as detected using detailed LC-MS/MS analysis. The partial sequences of the *mcyE* genes were most similar to those of the genus *Nostoc* sp. 152, with a pairwise similarity between 93% and 99% as well as to an uncultured *Nostoc* clone MVMG1 from Antarctica (98–99%) in SV-D, E, 75 and 80 and to an uncultured cyanobacterium isolate *nda*F gene from the Gulf of Finland (93%, Supplementary Table S3) in SV-81. The putative *mcyB* genes were related to non-ribosomal peptide synthetase gene cluster sequences in *Microcoleus* PCC-8701 with a maximum pairwise similarity of 73% as well as *Cylindrospermum* sp. NIES-4074 and *Scytonema* NIES-4073 whole genomes. Similarities of up to 76% were recorded to *Nostoc* strains, but with a lower sequence coverage. These similarities appear quite high as polar toxin gene sequence similarities to the sequences in GenBank from other habitats are generally low [18,19]. Moreover, genetic similarities for protein-coding genes are usually lower than for the highly conserved ribosomal subunit sequences. In order to focus on the functionality of the protein, we translated the DNA sequence into the corresponding amino acid sequence. Using the blastp search tool of GenBank, the *mcyE* amino acid sequences appeared most similar (>97%) to various Arctic and Antarctic *Nostoc* strains (Supplementary Table S3). The putative *mcyB* amino acid sequences were most similar (>83%) to a non-ribosomal peptide synthetase of the Antarctic cyanobacterium *Phormidesmis priestleyi* ULC007 (#WP_073072318.1).

In addition to MCs, two samples (SV-13 and SV-83) were positive in the cylindrospermopsin ELISA (data not shown). However, this could not be confirmed by HPLC or molecular analysis. A saxitoxin ELISA was negative for all samples assessed (data not shown), but a fragment of the *sxtA* gene was amplified in sample SV-75. The sequence matched with 100% similarity to a *sxt* sequence detected in a *Nostoc* dominated cyanobacterial mat from Baffin Island [18] and to *Scytonema* sp. UCFS15 from New Zealand with 98% similarity (Supplementary Table S3).

**Figure 3:**
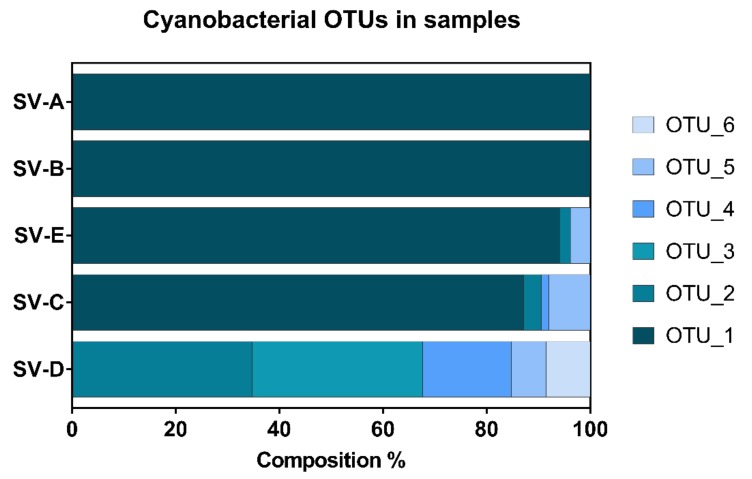
Phylogenetic composition of Svalbard samples based on 16S ribosomal RNA gene amplification using 454 sequencing and filtered for solely cyanobacterial reads. See text and Supplementary Figure S4 for identification of operational taxonomic units (OTUs).

To elucidate the cyanobacterial diversity of the samples and to identify potential toxin producers, five samples were analyzed by 454 next generation sequencing. The genomic library obtained from the partial 16S rRNA gene revealed a high homogeneity between the samples (Figure 3). The dominant OTU (OTU1) in three samples belonged to the family Oscillatoriaceae, with the highest match on GenBank being an uncultured bacterium clone from the Antarctic Peninsula. Similarities above 98% to cultured strains were observed for *Phormidium* sp. JR20 later identified as *Microcoleus favosus* JR20 [29] and *Phormidium* sp*.* Ant-Orange, both isolated from Antarctica. Representative sequences and putative identities of the 6 most abundant OTUs can be found in Supplemental Table S4. One sample, SV-D, was distinctly different from the other samples and not dominated by OTU1. SV-D had a higher taxonomic richness and was composed by five OTUs assigned to the *Oscillatoriales* family (OTU2 and 6), *Tolypothrix* (OTU3) and *Nostoc* (OTU4 and 5). OTU4 had highest similarities to a sequence from Baffin Island in the Canadian Arctic [18]. Upon macroscopic and microscopic examination, *Nostoc* was clearly dominant in sample SV-D and SV-E; however, it was only a minor component in the 454 sequencing analysis. Although SV-C, SV-D, and SV-E contained OTUs that could be assigned to the *Nostoc* genus, the *Nostoc* specific OTUs did not exceed 20% of the sequences in any of the samples.

## 3. Discussion

In the present study, we show that the cyanobacterial toxin MC is widely distributed in benthic cyanobacterial mats in the Svalbard archipelago. The identified MC congeners were chemically diverse and structurally similar to MC congeners detected previously in polar habitats [11,12,18,19]. The presence of MCs in our samples as well as the detection of toxins and other compounds with bioactive potential in other studies [11,12,18,19,30,31,32] indicate that cyanobacteria in polar regions could be a rich source of secondary metabolites.

Cyanobacterial toxins are commonly known from warm habitats but have been reported repeatedly in the polar regions over the last two decades [11,15,16,17,18,19,20,33]. Previous records of cyanotoxins in Svalbard reported the presence of MC variants in samples of cyanolichens [11] and MC-LR in biocrusts [33], without an exact determination of the MC variant in the latter study. In the present study, 90% of the samples were positive using the Adda-MC ELISA. The presence of specific MC variants was confirmed by LC-MS/MS analysis in 20% of the samples. A clear signal in the LC-MS precursor ion screen was confirmed by further MS/MS characterization in seven samples. The detection of the *mcyE* and *mcyB* genes confirmed the presence of a toxin producer in five samples.

More samples tested positive for MC in the ELISA than in the LC-MS analysis. This difference may be explained by the lower detection limit and the cumulative signal of all Adda-containing molecules in the ELISA. In contrast, LC-MS targets single compounds at a higher detection limit. However, the LC-MS precursor ion screen provided more definitive information than the Adda-ELISA. The ELISA seemed to have more problems with the complex sample matrix of Arctic environmental samples containing pigments, polysaccharides and other secondary metabolites [24] that may interfere with antibodies and other reagents [25,26].

The LC-MS precursor ion scanning method allowed candidate MCs to be identified more easily than traditional LC-MS identification methods. In our study, the precursor ion scan provided 57% fewer peaks to assess when compared to conducting a preliminary assessment using a conventional positive ion scan of the appropriate mass range. Furthermore, because the precursor ion scan assesses for product ions associated with MCs, there is more confidence that further investigation will result in the detection of MCs. Conventional dereplication strategies were employed to reduce the number of candidate ions by a further 68% (i.e., comparing the retention time and *m/z* with that which would be expected for MCs). Traditional MS/MS characterization techniques were still required to confirm the identity of candidate ions, but the workload was reduced significantly by decreasing the number of candidate compounds through the MC precursor ion screen.

Whilst the MC congeners detected most frequently during this study are rarely detected in planktonic cyanobacterial blooms, they are commonly observed in Arctic and Antarctic habitats [11,12,17,18,19]. This includes the desmethyl Asp^3^ modification, the substitution of *N*-methyl dehydroalanine (Mdha) to dehydrobutyrine (Dhb) at position seven (Figure 1) as well as the acetyl desmethyl modification of the position five Adda group (ADMAdda), which are commonly observed in MCs produced by *Nostoc* species [27]. However, the SV-74 sample contained the more conventional MC-LA, which is observed in planktonic cyanobacterial blooms relatively frequently. In SV-80, Ser^7^ and Thr^7^ congeners were present. These amino acids are the precursor compounds for Dha^7^ and Dhb^7^, commonly observed in position seven of MCs. Their presence indicates that either the dehydrogenase enzyme in the non-ribosomal peptide synthetase (NRPS) module was not functioning effectively [34] in the dominant cyanobacterial strain or that multiple MC-producing strains were present and the dehydrogenase gene was not functional in a subset of the population. Isolation of cultured strains would be required to further understand the MC diversity observed in these samples, highlighting some of the limitations of working on environmental material.

The chemical diversity of MCs and geographic connection was also reflected in the MC synthetase (*mcy*) genes and translated amino acid sequences amplified here. These sequences were most similar to *mcyE* sequences of an uncultured *Nostoc* detected in Antarctic habitats [17] and to an *ndaF* gene of an uncultured cyanobacterium in the Gulf of Finland [35]. The closest cultured match, *Nostoc* sp.152, was originally isolated in Finland [36]. For the putative *mcyB* gene, no close similarities (>90%) to gene sequences from environmental sources could be detected, indicating that the amplified product may be an NRPS but not located on a *mcy* operon. Nevertheless, more than 83% similarity to a non-ribosomal peptide synthetase amino acid sequence of the Antarctic cyanobacterium *Phormidesmis priestleyi* ULC007 suggests a geographic connection to polar organisms. The here detected nucleotide and translated amino acid sequences differ from those of species usually detected in planktonic blooms of warmer climate such as *Microcystis aeruginosa* (e.g., NIES-843, accession #NC_010296) or *Planktothrix rubescens* (e.g., NIVA-CYA 407 accession #NZ_AVFW00000000). Unfortunately, no known Arctic or Antarctic MC-producing strain has been isolated or cultivated to date and few sequences of the *mcy* genes are available from polar habitats, so that a comprehensive geographic distribution analysis of toxin genes is not possible. The full genome sequencing of an Arctic/Antarctic MC producer, or more *mcy* sequences from polar regions, could help to understand the detected MC diversity.

During this study, the cyanobacterial genus *Nostoc* was identified as a likely candidate for producing MCs on Svalbard. This genus has previously been suspected to be a producer of MCs in the polar regions [17,18,20] and the results of this study support this suggestion. *Nostoc* was predominant, identified macroscopically and microscopically, in samples containing the highest concentrations of MC (Samples SV-D, -E, -49, -56, -74 and -75). Moreover, the *mcyE* genes detected were most similar to *mcyE* genes of the *Nostoc* genus as described above. In addition, Kaasalainen et al. [11] detected MCs in lichen-associated *Nostoc* on Svalbard. The *Nostoc* genus is widely distributed on the Svalbard archipelago and may make a significant contribution to local nitrogen cycling [37].

Even though *Nostoc* clearly predominated some of the samples, the genus was underrepresented in the pyrosequencing results. This underrepresentation of *Nostoc* is likely an artifact of insufficient DNA extraction due to the extensive exopolysaccharidic sheaths of the Nostocalean family possibly in combination with primer biases [38]. This highlights the limits of next generation sequencing techniques, which can only be regarded as semi-quantitative [39,40]. The dominant OTUs detected in the five samples analyzed were similar to other Arctic or Antarctic cyanobacterial sequences (Supplemental Table S4). A circumpolar cold-climate biogeography of cyanobacterial species has been suggested before [41,42] and needs to be studied in detail in the future. Unfortunately, no cyanobacterial sequences from the Russian Arctic are available, but they would be a valuable addition for polar cyanobacterial biogeography studies.

Interestingly, the three samples from a polar geothermal spring with temperatures of around 20 °C contained relatively more congeners than samples from other habitats on the Svalbard archipelago. This would suggest that Arctic MC producers increase production in warmer temperatures or that warmer temperatures support different strains of cyanobacteria. It has been shown previously that polar cyanobacterial communities, when growing at warmer temperatures, changed species composition and increased MC production [43]. Another hypothesis is that MC producers are adapted to warmer habitats and thrive in the hot springs, though they are normally able to survive in the cold polar climate. Geothermal sites in the polar regions could thus serve as a refuge for organisms generally adapted to a warmer climate as was inferred by Fraser et al. [44] for Antarctica. As a possible consequence, a temperature rise due to climate change, like the one already ongoing in some parts of the Arctic and the Antarctic [45], could lead to an increased abundance of toxic cyanobacteria in Svalbard.

In summary, benthic cyanobacteria from Svalbard proved to be a rich source for structurally interesting MC congeners. The precursor ion screening tool facilitated the identification of six different MC congeners by specifically assessing product ions diagnostic of MCs. The MC congeners produced were structurally dissimilar to those usually observed in planktonic blooms in warmer regions, but were similar to those produced by the genus *Nostoc*. It is possible that the ‘unusual’ MC variants detected in extreme environments are not unusual, but have been overlooked in temperate and other bioregions to date. It has been suggested that “Given the ecological plasticity of cyanobacteria […] potentially toxic cyanobacteria are much more widely distributed than currently thought.” [20]. Consequently, cyanobacteria worldwide and in all habitat types (e.g., terrestrial and aquatic, benthic and planktonic) may contain a yet unknown diversity of MC variants as well as other secondary metabolites. Benthic cyanobacteria in polar, temperate and tropical environments remain an interesting source for the identification of bioactive compounds including MCs. The precursor ion screening methodology described here will assist in the discovery of these MC congeners as it simplifies and speeds up the identification of non-conventional congeners. 

## 4. Materials and Methods 

Svalbard is an archipelago in the North Atlantic Ocean at 77°50′ N and 19°50′ E. Samples were collected during two field seasons (Supplementary Table S1). Five cyanobacterial samples were collected at various locations in the vicinity of Longyearbyen, Björndalen and Colesbukta in June 2012 and 21 samples in a diversity of biotopes across the entire archipelago in July 2013 (Supplementary Figure S1). Cyanobacterial biofilms, mats and crusts were collected from streams, wet soil, moss cushions and in ‘hot springs’ with water temperature up to 25 °C (Supplementary Figure S2, Supplementary Table S1). Biofilm material was directly sampled using a sterile spatula and stored in sterile tubes or bags and were stored at −20 °C within 8 h from collection until further analysis. Biofilm material was examined microscopically and macroscopically in the laboratory.

For MC extraction, the frozen material was thawed, homogenized with a sterile spatula and lyophilized. In a methanolic extraction step, organic compounds were extracted as follows: one mL of 80% aqueous methanol (*v*/*v*) with 0.1% formic acid (*v*/*v*) was added to 0.02–2.4 mg of lyophilized material (Table 1), incubated for 2 h at room temperature, vortexed vigorously, and ultra-sonicated for 15 min. The organic material was pelleted by centrifugation (11,400× *g*, 10 min) and the extraction repeated twice on the pellet. The resulting supernatants were combined and dried at 37 °C under continuous nitrogen flow or in a speed-vac (Savant SPD111V, Thermo Fisher Scientific, Waltham, MA, USA). The dried extracts were re-solubilized (200 µL, 80% *v*/*v* methanol with 0.1% formic acid; *v*/*v*), centrifuged (13,000× *g*, 15 min) to remove residual particles and stored at −20 °C until LC-MS analysis. The samples collected during 2013 were further purified using C_18_ cartridges (Sep-Pak, Waters, Dublin, Ireland) as described previously [19] to decrease matrix effects in the subsequent ELISA.

Twenty samples collected during 2013 were screened using ELISA for MCs (MC-ADDA ELISA), cylindrospermopsins (CYNs) and saxitoxins (STXs) according to the recommended protocol (ABRAXIS, Warminster, PA, USA). The assays have an LOD of 0.15 ng MC/mL, 0.05 ng CYN/mL and 0.02 ng STX/mL, respectively.

Based on the preliminary results, 12 samples that generated a strong MC signal in the ELISA and five additional extracts from the 2012 field trip plus one from hot spring were selected for analysis using LC-MS applying the precursor ion screening method. Clarified extracts were analyzed using an Acquity I-Class ultra-performance liquid chromatography system (UPLC; Waters Ltd., Borehamwood, UK) coupled to a Xevo-TQS triple quadrupole mass spectrometer (Waters Ltd.). Compound separation was achieved using an Acquity BEH-C_18_ UPLC column (Waters Ltd.; 1.7-µm; 50 × 2.1 mm) at 40 °C. Sample components were eluted at 0.4 mL/min with a gradient from 10% acetonitrile (Solvent A; *v*/*v*) to 90% acetonitrile (Solvent B; *v*/*v*), each containing 100 mM formic acid and 4 mM ammonia. The sample extracts (5 µL) were injected at 5% Solvent B (*v*/*v*) and held for 12 s before a linear gradient up to 35% Solvent B (*v*/*v*) over 24 s, to 50% Solvent B (*v*/*v*) over a further 72 s and to 65% Solvent B (*v*/*v*) over a final 42 s, before flushing with 100% Solvent B for 60 s and returning to the initial column conditions to equilibrate for 60 s. The electrospray ionization source was operated in positive-ion mode (150 °C; capillary 1.5 kV; nitrogen desolvation gas 1000 L/h at 500 °C; cone gas 150 L/h) with the mass spectrometer conducting precursor ion scans.

In precursor ion scanning mode, the first quadrupole was set to scan between *m*/*z* 450–1150 before the ions were introduced to a collision cell (the second quadrupole) and fragmented with argon gas at a collision energy of 40 V. The third quadrupole was set to filter specific product ions, in this case *m/z* 135 for Adda-containing MCs and *m/z* 265 for ADMAdda-containing MCs. The precursor ions, which resulted in the specified product ions, were then determined by the MassLynx software (Version 4.1, Waters Ltd.). Following this, the samples were further de-replicated by assessing the observed retention time in comparison to the molecular weight of the precursor ions (e.g., microcystins with two arginine residues elute in an earlier retention region than other microcystin congeners and generally have masses >1000 Da) in order to compile a list of candidate MCs to be identified using conventional structural characterisation methods (described below).

The candidate MCs identified using the precursor ion scanning method were further investigated by generating MS/MS spectra for each ion of interest. Tandem MS spectra were collected in positive ion mode over an *m/z* range of 100–1200. Compounds that were presumed to be MCs containing two arginine residues (e.g., MC-RR) were fragmented using a collision energy of 25–30 V and compounds presumed to be MCs containing one or no arginine residues (e.g., MC-LR or MC-LA respectively) were fragmented using a collision energy of 40–45 V. The spectra were primarily annotated with the assistance of the software mMass [46]. When discrepancies were apparent, the spectra were annotated manually using previously published MS/MS investigations of MCs as a guide [12,27,36,47,48]. To confirm whether Dha, Mdha or Dhb were present in the MCs identified, a micro-scale thiol derivatization was performed [49]. Microcystin solutions were reacted with β-mercaptoethanol as described in Puddick et al. [50] but using the precursor ion scan described above. If no reaction had occurred within 2 h at 30 °C, the MC was classified as containing Dhb^7^. A control reaction containing MC-RR, MC-LR and MC-LA was run in parallel to confirm the reaction rate for Mdha.

DNA was extracted from each sample using the PowerSoil^®^ DNA Isolation Kit (former MO BIO laboratories, Carlsbad, CA, USA, now Qiagen, Germantown, MA, USA) for the samples collected in 2012 and the PowerBiofilm^®^ DNA Isolation Kit (former MO BIO laboratories, Carlsbad, CA, US, now Qiagen, Germantown, MA, USA) for the samples from 2013. Between 5 and 10 mg of frozen cyanobacterial material was extracted following the manufacturer’s recommendations. The resulting DNA was eluted in sterile DNAse-free water and stored frozen at −20 °C until further use. 

The DNA quality was checked by the amplification of the 16S rRNA gene using the primer set 27F/809R [22]. Several PCRs were performed on the extracted DNA, targeting genes of the *mcy*, *sxt*, and *cyr* operon involved in MC, STX, and CYN synthesis, respectively. For all reactions the Phusion™ polymerase Master Mix (NEB, Ipswich, MA, USA) or the iTaq^TM^ PCR Master Mix solution (iNtRON Biotechnology, Sangdaewon-dong, South-Korea) was used. Primers, references and PCR conditions are listed in Supplementary Table S5. Bands of interest were excised from TAE buffered 1.5% agarose-gels (*w*/*v*) using a sterile scalpel, purified with a Gel Extraction Kit (Fermentas, St. Leon-Rot, Germany) and bi-directionally sequenced using the same primer combination as for amplification on a Sanger Sequencer (3730 DNA Analyzer) at the sequencing facility GIGA (http://www.giga.uliege.be) of the University of Liège. *Microcystis aeruginosa* UAM501 served as a positive control for *mcy* genes, *Aphanizomenon ovalisporum* UAM290 for *cyr* genes, and *Aphanizomenon gracile* UAM531 for *sxt* genes [51]. The sequences obtained were aligned and manually edited using Geneious™ software (Geneious Pro 7.1.1., Biomatters Ltd., Auckland, New Zealand). Translation into the corresponding amino acid code was also done using Geneious^TM^ by aligning the amplified fragments to published and annotated *mcy* genes (of KC699835 *Nostoc* sp. 152 and AY768451 *Microcoleus* sp. PCC 8701). The closest cultured and non-cultured phylogenetic hits were identified for each sequence using the megablast and blastn tools of GenBank for nucleotide sequences and the blastp tool for amino acid sequences. Phylogenetic affiliations and accession numbers are given in Supplementary Table S3. The *sxtA* sequence was not submitted since it was represented by a single read only and can thus not be verified. 

The primer pair CYA106F 5′-CGGACGGGTGAGTAACGCGTGA-3′ and modified 519-536 5′-GTNTTACNGCGGCKGCTG-3′ were used to amplify a fragment of the 16S rRNA gene including the V2-V3 domains [52,53]. Pyrosequencing using a 454 Sequencing System (Roche 454 Life Sciences, Basel, Switzerland) was performed at the Research and Testing Laboratories (Lubbock, TE, USA) as described previously [19]. The raw 454 data can be downloaded from http://vm-lux.embl.de/~hildebra/Arctic_454/.

We used the LotuS 1.31 pipeline [54] in short amplicon mode with default quality filtering. Raw 16S rRNA gene reads were quality filtered to ensure a minimum length of 250 bp, not more than eight homonucleotides, no ambiguous bases, an average read phred quality equivalent to 25 and an accumulated error below 0.5. Clustering and denoising of OTUs was performed using UPARSE [55], removing chimeric OTUs against the RDP reference database (http://drive5.com/uchime/rdp_gold.fa) with uchime [56], merging reads with FLASH [57] and assigning a taxonomy using an RDP classifier [58]. We could assign on average 5500 ± 3108 reads to each sample that were of cyanobacterial origin. Further data analysis was conducted with R statistical language Version 3.00 (The R Foundation, https://www.r-project.org/) as described in Hildebrand et al. [59], employing the rtk software [60] for all data normalizations.

## Figures and Tables

**Table 1:** Microbial mat samples from Svalbard, the extracted mass of lyophilized material, their toxin content as determined by enzyme-linked immune sorbent assays (ELISAs), the liquid chromatography mass spectrometry (LC-MS) precursor ion screening method as well as a detailed LC-MS/MS analysis and the detection of genes involved in toxin production. Categories of the LC-MS precursor ion scan: (1) Microcystin (MC) likely to be present in the sample, (2) MC possibly present in the sample, and (3) MC absent from the sample. Genes: Non-ribosomal peptide synthetase (NRPS), polyketide synthase (PKS), microcystin gene E (*mcyE*), microcystin gene B (*mcyB*), and saxitoxin gene A (*sxtA*). Structures of MC congeners can be found in Figure 1.

Sample	Extracted Mass [g]	ELISA [µg/L]	LC-MS Precursor Ion Category (1–3)	LC-MS/MS Characterization	Genes
SV-A	0.047	n.a.	2	n.d.	NRPS, PKS
SV-B	0.078	n.a.	3	n.a.	NRPS, PKS
SV-C	0.089	n.a.	3	n.a.	-
SV-D ^N^	0.093	n.a.	1	[Asp^3^, ADMAdda^5^, Dhb^7^] MC-LR	NRPS, PKS, *mcyE*, *mcyB*
SV-E ^N^	0.068	n.a.	3	n.a.	NRPS, PKS, *mcyE*
SV-2	2.017	8	3	n.a.	NRPS, PKS
SV-8	0.679	9	3	n.a.	PKS
SV-13	0.109	14	3	n.a.	-
SV-14	0.025	0	n.a.	n.a.	NRPS, PKS
SV-16	0.105	19	3	n.a.	NRPS, PKS
SV-17	0.262	18	2	n.d.	NRPS, PKS
SV-24	1.010	2	n.a.	n.a.	-
SV-28	0.893	8	n.a.	n.a.	-
SV-39	0.020	0	n.a.	n.a.	NRPS, PKS
SV-40 ^N^	0.815	2	n.a.	n.a.	-
SV-46	2.438	6	n.a.	n.a.	-
SV-49 ^N^	0.663	54	1	[Asp^3^, ADMAdda^5^, Dhb^7^] MC-LR	-*
SV-54	1.267	>STD	3	n.a.	-
SV-56 ^N^	0.569	37	3	n.a.	-
SV-65	1.805	3	n.a.	n.a.	-
SV-74 ^N^	0.101	>STD	1	MC-LA	-*
SV-75 ^N^	0.056	>STD	1	[Asp^3^, ADMAdda^5^, Dhb^7^] MC-RR [Asp^3^, ADMAdda^5^, Dhb^7^] MC-LR	NRPS, PKS, *mcyE*, *mcyB, sxtA*
SV-77	0.256	2	n.a.	n.a.	-
SV-80 ^H^	0.104	25	1	[Asp^3^, Ser^7^] MC-RR [Asp^3^, Dha^7^] MC-RR [Asp^3^, ADMAdda^5^, Dhb^7^] MC-RR [Asp^3^, ADMAdda^5^, Thr^7^] MC-LR [Asp^3^, ADMAdda^5^, Dhb^7^] MC-LR	NRPS, PKS, *mcyE, mcyB*
SV-81 ^H,E^	11.309	n.a.	1	[Asp^3^, ADMAdda^5^, Dhb^7^] MC-LR	NRPS, PKS, *mcyE, mcyB*
SV-83 ^H^	0.126	2	1	[Asp^3^, ADMAdda^5^, Dhb^7^] MC-RR [Asp^3^, ADMAdda^5^, Dhb^7^] MC-LR Unidentified microcystin	-*

n.a. = not analyzed; n.d. = MCs not detected; >STD = above standard curve; ^N^ = sample dominated by *Nostoc*; ^H^ = Hotspring; ^E^ = endolithic; * = low DNA quality.

## Supplementary Materials

The following are available online at http://www.mdpi.com/2072-6651/10/4/147/s1, Supplemental file 1 including Figure S1: Map of Svalbard and sampling locations. Figure S2: In-situ photographs of some cyanobacterial mats sampled on Svalbard, Table S1: Metadata of samples, sampling dates, GPS coordinates and site description. Table S2: Evaluation of the precursor ion screen. Table S3: Amplified *mcyE, mcyB*, and *sxtA* sequences from cyanobacterial samples in Svalbard. Table S4: Sequences of most abundant cyanobacterial OTUs. Table S5: PCR conditions and primers. Supplemental File 2: Mass spectrometry data for all samples assessed.
