# Supplementary material for: Toxic Cyanobacteria in Svalbard: Chemical Diversity of Microcystins Detected Using a Liquid Chromatography Mass Spectrometry Precursor Ion Screening Method
Source: Toxins (Basel). 2018 Apr 3;10(4):147. doi: 10.3390/toxins10040147 (PMC5923313; doi:10.3390/toxins10040147)
Supplement: Supplementary file 1 [file toxins-10-00147-s001.zip › toxins-276582 Supp final/toxins-276582 Supp Final/toxins-276582 Supp 1 Final.pdf]

Supplementary Materials: Toxic cyanobacteria in Svalbard: Chemical diversity of microcystins detected using a liquid chromatography mass spectrometry precursor ion screening method

Julia Kleinteich, Jonathan Puddick, Susanna A. Wood, Falk Hildebrand, H. Dail Laughinghouse IV, David A. Pearce, Daniel R. Dietrich and Annick Wilmotte

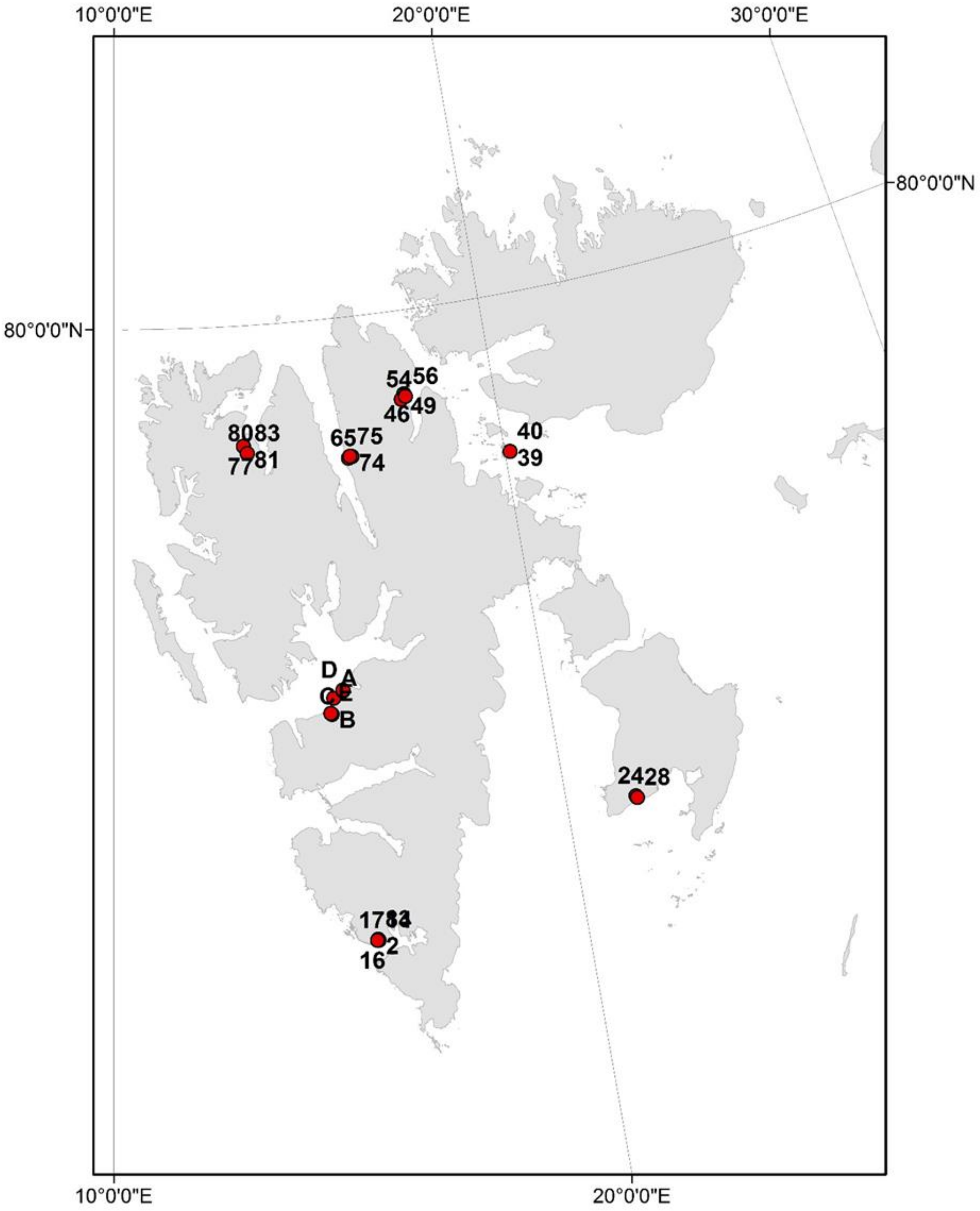

Figure S1: Map of Svalbard and sampling locations used. Numbers and letters indicate the identity and location of each sample as described in Table S1 below.

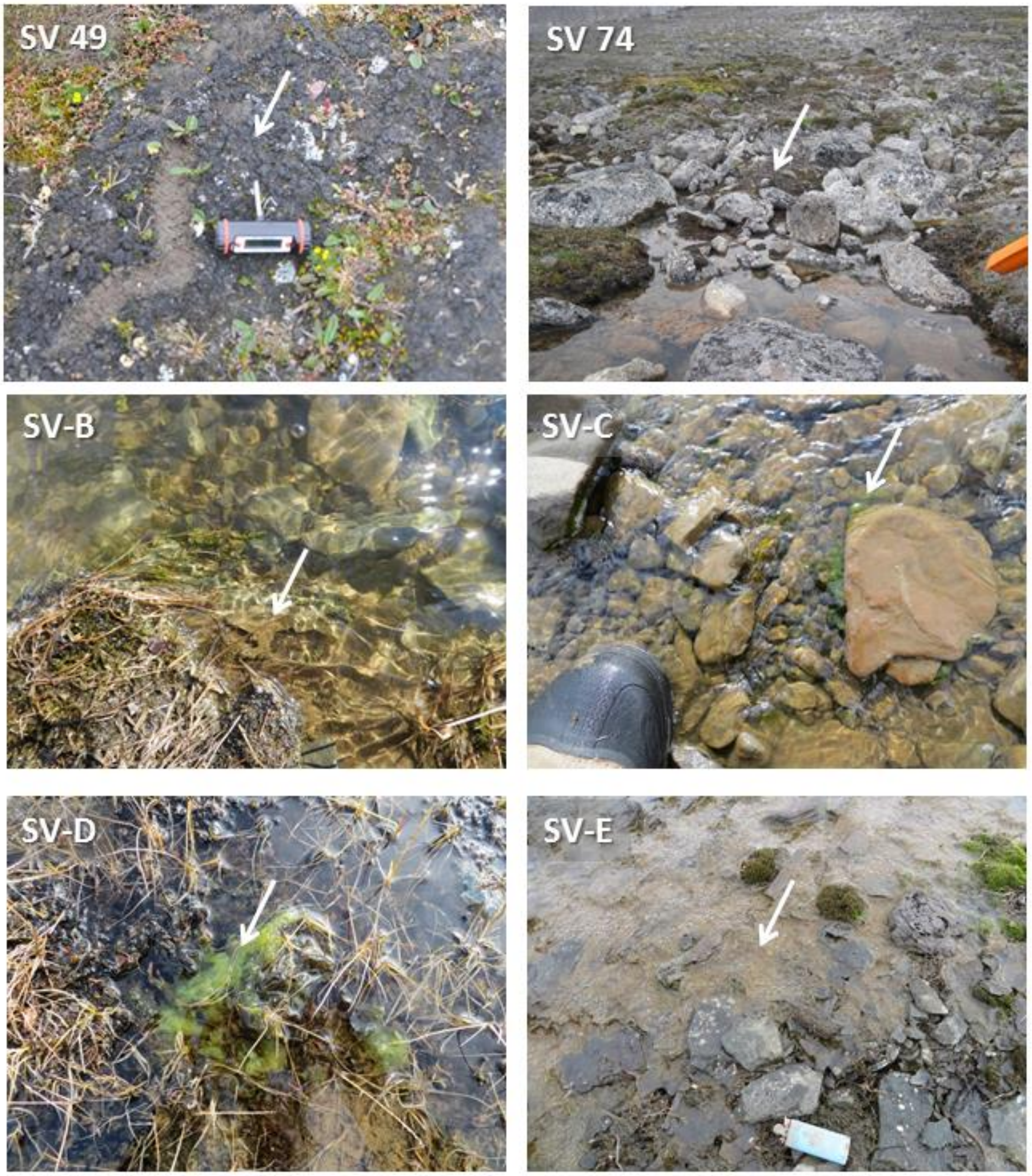

Figure S2: *In-situ* photographs of some cyanobacterial mats sampled in freshwater habitats of Svalbard. Cyanobacterial mats are indicated by arrows.

Table S1: Metadata. GPS coordinates, sampling date and description for each sample.

Sample	Sampling date	GPS coordinates	Site and sample description
SV A	7.6.2012	N 78°11′17.4′′ E 15°05′49.2′′	Colesbukta - Marshy land, Nostoc colony between moss cushions
SV B	6.6.2012	N 78°06′42.6′′ E 15°01′41.1′′	Colesbukta - Stream sample
SV C	13.6.2012	N 78°13′08.1′′ E 15°19′40.4′′	Björndalen - Stream sample, green biofilm
SV D [N]	13.6.2012	N 78°13′23.3′′ E 15°19′39.8′′	Björndalen - Marshy land, *Nostoc* colony between moss cushions
SV E [N]	7.6.2012	N 78°07′03.0′′ E 15°0′13.1′′	Colesbukta - Stream sample – orange mat
SV 2	02.07.2013	N 77°00′42.9′′ E 15°35′42.9′′	Not available
SV 8	02.07.2013	N 77°01′00.1′′ E 15°34′52.7′′	Brown mucilaginous filament
SV 13	02.07.2013	N 77°01′00.1′′ E 15°34′52.7′′	Green biofilm
SV 14	02.07.2013	N 77°00′51.1′′ E 15°35′09.9′′	Orange biofilm in small seepage
SV 16	02.07.2013	N 77°00′45.1′′ E 15°33′57.1′′	Puddle with bryophytes
SV 17	02.07.2013	N 77°00′45.1′′ E 15°33′57.1′′	Puddle embayment in stream
SV 24	07.04.2013	N 77°30′53.3′′ E 21°31′21.1′′	Red soil crust, humid, soft
SV 28	04.07.2013	N 77°30′23.3′′ E 21°32′42.6′′	Rugous dry biofilm in dry river bed
SV 39	06.07.2013	N 79°14′59.3′′ E 20°08′06.9′′	Microbial mat in swampy area
SV 40 [N]	06.07.2013	N 79°14′59.3′′ E 20°08′06.9′′	*Nostoc* soil mat
SV 46	06.07.2013	N 79°36′01.1′′ E 17°38′49.9′′	Crust, black
SV 49 [N]	06.07.2013	N 79°35′39.9′′ E 17°38′02.9′′	Wet, *Nostoc* colonies
SV 54	06.07.2013	N 79°34′31.8′′ E 17°33′36.6′′	Biofilm on the side of moraine lake
SV 56 [N]	06.07.2013	N 79°35′17.7′′ E 17°40′41.8′′	*Nostoc* communities on cliff
SV 65	07.07.2013	N 79°19′34.2′′ E 16°01′14.9′′	Lake 200×50 m; Biofilm sample (20 cm depth)
SV 74 [N]	07.07.2013	N 79°19′56.6′′ E 16°06′55.3′′	*Nostoc* in dried wetland
SV 75[N]	07.07.2013	N 79°20′02.7′′ E 16°03′46.7′′	Floating mat in wetlands; brown
SV 77	08.07.2013	N 79°25′27.2′′ E 13°20′53.3′′	Floating mat in puddle
SV 80[H]	08.07.2013	N 79°23′17.9′′ E 13°26′21.3′′	Hotspring sample
SV 81[H,E]	08.07.2013	N 79°23′17.9′′ E 13°26′21.3′′	Hotspring sample, endolithic
SV 83[H]	08.07.2013	N 79°23′17.9′′ E 13°26′21.3′′	Hotspring sample

[N] Dominated by *Nostoc* sp.; [H] Hot spring; [E] Endolithic.

Table S2: Evaluation of the microcystin precursor ion screen. Data on dereplication benefits from using the precursor scan method for identifying potential microcystins in environmental extracts. n.a. = sample not analysed in the MS/MS analysis.

**Sample**	**Number of Peaks**		**MS/MS analyses conducted**	**Microcystins identified**
	Positive ion scan	Precursor ion scan		
SV-A	12	6	1	0
SV-B	11	1	0	n.a.
SV-C	14	1	0	n.a.
SV-D	15	7	1	1
SV-E	17	10	0	n.a.
SV-02	17	4	0	n.a.
SV-08	15	1	0	n.a.
SV-13	15	3	0	n.a.
SV-16	9	6	0	n.a.
SV-17	12	3	2	0
SV-49	11	6	2	1
SV-54	11	6	0	n.a.
SV-56	8	6	0	n.a.
SV-74	7	6	3	1
SV-75	13	8	8	2
SV-80	12	7	7	5
SV-81	18	6	1	1
SV-83	16	13	7	3

Table S3: Amplified *mcyE, mcyB, and sxtA* sequences from cyanobacterial samples in Svalbard that corresponded to Category 1 in the LC-MS precursor ion screen. The length of the fragment, the closest cultured and uncultured hit in the GenBank database obtained by a BLAST (megablast for *mcyE*, blastn for *mcyB* and blastp for amino acid sequences based on nucleotide sequences translated with Geneious™), as well as the accession number of the newly obtained sequences.

**Sample**	**Sequence**	**# bp**	**Highest uncultured match, geographic origin, accession # and pairwise identity**	**Highest cultured match, geographic origin, accession # and pairwise identity**	**Highest match amino acid sequence, geographic origin, accession # and pairwise identity**	**GenBank Accession #**
**SV-D**	*mcyE*	445	Uncultured *Nostoc* sp. clone MVMG1, Antarctica, EU359046, 98%	*Nostoc* sp. 152, Finland, microcystin synthetase gene cluster, KC699835, 93%	McyE, *Nostoc* sp. 152, Finland #AGZ05272.1, 97%	MG838576
**SV-E**	*mcyE*	467	Uncultured cyanobacterium isolate *nda*F gene, Finland, EU916784, 93%	*Nostoc* sp. 152, Finland, microcystin synthetase gene cluster, KC699835, 94%	McyE, *Nostoc* sp. 152, Finland #AGZ05272.1, 97%	MG838577
**SV75**	*mcyE*	406	Uncultured *Nostoc* sp. clone MVMG1, Antarctica, EU359046, 99%	*Nostoc* sp. 152, Finland, microcystin synthetase gene cluster, KC699835, 94%	microcystin synthetase, uncultured *Nostoc* sp. Antarctica, #ABY66381.1, 99%	MG838573
**SV80**	*mcyE*	330	Uncultured *Nostoc* sp. clone MVMG1, Antarctica, EU359046, 98%	*Nostoc* sp. 152, Finland, microcystin synthetase gene cluster, KC699835, 94%	microcystin synthetase, uncultured *Nostoc* sp. Antarctica, #ABY66381.1, 99%	MG838574
**SV81**	*mcyE*	417	Uncultured cyanobacterium isolate *nda*F gene, Finland, EU916784, 93%	*Nostoc* sp. 152, Finland, microcystin synthetase gene cluster, KC699835, 97%	methyltransferase domain-containing protein, *Nostoc* sp. '*Peltigera membranacea* cyanobiont' 213, Iceland, #WP_094329291.1, 98%	MG838575
**SV-D**	*mcyB*	728	-	*Microcoleus* sp. PCC 8701 clone 1 non-ribosomal peptide synthetase gene cluster, AY768451.2, 73%	non-ribosomal peptide synthetase *Phormidesmis priestleyi*, Antarctica #WP_073072318.1, 84%	MG838581
**SV-75**	*mcyB*	756	-	*Microcoleus* sp. PCC 8701 clone 1 non-ribosomal peptide synthetase gene cluster, AY768451.2, 73%	non-ribosomal peptide synthetase *Phormidesmis priestleyi*, Antarctica #WP_073072318.1, 83%	MG838578
**SV-80**	*mcyB*	758	-	*Microcoleus* sp. PCC 8701 clone 1 non-ribosomal peptide synthetase gene cluster, AY768451.2, 71%	non-ribosomal peptide synthetase *Phormidesmis priestleyi*, Antarctica #WP_073072318.1, 84%	MG838579
**SV-81**	*mcyB*	757	-	*Microcoleus* sp. PCC 8701 clone 1 non-ribosomal peptide synthetase gene cluster, AY768451.2, 71%	non-ribosomal peptide synthetase *Phormidesmis priestleyi*, Antarctica #WP_073072318.1, 84%	MG838580
**SV75**	*sxtA*	635	Uncultured cyanobacterium clone, Arctic, JX887897, 100%	*Scytonema* sp. UCFS15 polyketide synthase (sxtA), JQ182302, 98%	-	Not submitted

Table S4: Sequences of cyanobacterial OTUs (excluding chloroplasts) derived by 454 sequencing and their highest cultured and uncultured hit on GenBank (March 2018) based on pairwise similarity as well as the geographic origin of the sequences.

OTU #	Sequence	Similar sequences in GenBank	Geographic origin
**OTU_1**	GAATCTACCTTCAGGACGGAGACAACAGTTGGAAACGACTGCTAACCCCCGATGT<br>ACCGAAAGGGCAAATATTTATAGCCTGAAGAAGAGCTCGCGTCCGATTAGCTAGT<br>TGGAGAGGTAAAAGCTCACCAAGGCGACGATCGGTAGCTGGTCTGAGAGGACGAT<br>CAGCCACACTGGGACTGAGACACGGCCCAGACTCCTACGGGAGGCAGCAGTGGG<br>GAATTTTCCGCAATGGGCGAAAGCCTGACGGAGCAAGACCGCGTGGGGAAGAAG<br>GCTCTTGGGTTGTAAACCCCTTTTCTCTGGGAAGAAAGTTGTGAAAGCAGCCTGAC<br>GGTACCAGGGGAATCAGCATCGGCTAACTCCGTGCCAGCCGCCGCCGTAAT	Uncultured bacterium clone GB7N87004INZPF, #HM731084, 99%<br>*Phormidium* sp. JR20 #KT753321, 98%	Antarctic Peninsula<br>Antarctica
**OTU_2**	GAATCTGCCTTCAGGTTGGGGACAACAGTTGGAAACGACTGCTAATACCCAATGTG<br>CCGTGAGGTGAAAGCTTTAGTGCCTGAGGATGAGCTCGCGTCTGATTAGCTAGTTG<br>GTGGGGTAATGGCCTACCAAGGCGACGATCAGTAGCTGGTCTGAGAGGATGATCA<br>GCCACACTGGGACTGAGACACGGCCCAGACTCCTACGGGAGGCAGCAGTGGGGA<br>ATTTTCCGCAATGGGCGCAAGCCTGACGGAGCAAGACCGCGTGAGGGAGGAAGG<br>CTCTTGGGTCGTAAACCTCTTTTATCATGGAAGAAGATCTGACGGTACCTGATGAA<br>TCAGCATCGGCTAACTCCGTGCCAGCAGCCGCCGCAGTAAT	Uncultured cyanobacterium ADJ921, #HQ230219.1, 99%<br>*Leptolyngbya* sp. CENA375, #KR137596, 99%	High Arctic<br>Brazil
**OTU_3**	GAATCTGCCTTCAGGTTCGGGACAACCACTGGAAACGGTGGCTAATACCGAATGT<br>GCAGAGATGTGAAATATTTATAGCCTGAAGATGAGCTCGCGTCTGATTAGCTAGTA<br>GGTGTGGTAAAAGCGCACCTAGGCGACGATCAGTAGCTGGTCTGAGAGGATGATC<br>AGCCACACTGGGACTGAGACACGGCCCAGACTCCTACGGGAGGCAGCAGTGGGG<br>AATTTTCCGCAATGGGCGAAAGCCTGACGGAGCAATACCGCGTGAGGGAGGAAG<br>GCTCTTGGGTTGTAAACCTCTTTTCTCAGGGAAGAAATCTTGACGGTACCTGAGGA<br>ATAAGCATCGGCTAACTCCGTGCCAGCAGCCGCCGTAAC	*Tolypothrix* sp. NIES-515, #LC215279, 99%<br>Uncultured cyanobacterium, #JX887893.1, 99%	Japan<br>Arctic
**OTU_4**	GAATCTACCATCAGGTCTGGGACAACCAGTGGAAACGTTGGCTAATACCAGATGT<br>GCCGAAAGGTGAAAGGTTAACTGCCTGTTGATGAGCTCGCGTCTGATTAGCTAGTT<br>GGTAGAGTAAGAGCCTACCAAGGCGACGATCAGTAGCTGGTCTGAGAGGACGATC<br>AGCCACACTGGGACTGAGACACGGCCCAGACTCCTACGGGAGGCAGCAGTGGGG<br>AATTTTCCGCAATGGGCGAAAGCCTGACGGAGCAATACCGCGTGAGGGAGGAAG<br>GCTCTTGGGTCGTAAACCTCTTTTCTCAGGGAAGAACACAATGACGGTACCTGAGG<br>AATAAGCATCGGCTGACTCCGTGCCAGCCGCCGCCGTAAC	*Nostoc pruniforme* SAG 62.79, #KM019941, 99%<br>Uncultured cyanobacterium, #JX887892.1, 99%	Denmark<br>Arctic
**OTU_5**	GAATCTGCCTTCAGGTCTGGGACAACCACTGGAAACGGTGGCTAATACCGGATGT<br>GCCGAGAGGTGAAAGGTTAACTGCCTGAAGATGAGCTCGCGTCTGATTAGCTAGTT<br>GGCAGTGTAAGAGACTACCAAGGCGACGATCAGTAGCTGGTCTGAGAGGATGATC<br>AGCCACACTGGGACTGAGACACGGCCCAGACTCCTACGGGAGGCAGCAGTGGGG<br>AATTTTCCGCAATGGGCGAAAGCCTGACGGAGCAATACCGCGTGAGGGAGGAAG<br>GCTCTTGGGTCGTAAACCTCTTTTCTCAGGGAAGAACACAATGACGGTACCTGAGG<br>AATAAGCATCGGCTAACTCCGTGCCAGCCGCCGCCGTAAG	*Nostoc* sp. Fuscopannaria pacifica UK179 cyanobiont, #KF359683, 99%<br>Uncultured *Nostoc* sp. clone K_S45, #JQ007765.1 99%	Chile<br>Scotland
**OTU_6**	GAATCTGCCTATAGGTTGGGGACAACAGTTGGAAACAATTGCTAATACCCAATGTG<br>CCGAGAGGTGAAAGCTTTAGTGCCTGTAGATGAGCTCGCGTCCGATTAGCTAGTTG<br>GTTGGGTAATGGCTGACCAAGGCGACGATCGGTAGCTGGTCTGAGAGGATGATCA<br>GCCACACTGGGACTGAGACACGGCCCAGACTCCTACGGGAGGCAGCAGTGGGGA<br>ATTTTCCGCAATGGGCGAAAGCCTGACGGAGCAATACCGCGTGCGGGACGAAGGC<br>TTTTGGGTCGTAAACCGCTTTTGTTAGGGAAGAAGAACTGACGGTACCTAACGAAT<br>CAGCATCGGCTAACTCCGTGCCAGCAGCCGCCGTAAT	Uncultured cyanobacterium #JX887887.1, 99%<br>Cyanobacterium cCLA-17 #HQ230228, 98%	Arctic<br>High Arctic

Table S5: PCR conditions and primers.

Primer	Gene	Sequence	T_m	Reference
27F	16S rRNA	AGAGTTTGATCCTGGCTCAG	54°C*	(1)
809R	16S rRNA	GCTTCGGCACGGCTCGGGTCGATA		
HEPF	*mcyE*	TTTGGGGTTAACTTTTTTGGGCATAGTC	60°C / 56°C*	(1)
HEPR	*mcyE*	AATTCTTGAGGCTGTAAATCGGGTTT		
FAA	*mcyB*	CTATGTTATTTATACATCAGG	48°C	(2)
RAA	*mcyB*	CTCAGCTTAACTTGATTATC		
MTF mod	*NRPS*	GCNGGDGGRGCNTAYGTNCC	54°C	modified after (2)
MTR mod	*NRPS*	CCNCGAAATYTTRACYTG		
DKF	PKS	GTGCCGGTNCCRTGNGYYTC	61.5°C	(3)
DKR	PKS	GCGATGGAYCCNCARCARMG		
Cyl M13	*cyrB*	GGCAAATTGTGATAGCCACGAGC	50°C*	(4)
Cyl M14	*cyrB*	GATGGAACATCGCTCACTGGTG		
sxtaF	*sxtA*	GCGTACATCCAAGCTGGACTCG	62.0°C	(5)
sxtaR	*sxtA*	GTAGTCCAGCTAAGGCACTTGC		

*PCR using iTaq™ PCR Master Mix solution (iNtRON Biotechnology, Korea), all other reactions using Phusion Master Mix

1. Jungblut, A.D.; Neilan, B.A. Molecular identification and evolution of the cyclic peptide hepatotoxins, microcystin and nodularin, synthetase genes in three orders of cyanobacteria. *Arch. Microbiol.* **2006**, *185*, 107–114.
2. Neilan, B.A.; Dittmann, E.; Rouhiainen, L.; Bass, R.A.; Schaub, V.; Sivonen, K.; Börner, T. Nonribosomal Peptide Synthesis and Toxigenicity of Cyanobacteria. *J. Bacteriol.* **1999**, *181*, 4089–4097.
3. Moffitt, M.C.; Neilan, B.A. Evolutionary affiliations within the superfamily of ketosynthases reflect complex pathway associations. *J. Mol. Evol.* **2003**, *56*, 446–457.
4. Schembri, M.A.; Neilan, B.A.; Saint, C.P. Identification of genes implicated in toxin production in the cyanobacterium *Cylindrospermopsis raciborskii*. *Environ. Toxicol.* **2001**, *16*, 413–421.
5. Ballot, A.; Fastner, J.; Wiedner, C. Paralytic shellfish poisoning toxin-producing cyanobacterium *Aphanizomenon gracile* in Northeast Germany. *Appl. Environ. Microbiol.* **2010**, *76*, 1173–1180.
