# Supplementary material for: Toxic Cyanobacteria in Svalbard: Chemical Diversity of Microcystins Detected Using a Liquid Chromatography Mass Spectrometry Precursor Ion Screening Method
Source: Toxins (Basel). 2018 Apr 3;10(4):147. doi: 10.3390/toxins10040147 (PMC5923313; doi:10.3390/toxins10040147)
Supplement: Supplementary file 1 [file toxins-10-00147-s001.zip › toxins-276582 Supp final/toxins-276582 Supp Final/toxins-276582 Supp 2 Final.pdf]

Supplementary Materials: Toxic cyanobacteria in Svalbard: Chemical diversity of microcystins detected using a liquid chromatography mass spectrometry precursor ion screening method

Julia Kleinteich, Jonathan Puddick, Susanna A. Wood, Falk Hildebrand, H. Dail Laughinghouse IV, David A. Pearce, Daniel R. Dietrich and Annick Wilmotte

Environmental Sample SV-A

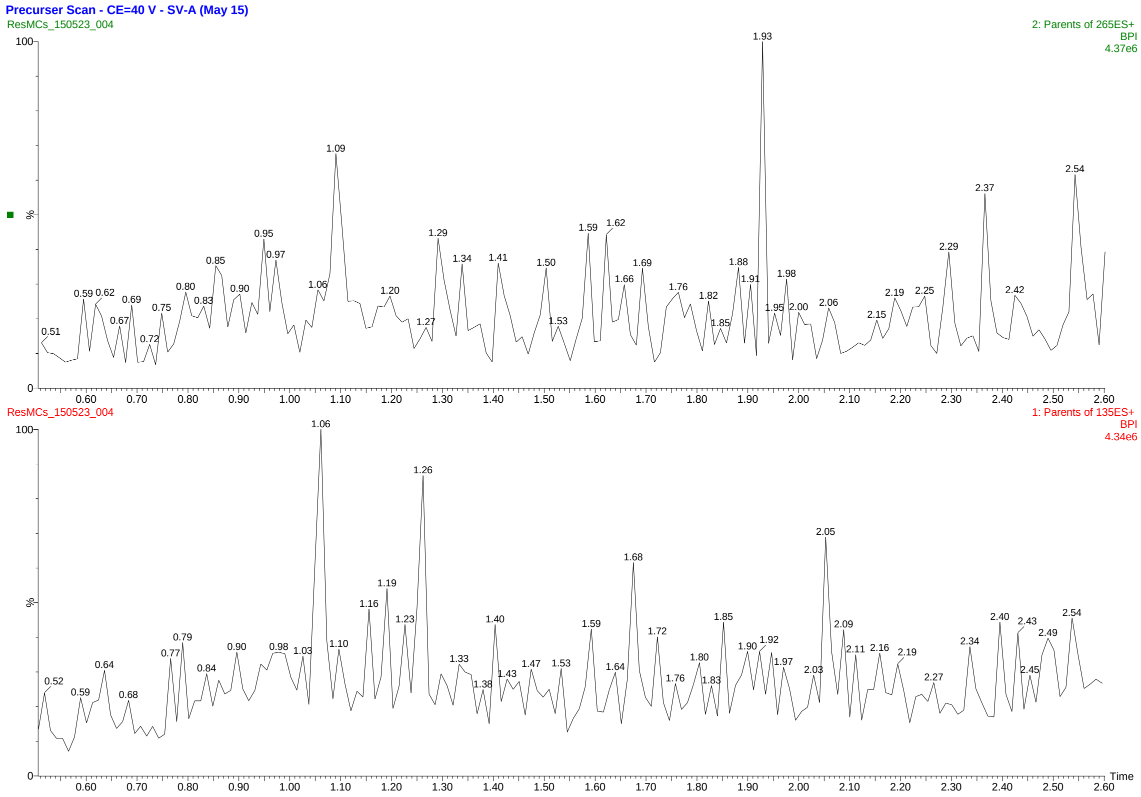

Base-peak chromatogram of the precursor ion scan for SV-A (CE=40 V).

RT[a] (min)	Prod.[b]	*m/z*[c]	M_r[d] (Da)	Identity	Comments
1.06	135	744.4	-	Unknown	Unlikely to be a microcystin because of retention time in conjunction with *m/z*.
1.09	265	563.8	-	Unknown	Insufficient signal in MS/MS spectrum.
1.26	135	461.6	-	Unknown	Unlikely to be a microcystin because of retention time in conjunction with *m/z*.
1.68	135	484.9	-	Unknown	Unlikely to be a microcystin because of retention time in conjunction with *m/z*.
1.93	265	733.5	-	Unknown	Unlikely to be a microcystin because of retention time in conjunction with *m/z*.
2.05	135	645.0	-	Unknown	Unlikely to be a microcystin because of retention time in conjunction with *m/z*.

[a] RT = Retention time on a Waters Acquity BEH-C_{18} UPLC column (2.1×50 mm; 1.7-µm). [b] Product ion trace in which the precursor ion was identified (m/z 135 for Adda-containing compounds or m/z 265 for ADMAdda-containing compounds). [c] *m/z* = Mass-to-charge ratio of precursor ion. [d] M_r = Molecular weight of the identified precursor ion.

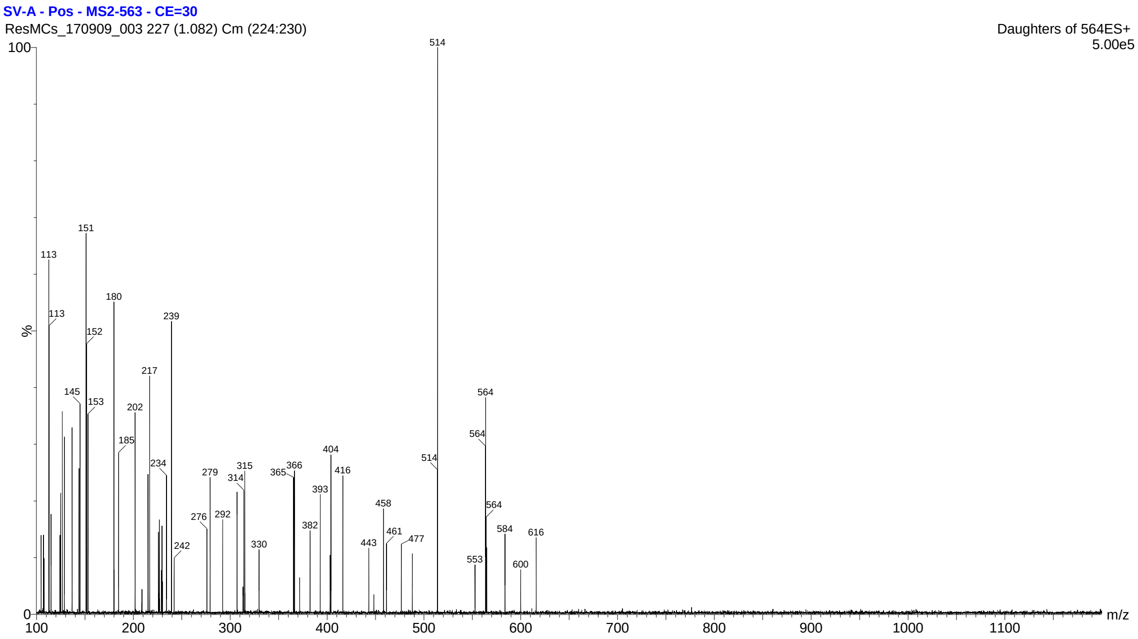

MS/MS spectrum of the 563.8 ion at 1.09 min from SV-A (CE=30 V).

Environmental Sample SV-B

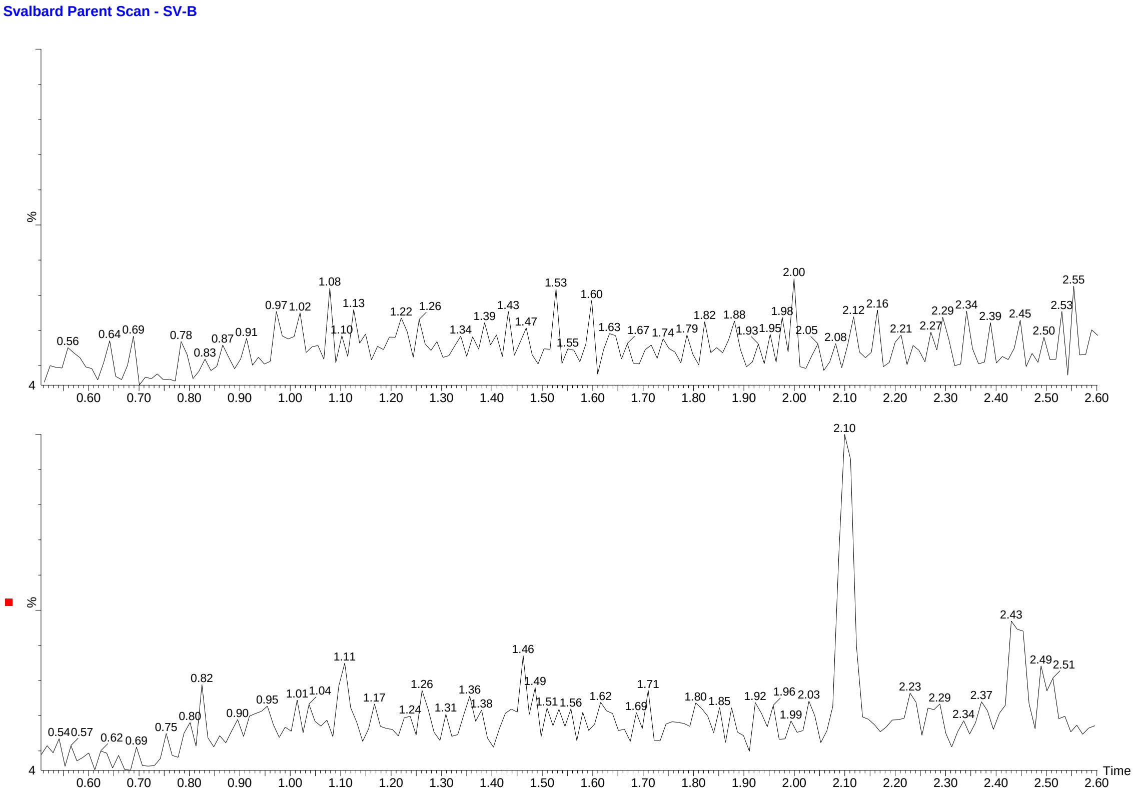

Base-peak chromatogram of the precursor ion scan for SV-B (CE=40 V).

RT[a] (min)	Prod.[b]	*m/z*[c]	M_r[d] (Da)	Identity	Comments
2.10	265	666.3	-	Unknown	Unlikely to be a microcystin because of retention time in conjunction with *m/z*.

[a] RT = Retention time on a Waters Acquity BEH-C_{18} UPLC column (2.1×50 mm; 1.7-µm). [b] Product ion trace in which the precursor ion was identified (*m/z* 135 for Adda-containing compounds or *m/z* 265 for ADMAdda-containing compounds). [c] *m/z* = Mass-to-charge ratio of precursor ion. [d] M_r = Molecular weight of the identified precursor ion.

Environmental Sample SV-C

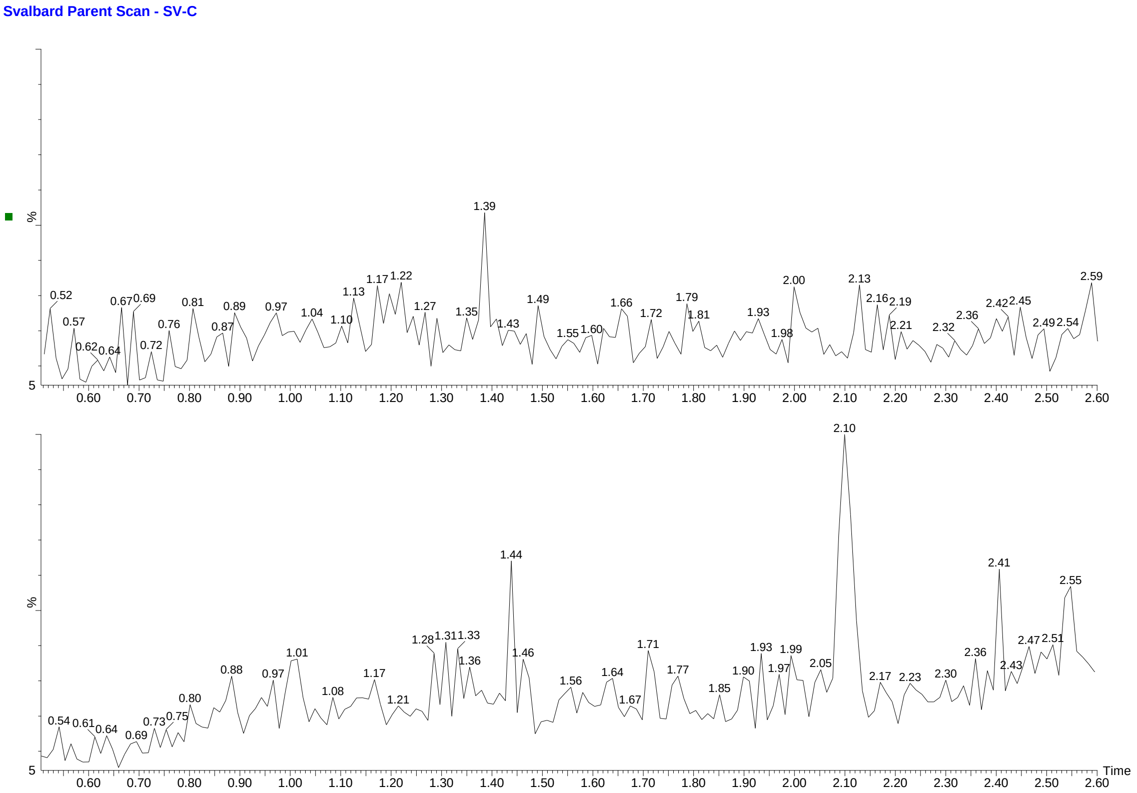

Base-peak chromatogram of the precursor ion scan for SV-C (CE=40 V).

RT[a] (min)	Prod.[b]	*m/z*[c]	M_r[d] (Da)	Identity	Comments
2.10	265	666.1	-	Unknown	Unlikely to be a microcystin because of retention time in conjunction with *m/z*.

[a] RT = Retention time on a Waters Acquity BEH-C_{18} UPLC column (2.1×50 mm; 1.7-µm). [b] Product ion trace in which the precursor ion was identified (*m/z* 135 for Adda-containing compounds or *m/z* 265 for ADMAdda-containing compounds). [c] *m/z* = Mass-to-charge ratio of precursor ion. [d] M_r = Molecular weight of the identified precursor ion.

Environmental Sample SV-D

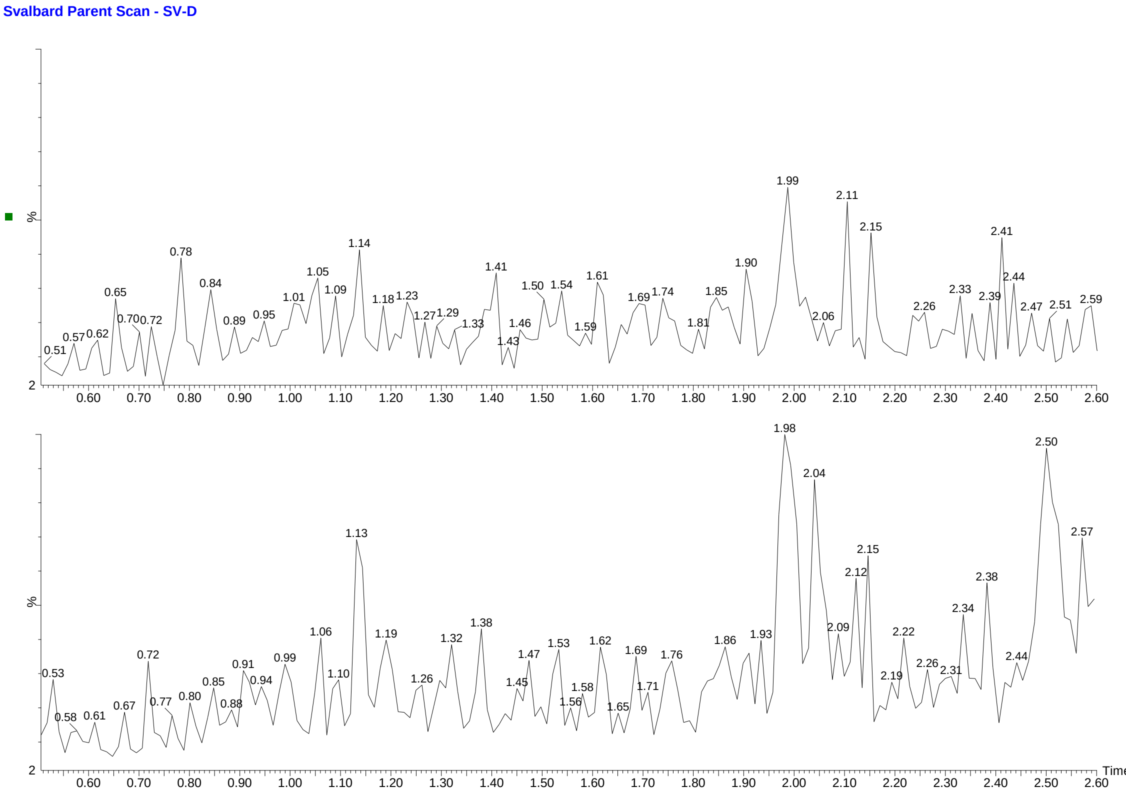

Base-peak chromatogram of the precursor ion scan for SV-D (CE=40 V).

RT[a] (min)	Prod.[b]	*m*/*z*[c]	M_r[d] (Da)	Identity	Comments
1.13	265	526.4	1051.6	[Asp[3], ADMAdda[5], Dhb[7]] MC-RR	MS/MS used to confirm identity. Thiol derivatisation confirmed presence of Dhb.
1.98	265	575.2	-	Unknown	Unlikely to be a microcystin because of retention time in conjunction with *m*/*z*.
1.99	135	575.2	-	Unknown	Unlikely to be a microcystin because of retention time in conjunction with *m*/*z*.
2.04	265	575.2	-	Unknown	Unlikely to be a microcystin because of retention time in conjunction with *m*/*z*.
2.11	135	557.5	-	Unknown	Unlikely to be a microcystin because of retention time in conjunction with *m*/*z*.
2.15	135	557.7	-	Unknown	Unlikely to be a microcystin because of retention time in conjunction with *m*/*z*.
2.50	265	846.3	-	Unknown	Unlikely to be a microcystin because of retention time in conjunction with *m*/*z*.

[a] RT = Retention time on a Waters Acquity BEH-C_{18} UPLC column (2.1×50 mm; 1.7-µm). [b] Product ion trace in which the precursor ion was identified (*m*/*z* 135 for Adda-containing compounds or *m*/*z* 265 for ADMAdda-containing compounds). [c] *m*/*z* = Mass-to-charge ratio of precursor ion. [d] M_r = Molecular weight of the identified precursor ion.

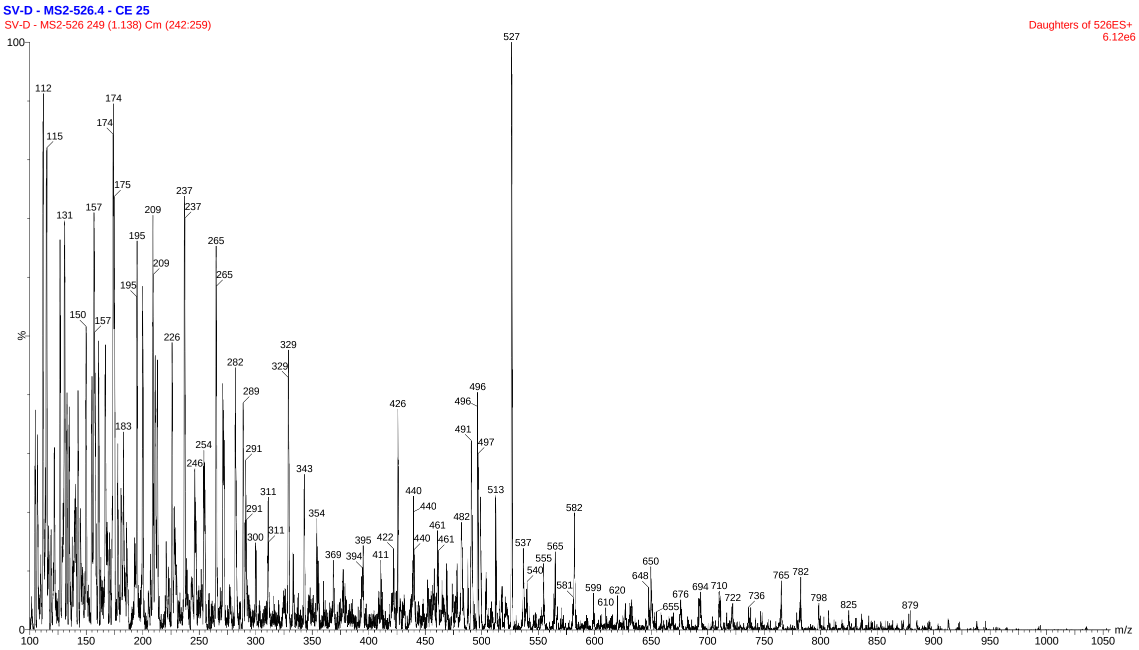

MS/MS spectrum of the 526.4 $[M+2H]^{2+}$ ion at 1.14 min from SV-D (CE=25-30 V).

Environmental Sample SV-E

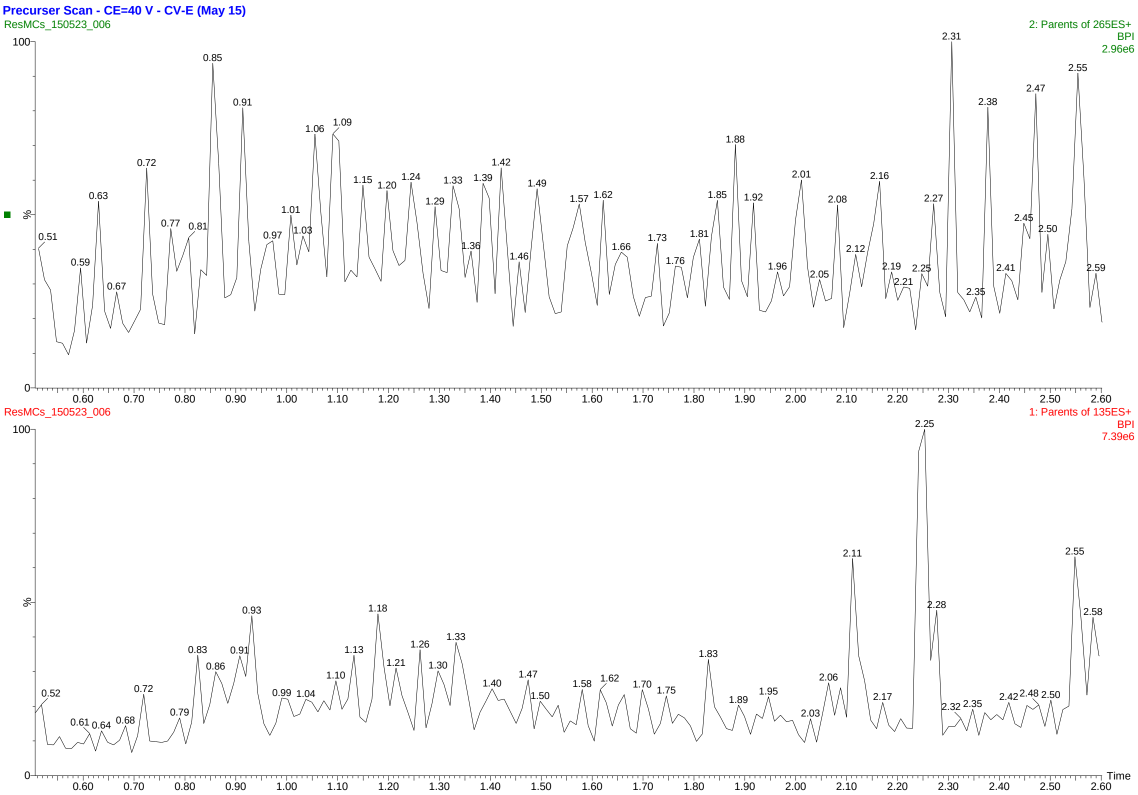

Base-peak chromatogram of the precursor ion scan for SV-E (CE=40 V).

RT[a] (min)	Prod.[b]	*m*/*z*[c]	M_r[d] (Da)	Identity	Comments
0.85	265	594.1	-	Unknown	Unlikely to be a microcystin because of retention time in conjunction with *m*/*z*.
0.91	265	665.1	-	Unknown	Unlikely to be a microcystin because of retention time in conjunction with *m*/*z*.
2.11	135	666.2	-	Unknown	Unlikely to be a microcystin because of retention time in conjunction with *m*/*z*.
2.25	135	666.3	-	Unknown	Unlikely to be a microcystin because of retention time in conjunction with *m*/*z*.
2.28	135	559.0	-	Unknown	Unlikely to be a microcystin because of retention time in conjunction with *m*/*z*.
2.31	265	675.4	-	Unknown	Unlikely to be a microcystin because of retention time in conjunction with *m*/*z*.
2.38	265	581.8	-	Unknown	Unlikely to be a microcystin because of retention time in conjunction with *m*/*z*.
2.47	265	650.7	-	Unknown	Unlikely to be a microcystin because of retention time in conjunction with *m*/*z*.
2.55	135	504.0	-	Unknown	Unlikely to be a microcystin because of retention time in conjunction with *m*/*z*.
2.55	265	957.0	-	Unknown	Unlikely to be a microcystin because of retention time in conjunction with *m*/*z*.

[a] RT = Retention time on a Waters Acquity BEH-C_{18} UPLC column (2.1×50 mm; 1.7-µm). [b] Product ion trace in which the precursor ion was identified (*m*/*z* 135 for Adda-containing compounds or *m*/*z* 265 for ADMAdda-containing compounds). [c] *m*/*z* = Mass-to-charge ratio of precursor ion. [d] M_r = Molecular weight of the identified precursor ion.

Environmental Sample SV-02

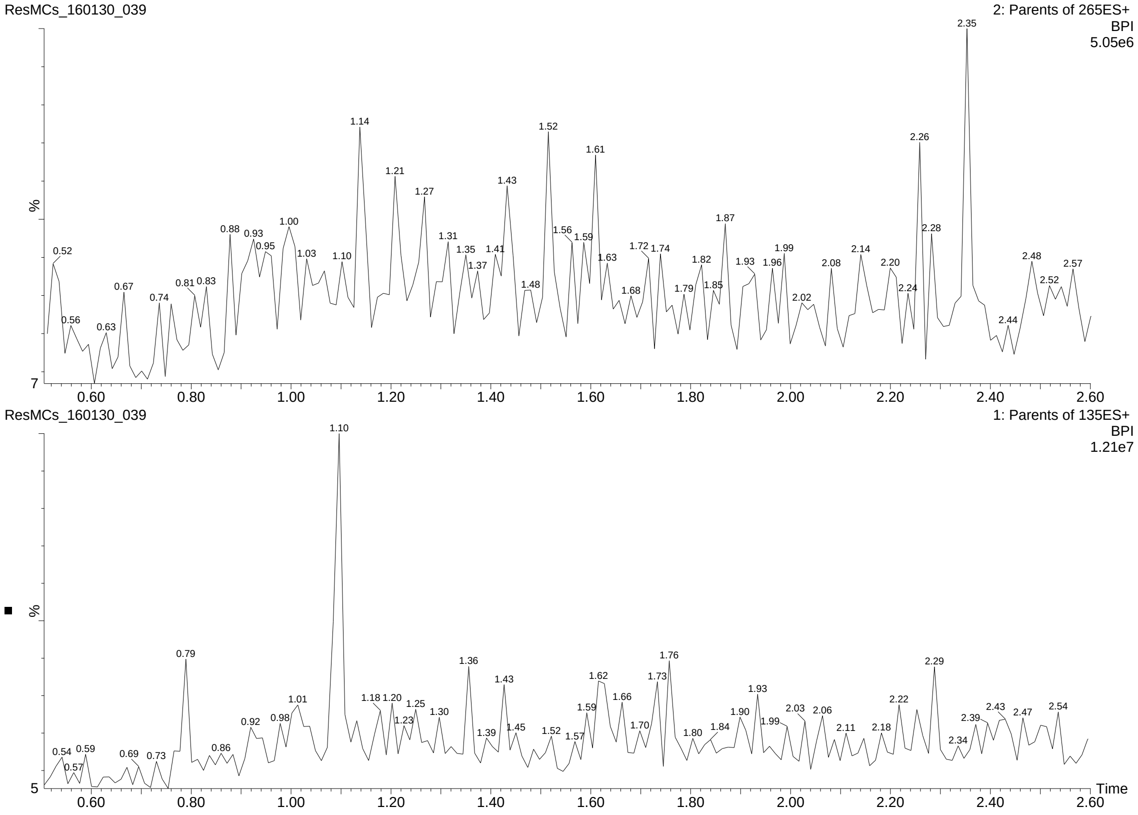

Base-peak chromatogram of the precursor ion scan for SV-02 (CE=40 V).

RT[a] (min)	Prod.[b]	*m*/*z*[c]	M_r[d] (Da)	Identity	Comments
0.79	135	636.0	-	Unknown	Unlikely to be a microcystin because of retention time in conjunction with *m*/*z*.
1.10	135	837.5	-	Unknown	Unlikely to be a microcystin because of retention time in conjunction with *m*/*z*.
1.14	265	615.7	-	Unknown	Unlikely to be a microcystin because of retention time in conjunction with *m*/*z*.
2.35	265	747.2	-	Unknown	Unlikely to be a microcystin because of retention time in conjunction with *m*/*z*.

[a] RT = Retention time on a Waters Acquity BEH-C_{18} UPLC column (2.1×50 mm; 1.7-µm). [b] Product ion trace in which the precursor ion was identified (*m*/*z* 135 for Adda-containing compounds or *m*/*z* 265 for ADMAdda-containing compounds). [c] *m*/*z* = Mass-to-charge ratio of precursor ion. [d] M_r = Molecular weight of the identified precursor ion.

Environmental Sample SV-08

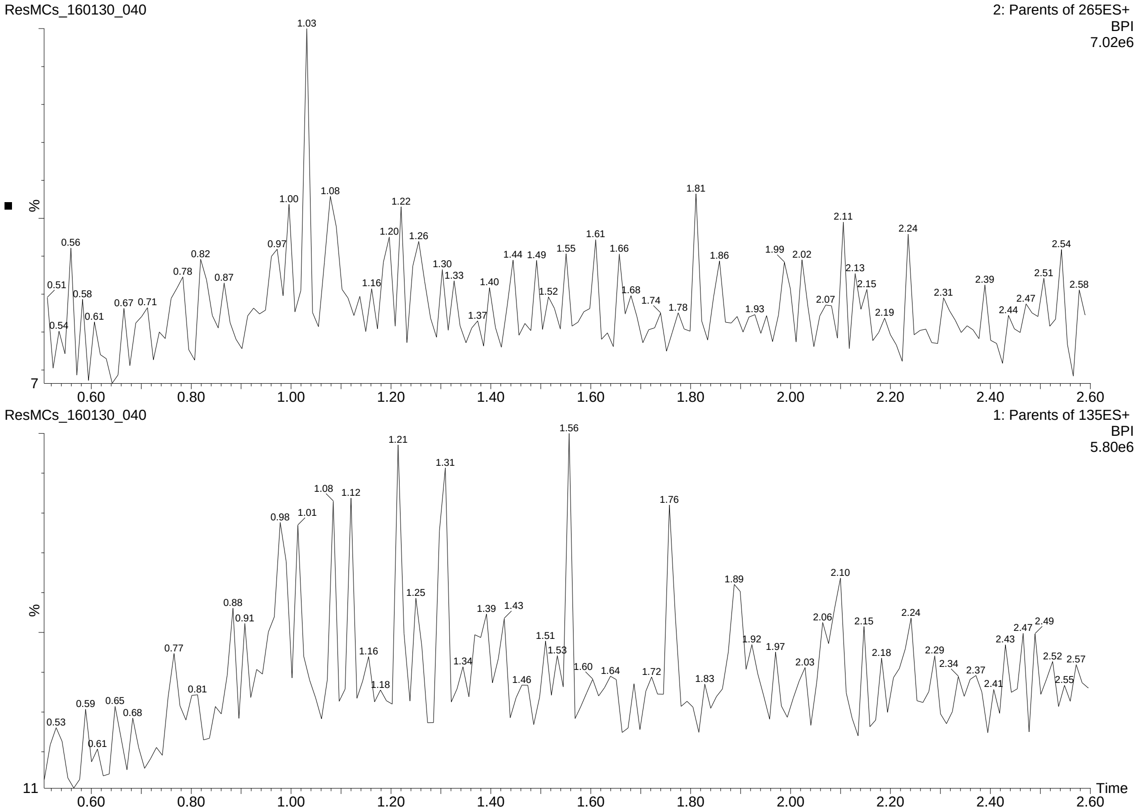

Base-peak chromatogram of the precursor ion scan for SV-08 (CE=40 V).

RT[a] (min)	Prod.[b]	*m/z*[c]	M_r[d] (Da)	Identity	Comments
1.03	265	453.6	-	Unknown	Unlikely to be a microcystin because of retention time in conjunction with *m/z*.

[a] RT = Retention time on a Waters Acquity BEH-C_{18} UPLC column (2.1×50 mm; 1.7-µm). [b] Product ion trace in which the precursor ion was identified (*m/z* 135 for Adda-containing compounds or *m/z* 265 for ADMAdda-containing compounds). [c] *m/z* = Mass-to-charge ratio of precursor ion. [d] M_r = Molecular weight of the identified precursor ion.

Environmental Sample SV-13

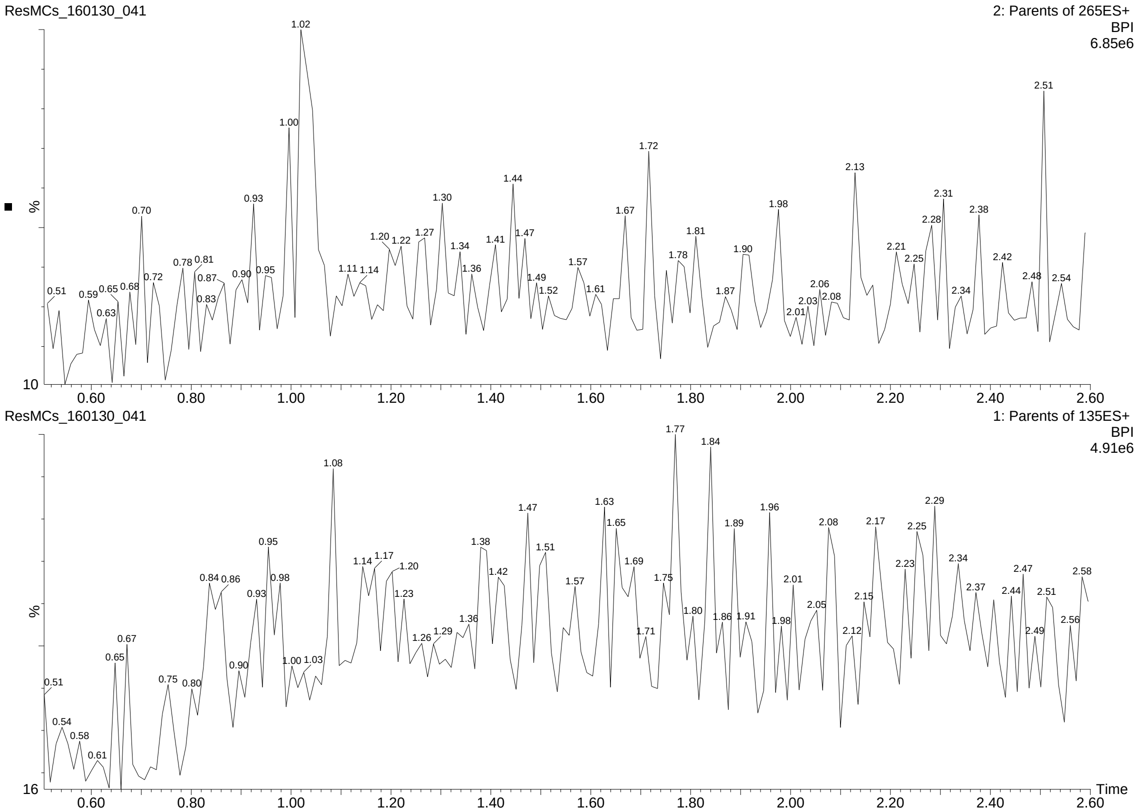

Base-peak chromatogram of the precursor ion scan for SV-13 (CE=40 V).

RT[a] (min)	Prod.[b]	*m/z*[c]	M_r[d] (Da)	Identity	Comments
1.02	265	452.7	-	Unknown	Unlikely to be a microcystin because of retention time in conjunction with *m/z*.
1.08	135	701.5	-	Unknown	Unlikely to be a microcystin because of retention time in conjunction with *m/z*.
1.77	135	630.7	-	Unknown	Unlikely to be a microcystin because of retention time in conjunction with *m/z*.

[a] RT = Retention time on a Waters Acquity BEH-C_{18} UPLC column (2.1×50 mm; 1.7-µm). [b] Product ion trace in which the precursor ion was identified (*m/z* 135 for Adda-containing compounds or *m/z* 265 for ADMAdda-containing compounds). [c] *m/z* = Mass-to-charge ratio of precursor ion. [d] M_r = Molecular weight of the identified precursor ion.

Environmental Sample SV-16

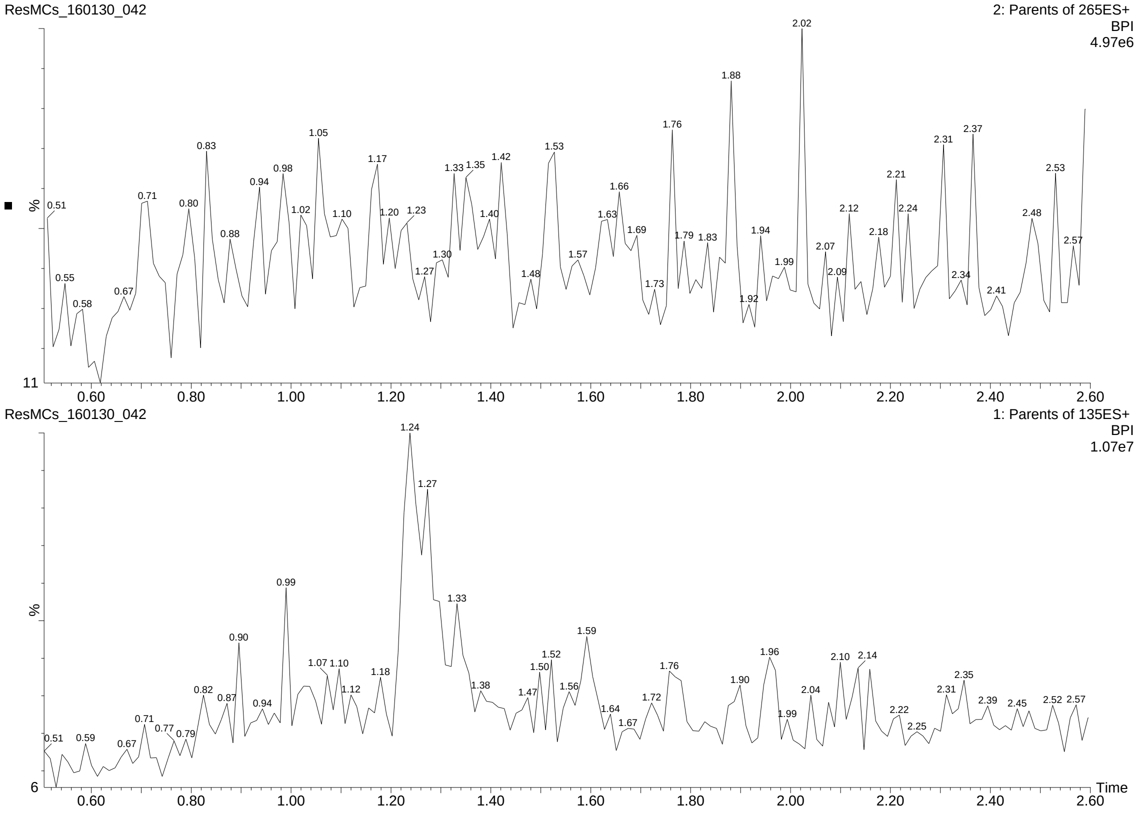

Base-peak chromatogram of the precursor ion scan for SV-16 (CE=40 V).

RT[a] (min)	Prod.[b]	*m*/*z*[c]	M_r[d] (Da)	Identity	Comments
0.99	135	752.7	-	Unknown	Unlikely to be a microcystin because of retention time in conjunction with *m*/*z*.
1.24	135	604.8	-	Unknown	Unlikely to be a microcystin because of retention time in conjunction with *m*/*z*.
1.53	265	863.9	-	Unknown	Unlikely to be a microcystin because of retention time in conjunction with *m*/*z*.
1.76	265	827.2	-	Unknown	Unlikely to be a microcystin because of retention time in conjunction with *m*/*z*.
1.88	265	818.9	-	Unknown	Unlikely to be a microcystin because of retention time in conjunction with *m*/*z*.
2.02	265	817.4	-	Unknown	Unlikely to be a microcystin because of retention time in conjunction with *m*/*z*.

[a] RT = Retention time on a Waters Acquity BEH-C_{18} UPLC column (2.1×50 mm; 1.7-µm). [b] Product ion trace in which the precursor ion was identified (*m*/*z* 135 for Adda-containing compounds or *m*/*z* 265 for ADMAdda-containing compounds). [c] *m*/*z* = Mass-to-charge ratio of precursor ion. [d] M_r = Molecular weight of the identified precursor ion.

Environmental Sample SV-17

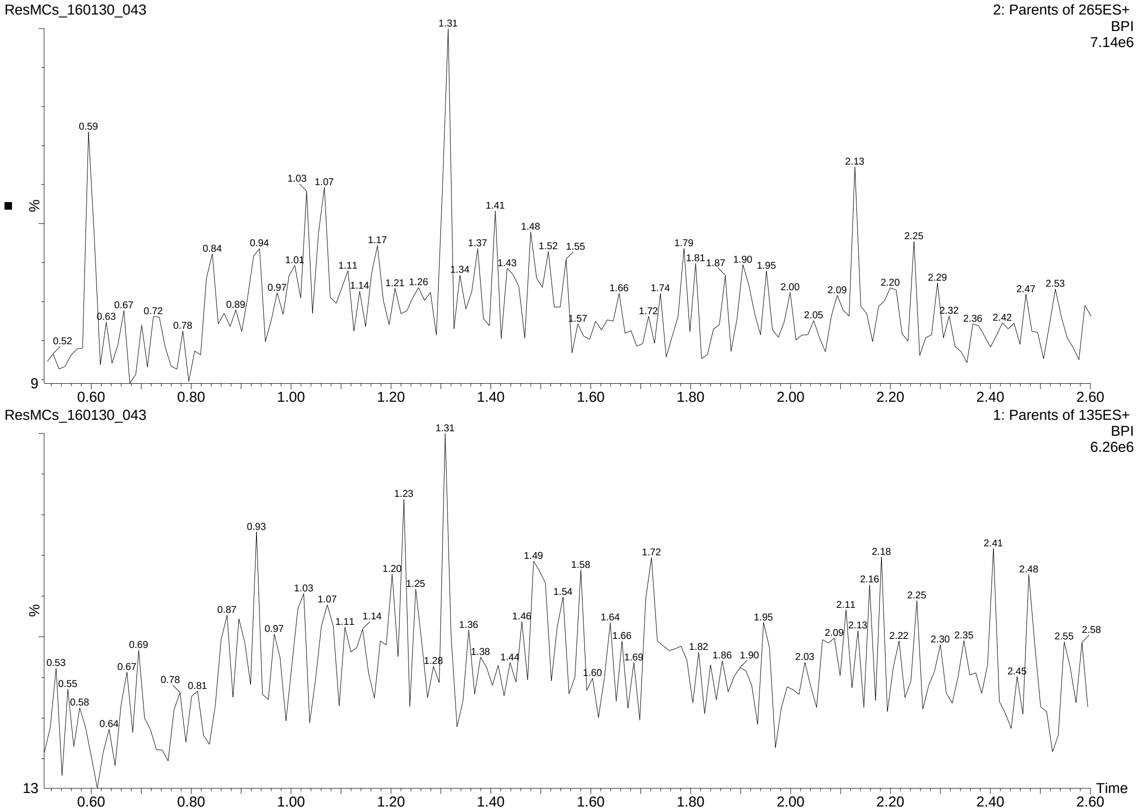

Base-peak chromatogram of the precursor ion scan for SV-17 (CE=40 V).

RT[a] (min)	Prod.[b]	*m*/*z*[c]	M_r[d] (Da)	Identity	Comments
1.31	265	757.5	-	Unknown	Unlikely to be a microcystin because of retention time in conjunction with *m*/*z*.
1.31	135	1070.9	-	Unknown	Insufficient signal in MS/MS spectrum.
2.13	265	933.7	-	Unknown	Insufficient signal in MS/MS spectrum.

[a] RT = Retention time on a Waters Acquity BEH-C_{18} UPLC column (2.1×50 mm; 1.7-μm). [b] Product ion trace in which the precursor ion was identified (*m*/*z* 135 for Adda-containing compounds or *m*/*z* 265 for ADMAdda-containing compounds). [c] *m*/*z* = Mass-to-charge ratio of precursor ion. [d] M_r = Molecular weight of the identified precursor ion.

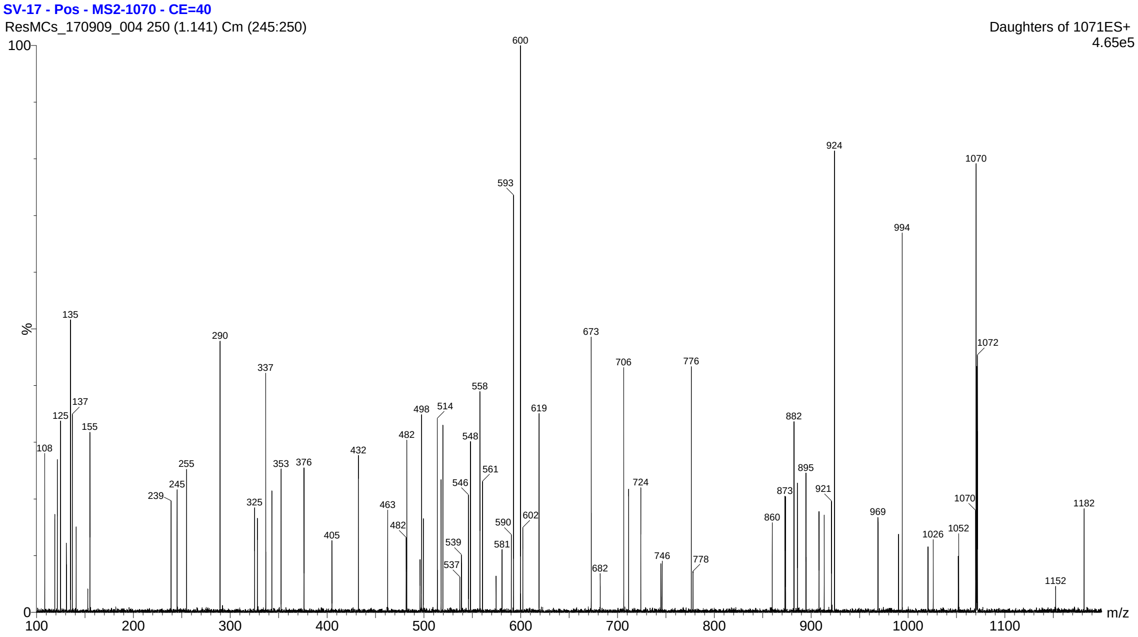

MS/MS spectrum of the 1070.9 ion at 1.13 min from SV-17 (CE=40 V).

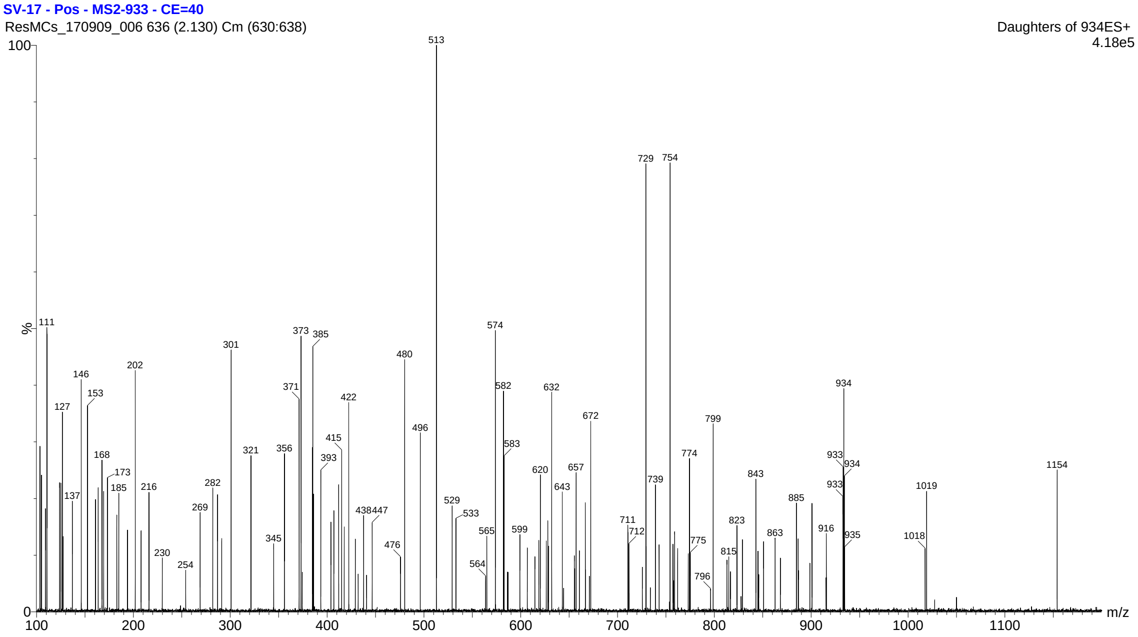

MS/MS spectrum of the 933.7 ion at 2.13 min from SV-17 (CE=40 V).

Environmental Sample SV-49

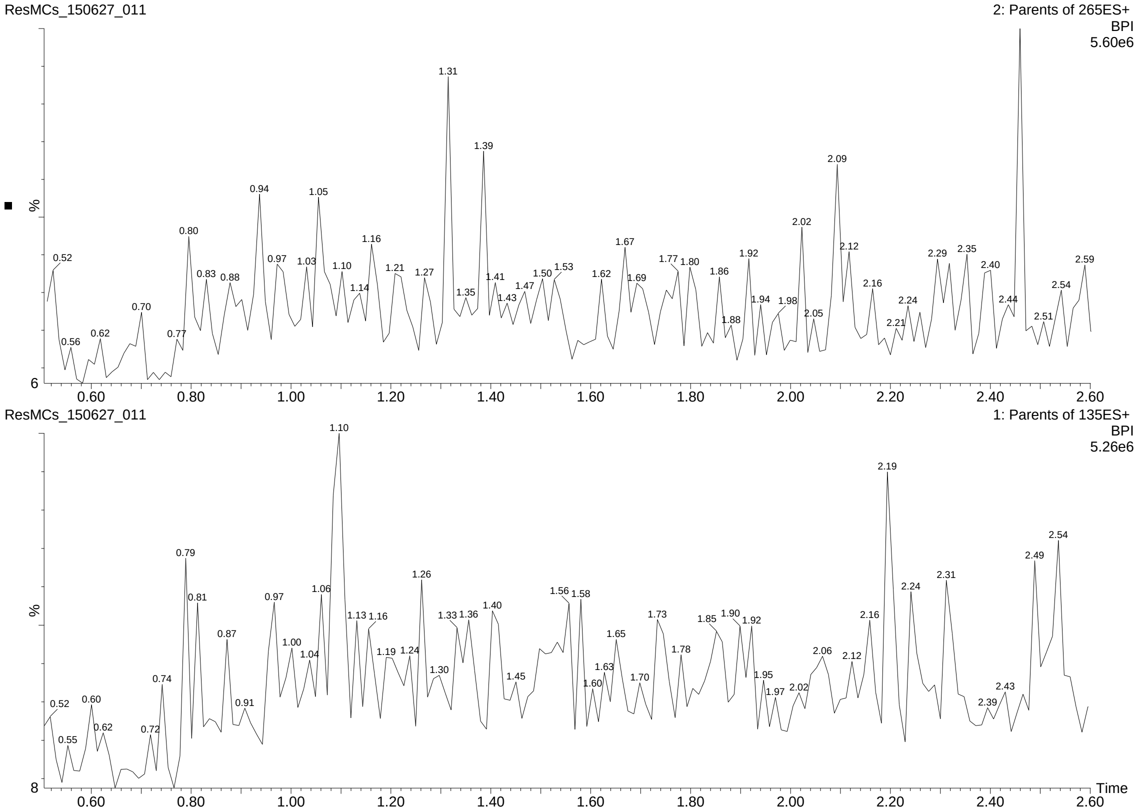

Base-peak chromatogram of the precursor ion scan for SV-49 (CE=40 V).

RT[a] (min)	Prod.[b]	*m*/*z*[c]	M_r[d] (Da)	Identity	Comments
1.10	135	837.9	-	Unknown	Unlikely to be a microcystin because of retention time in conjunction with *m*/*z*.
1.31	265	577.7	-	Unknown	Unlikely to be a microcystin because of retention time in conjunction with *m*/*z*.
1.39	265	1009.6	1008.5	[Asp³, ADMAdda⁵, Dhb⁷] MC-LR	MS/MS used to confirm identity. Thiol derivatisation confirmed presence of Dhb.
2.09	265	709.8	-	Unknown	Unlikely to be a microcystin because of retention time in conjunction with *m*/*z*.
2.19	135	1012.9	-	Unknown	Insufficient signal in MS/MS spectrum.
2.46	265	597.6	-	Unknown	Unlikely to be a microcystin because of retention time in conjunction with *m*/*z*.

[a] RT = Retention time on a Waters Acquity BEH-C_{18} UPLC column (2.1×50 mm; 1.7-µm). [b] Product ion trace in which the precursor ion was identified (*m*/*z* 135 for Adda-containing compounds or *m*/*z* 265 for ADMAdda-containing compounds). [c] *m*/*z* = Mass-to-charge ratio of precursor ion. [d] M_r = Molecular weight of the identified precursor ion.

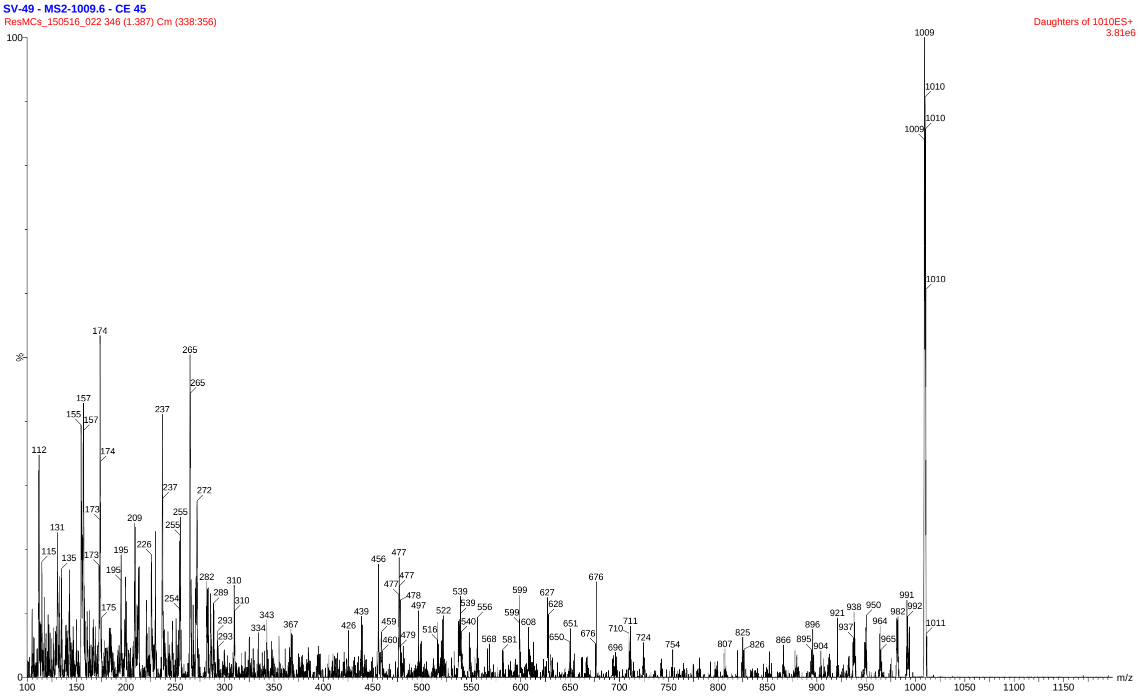

MS/MS spectrum of the 1009.6 [M+H]$^+$ ion at 1.39 min from SV-49 (CE=45 V).

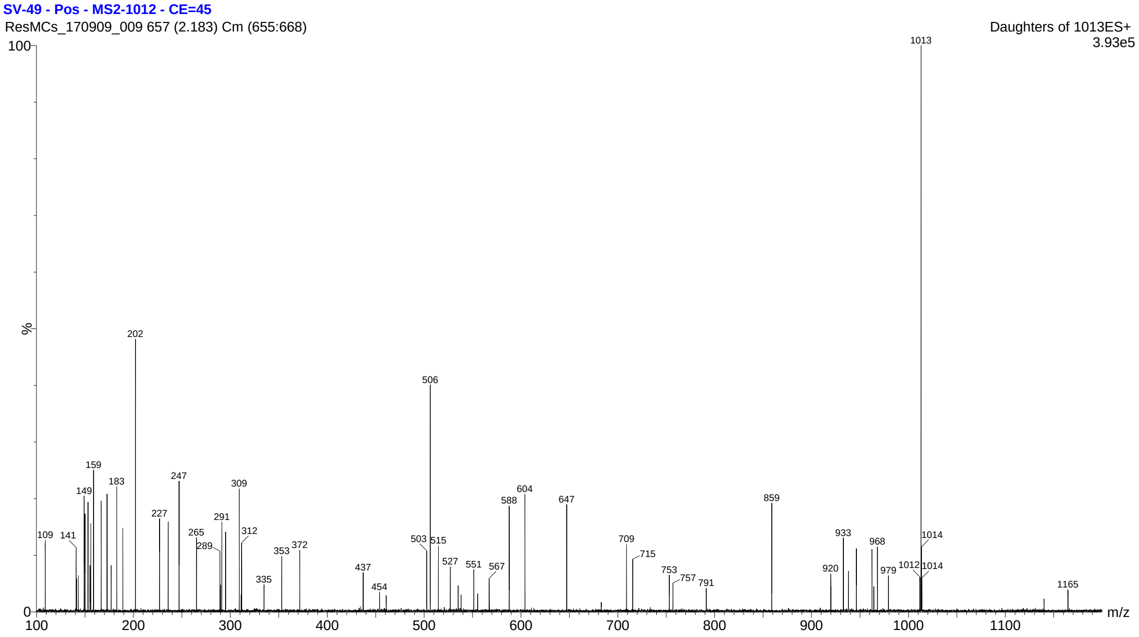

MS/MS spectrum of the 1012.9 ion at 2.19 min from SV-49 (CE=45 V).

Environmental Sample SV-54

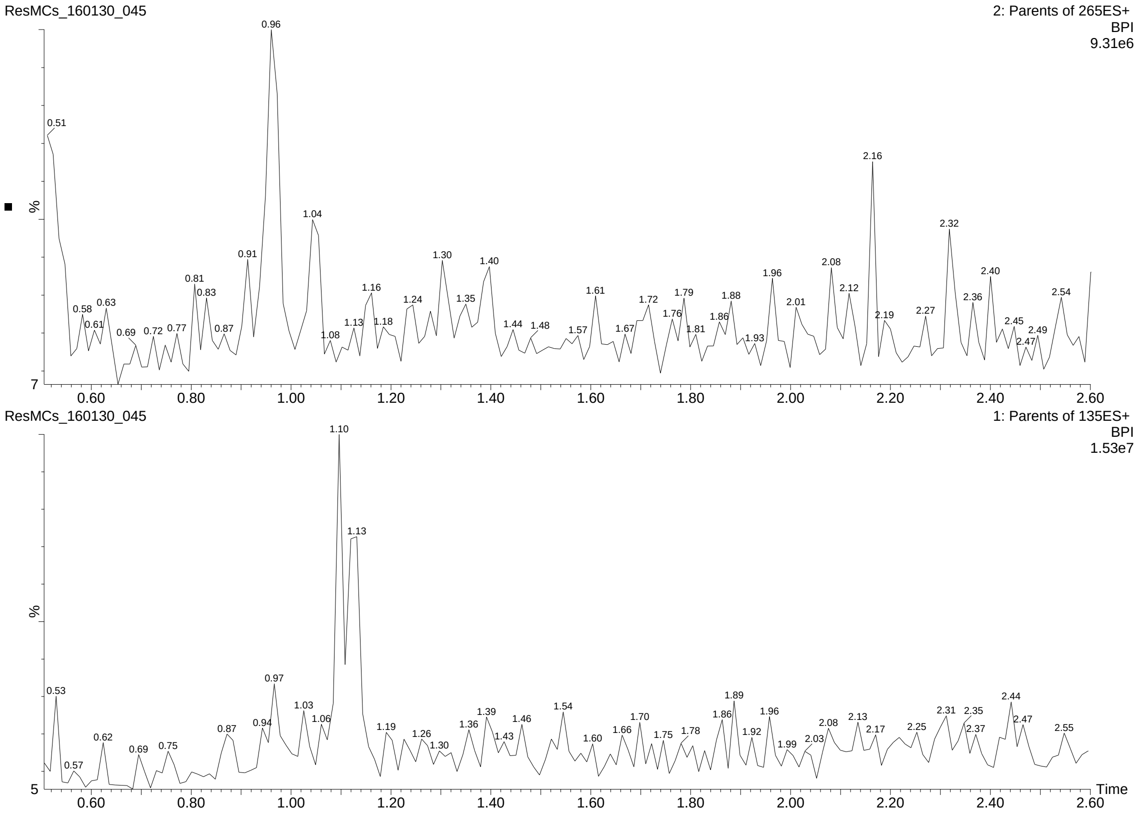

Base-peak chromatogram of the precursor ion scan for SV-54 (CE=40 V).

RT[a] (min)	Prod.[b]	*m/z*[c]	Mr[d] (Da)	Identity	Comments
0.96	265	480.8	-	Unknown	Unlikely to be a microcystin because of retention time in conjunction with *m/z*.
1.04	265	487.8	-	Unknown	Unlikely to be a microcystin because of retention time in conjunction with *m/z*.
1.10	135	837.2	-	Unknown	Unlikely to be a microcystin because of retention time in conjunction with *m/z*.
1.13	135	882.2	-	Unknown	Unlikely to be a microcystin because of retention time in conjunction with *m/z*.
2.16	265	667.7	-	Unknown	Unlikely to be a microcystin because of retention time in conjunction with *m/z*.
2.32	265	586.4	-	Unknown	Unlikely to be a microcystin because of retention time in conjunction with *m/z*.

[a] RT = Retention time on a Waters Acquity BEH-C_{18} UPLC column (2.1×50 mm; 1.7-µm). [b] Product ion trace in which the precursor ion was identified (*m/z* 135 for Adda-containing compounds or *m/z* 265 for ADMAdda-containing compounds). [c] *m/z* = Mass-to-charge ratio of precursor ion. [d] M_r = Molecular weight of the identified precursor ion.

Environmental Sample SV-56

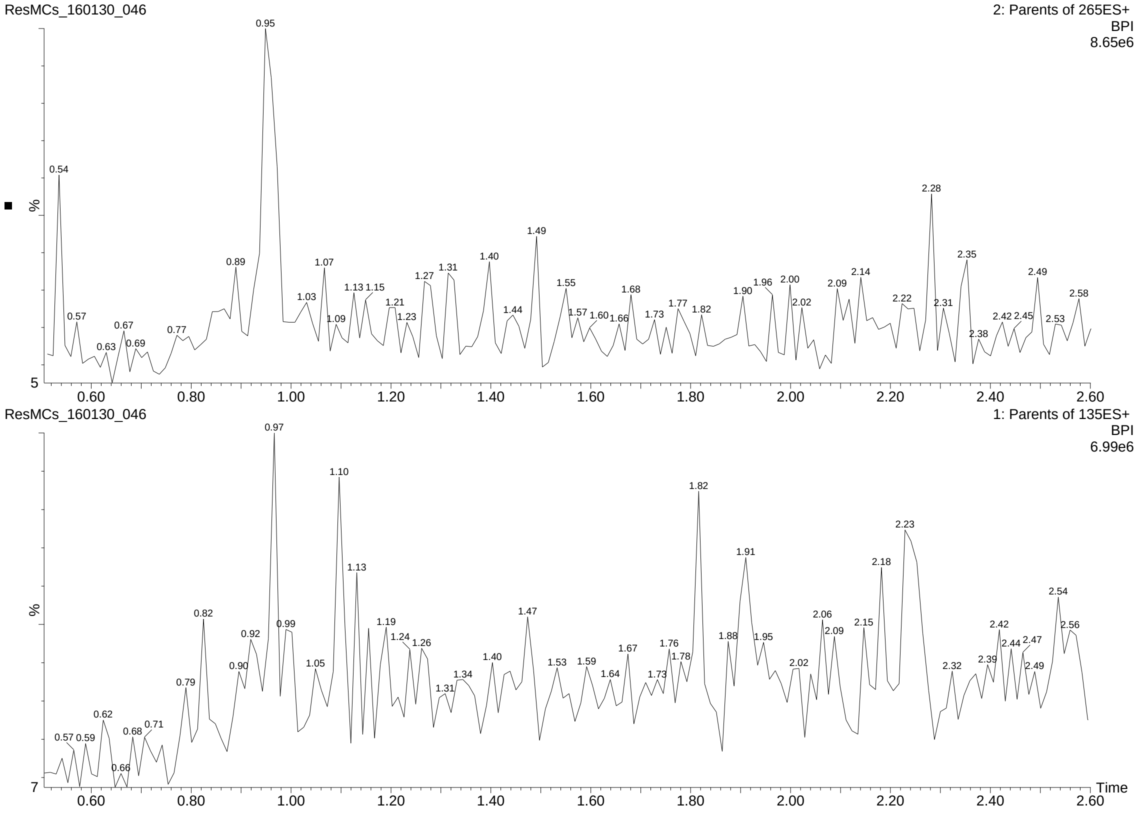

Base-peak chromatogram of the precursor ion scan for SV-56 (CE=40 V).

RT[a] (min)	Prod.[b]	*m/z*[c]	M_r[d] (Da)	Identity	Comments
0.95	265	628.9	-	Unknown	Unlikely to be a microcystin because of retention time in conjunction with *m/z*.
0.97	135	487.9	-	Unknown	Unlikely to be a microcystin because of retention time in conjunction with *m/z*.
1.10	135	837.2	-	Unknown	Unlikely to be a microcystin because of retention time in conjunction with *m/z*.
1.82	135	744.0	-	Unknown	Unlikely to be a microcystin because of retention time in conjunction with *m/z*.
2.23	135	724.2	-	Unknown	Unlikely to be a microcystin because of retention time in conjunction with *m/z*.
2.28	265	543.0	-	Unknown	Unlikely to be a microcystin because of retention time in conjunction with *m/z*.

[a] RT = Retention time on a Waters Acquity BEH-C_{18} UPLC column (2.1×50 mm; 1.7-µm). [b] Product ion trace in which the precursor ion was identified (*m/z* 135 for Adda-containing compounds or *m/z* 265 for ADMAdda-containing compounds). [c] *m/z* = Mass-to-charge ratio of precursor ion. [d] M_r = Molecular weight of the identified precursor ion.

Environmental Sample SV-74

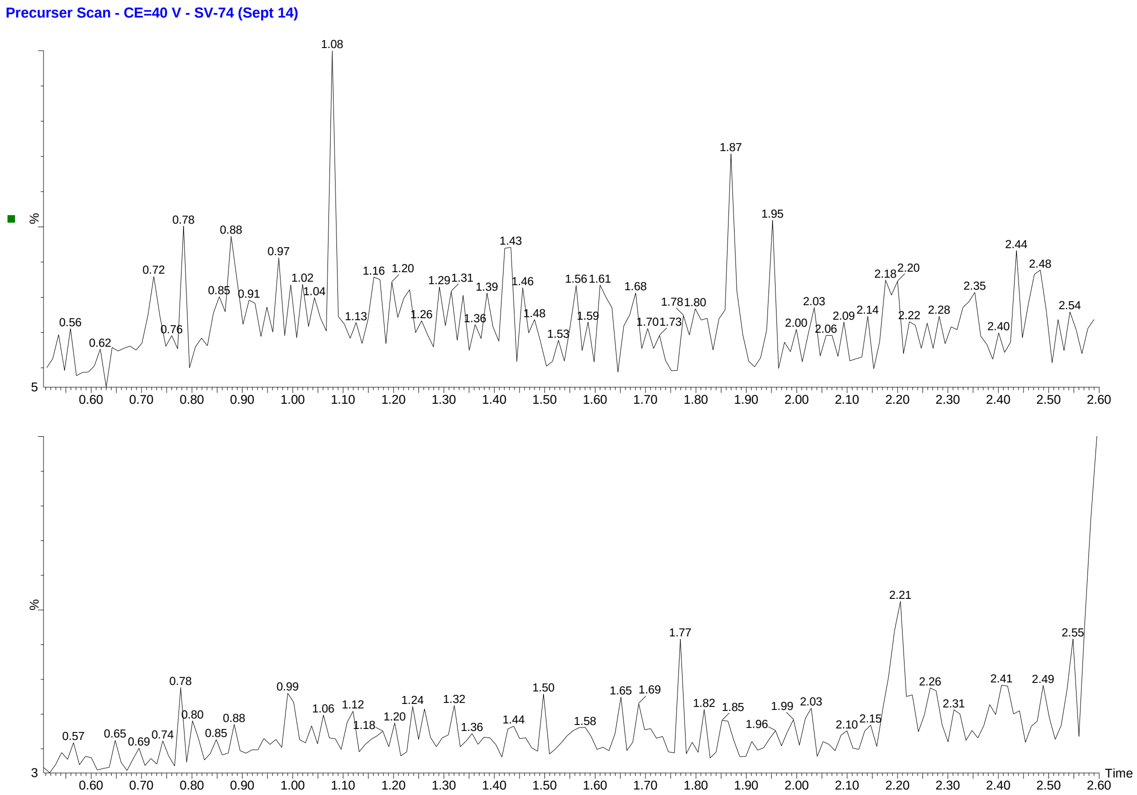

Base-peak chromatogram of the precursor ion scan for SV-74 (CE=40 V).

RT[a] (min)	Prod.[b]	*m*/*z*[c]	M_r[d] (Da)	Identity	Comments
0.82	265	598.8	-	Unknown	Unlikely to be a microcystin because of retention time in conjunction with *m*/*z*.
0.99	135	474.0	-	Unknown	Unlikely to be a microcystin because of retention time in conjunction with *m*/*z*.
1.27	135	596.4	-	Unknown	MS/MS demonstrated this was not a MC.
1.92	135	910.6	909.5	MC-LA	MS/MS used to confirm identity. Thiol derivatisation confirmed presence of Mdha.
2.20	135	968.3	-	Unknown	MS/MS demonstrated this was not a MC.
2.42	135	948.2	-	Unknown	Unlikely to be a microcystin because of retention time in conjunction with *m*/*z*.

[a] RT = Retention time on a Waters Acquity BEH-C_{18} UPLC column (2.1×50 mm; 1.7-µm). [b] Product ion trace in which the precursor ion was identified (*m*/*z* 135 for Adda-containing compounds or *m*/*z* 265 for ADMAdda-containing compounds). [c] *m*/*z* = Mass-to-charge ratio of precursor ion. [d] M_r = Molecular weight of the identified precursor ion.

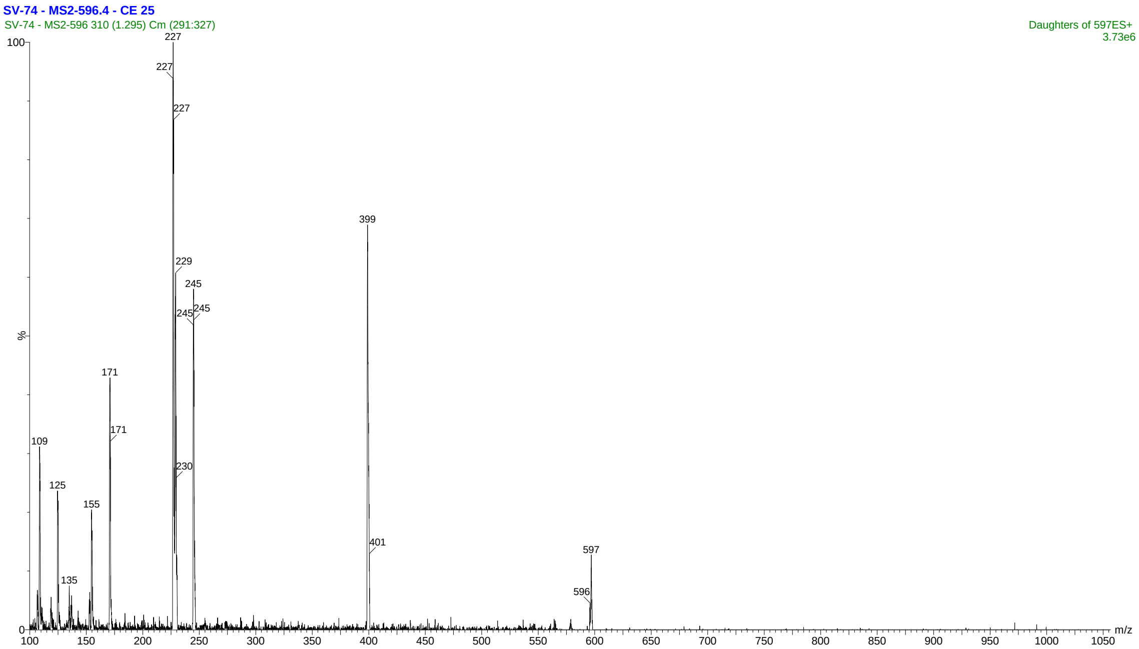

MS/MS spectrum of the 596.4 ion at 1.29 min from SV-74 (Sept 14; CE=25-30 V).

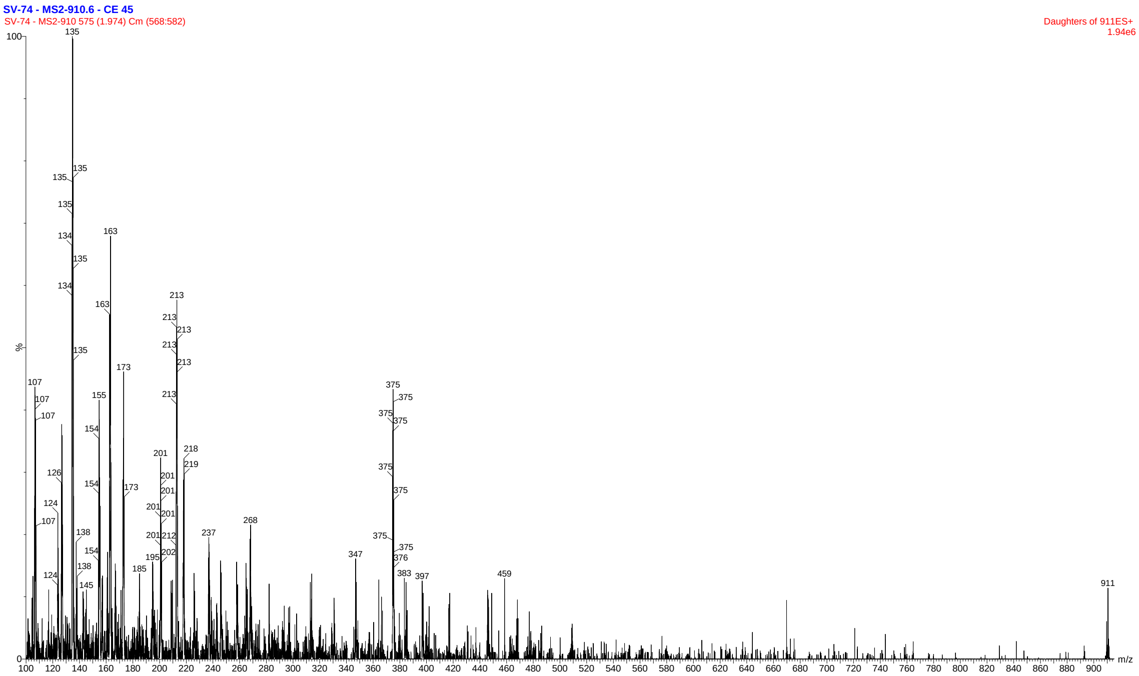

MS/MS spectrum of the 910.6 [M+H]$^+$ ion at 1.97 min from SV-74 (CE=35-45 V).

Environmental Sample SV-75

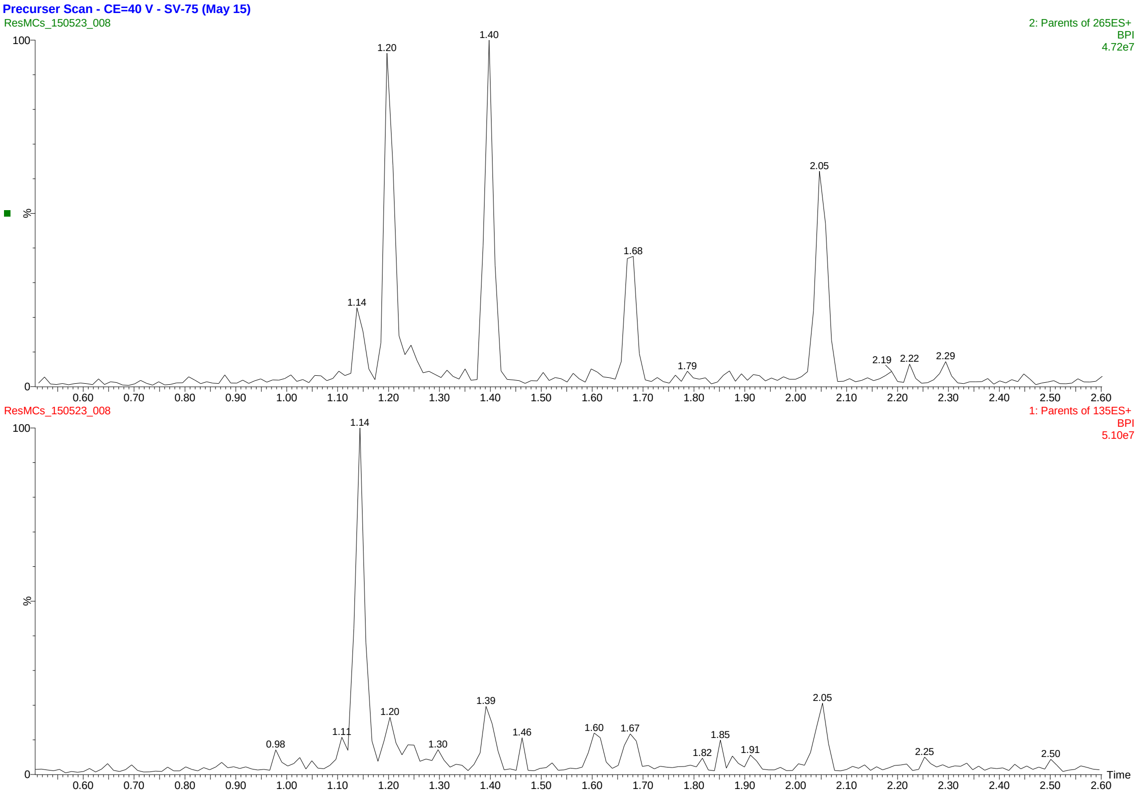

Base-peak chromatogram of the precursor ion scan for SV-75 (CE=40 V).

RT[a] (min)	Prod.[b]	*m/z*[c]	M_r[d] (Da)	Identity	Comments
1.14	265	526.7	1051.6	[Asp[3], ADMAdda[5], Dhb[7]] MC-RR	MS/MS used to confirm identity. Thiol derivatisation confirmed presence of Dhb.
1.20	265	978.5	-	Unknown	MS/MS indicated it is not a microcystin.
1.40	265	1009.6	1008.5	[Asp[3], ADMAdda[5], Dhb[7]] MC-LR	MS/MS used to confirm identity. Thiol derivatisation confirmed presence of Dhb.
1.60	135	1083.9	-	Unknown	MS/MS indicated it is not a microcystin.
1.67	135	1084.4	-	Unknown	MS/MS indicated it is not a microcystin.
1.68	265	1084.4	-	Unknown	MS/MS indicated it is not a microcystin.
2.05	265	1098.7	-	Unknown	MS/MS indicated it is not a microcystin.
2.05	135	1098.7	-	Unknown	MS/MS indicated it is not a microcystin.

[a] RT = Retention time on a Waters Acquity BEH-C_{18} UPLC column (2.1×50 mm; 1.7-µm). [b] Product ion trace in which the precursor ion was identified (*m/z* 135 for Adda-containing compounds or *m/z* 265 for ADMAdda-containing compounds). [c] *m/z* = Mass-to-charge ratio of precursor ion. [d] M_r = Molecular weight of the identified precursor ion.

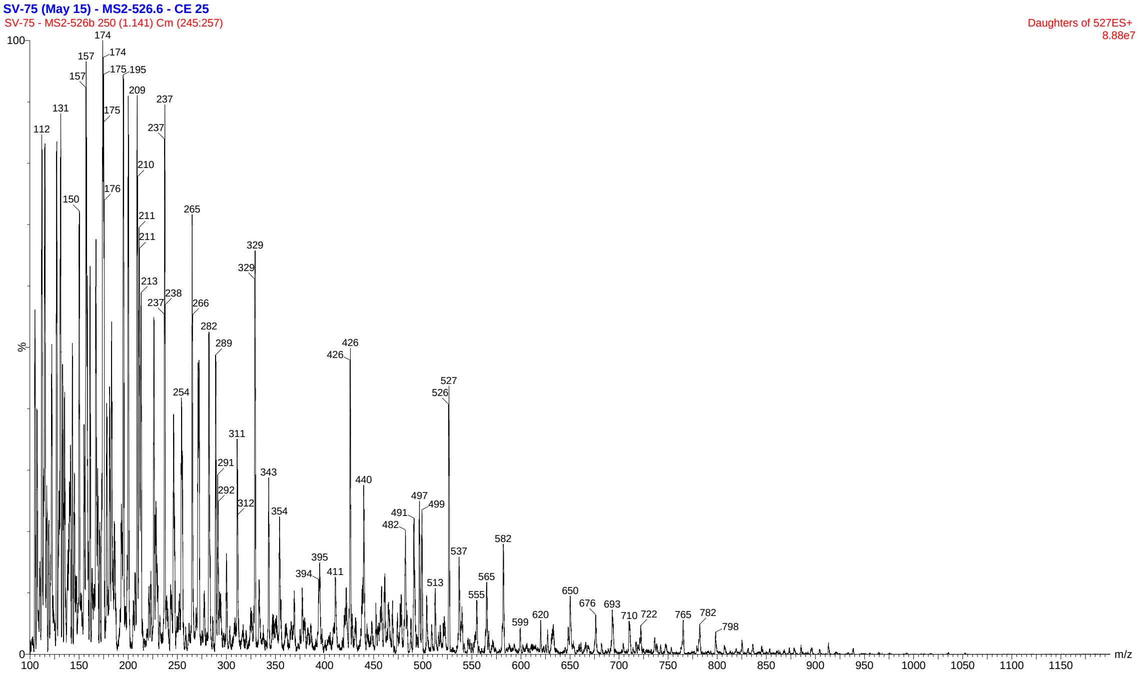

MS/MS spectrum of the 526.7 [M+2H]$^{2+}$ ion at 1.14 min from SV-75 (CE=25-30 V).

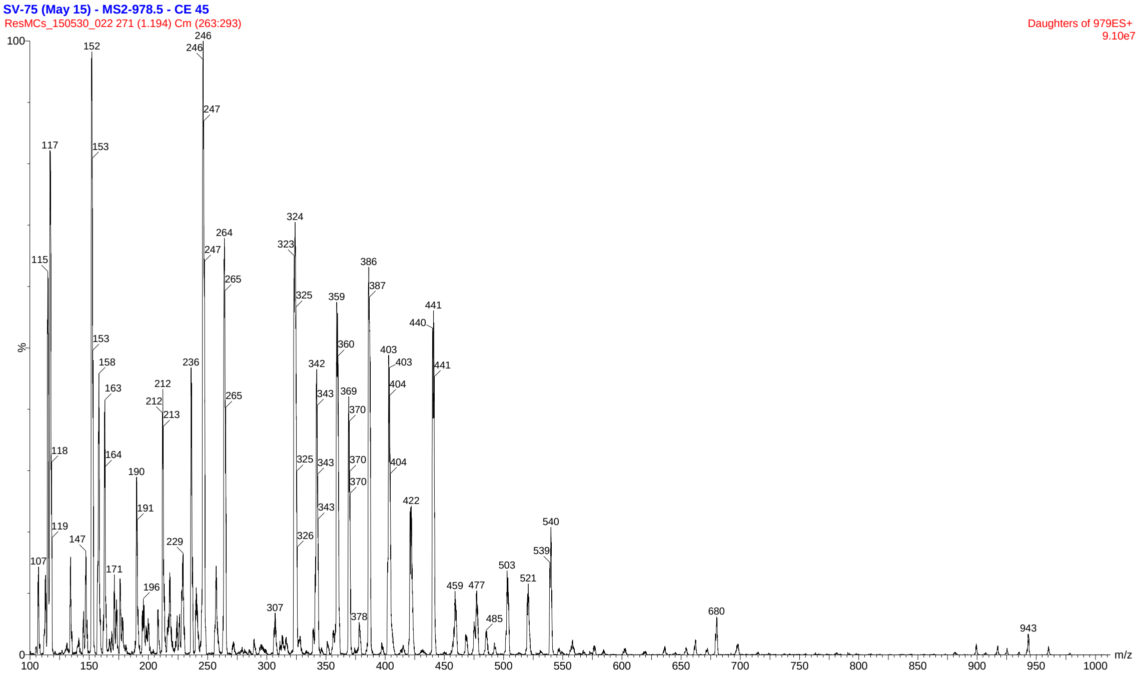

MS/MS spectrum of the 978.5 ion at 1.20 min from SV-75 (CE=45 V).

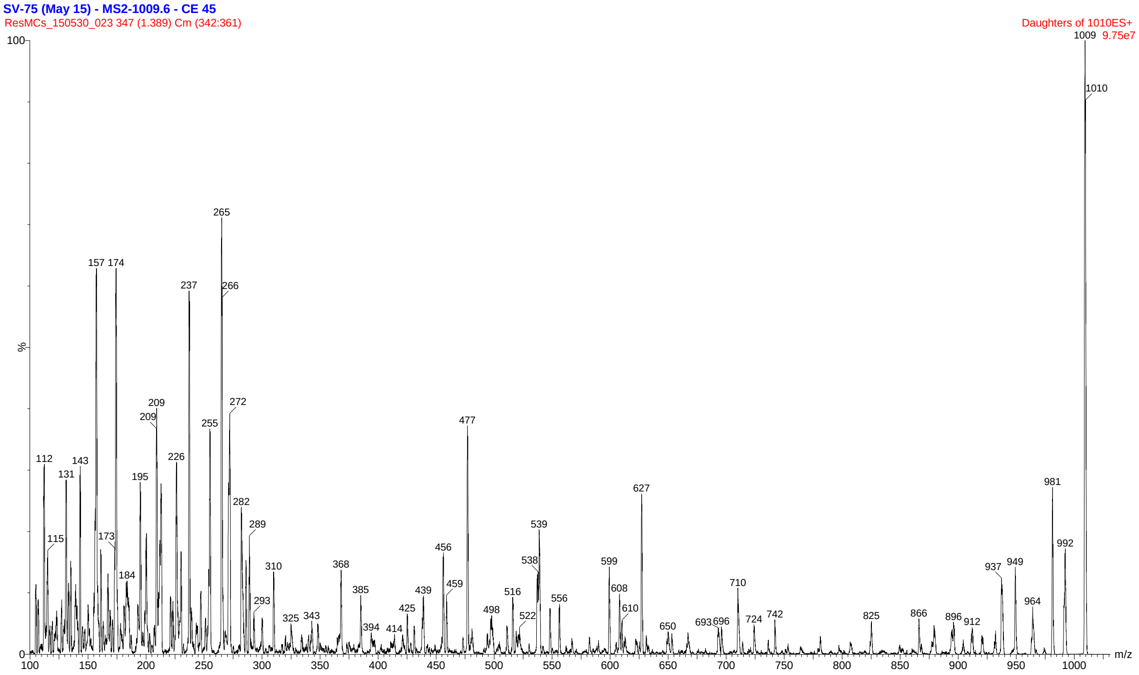

MS/MS spectrum of the 1009.6 [M+H]+ ion at 1.40 min from SV-75 (CE=45 V).

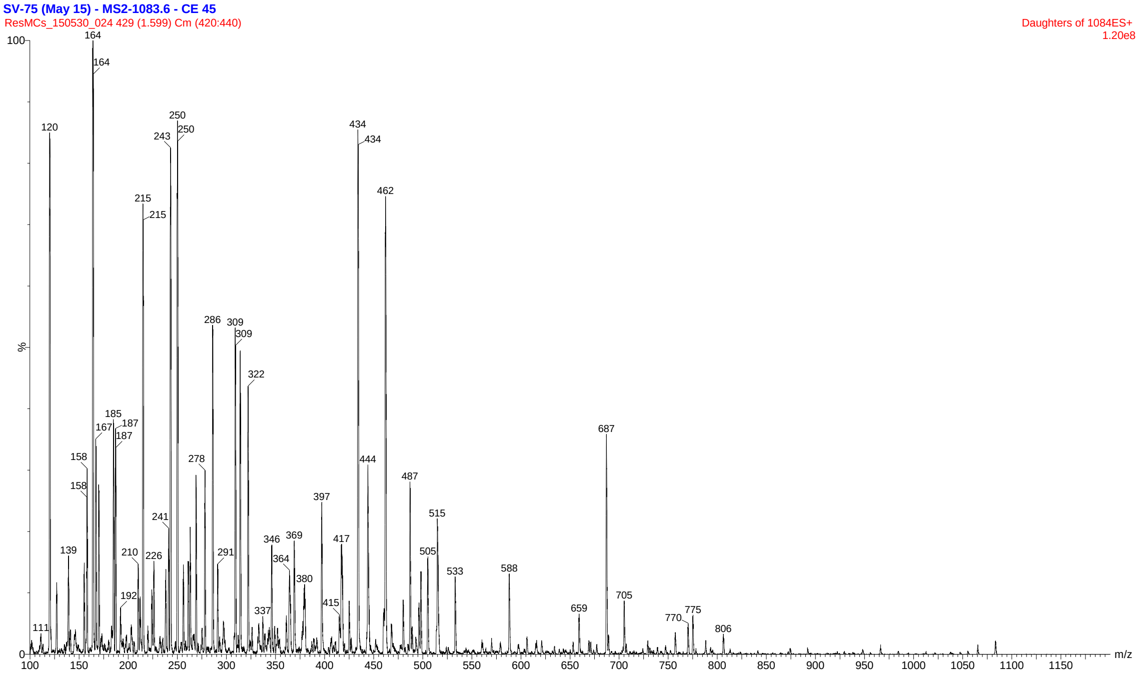

MS/MS spectrum of the 1083.9 ion at 1.60 min from SV-75 (CE=45 V).

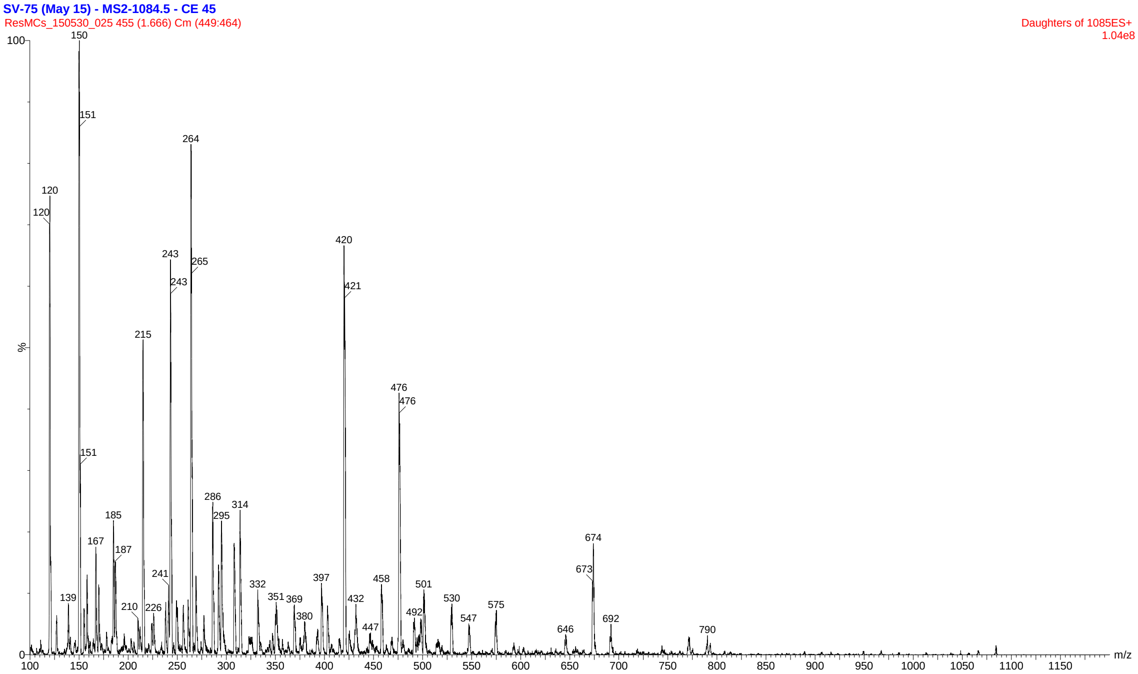

MS/MS spectrum of the 1084.4 ion at 1.68 min from SV-75 (CE=45 V).

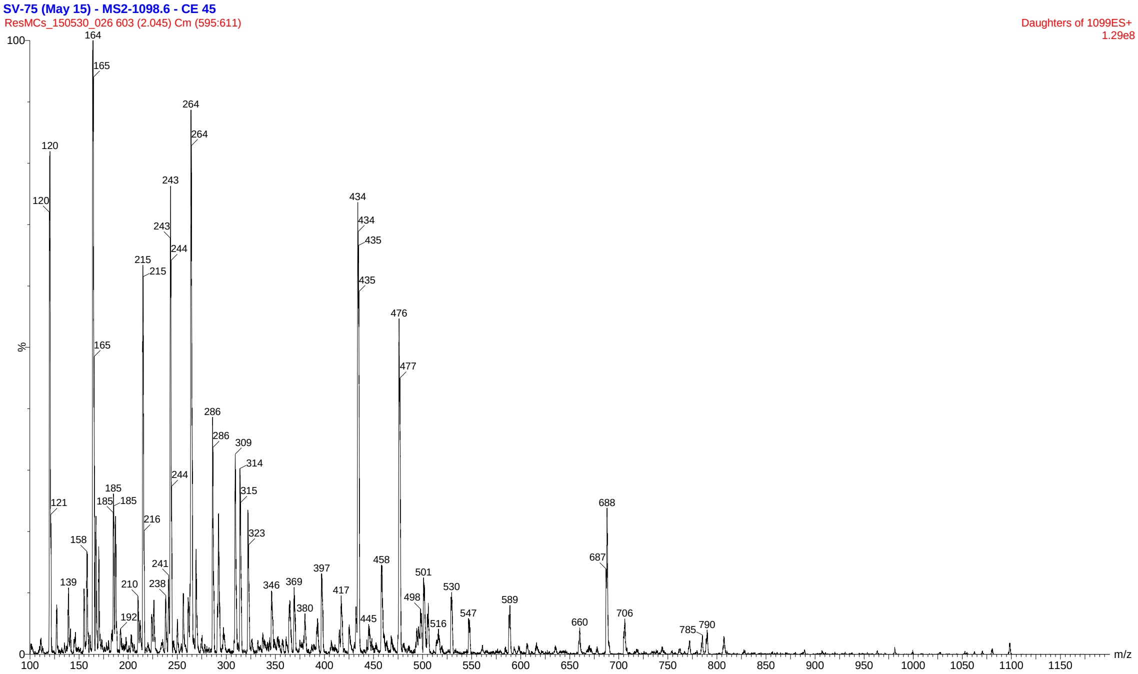

MS/MS spectrum of the 1098.7 ion at 2.05 min from SV-75 (CE=45 V).

Environmental Sample SV-80

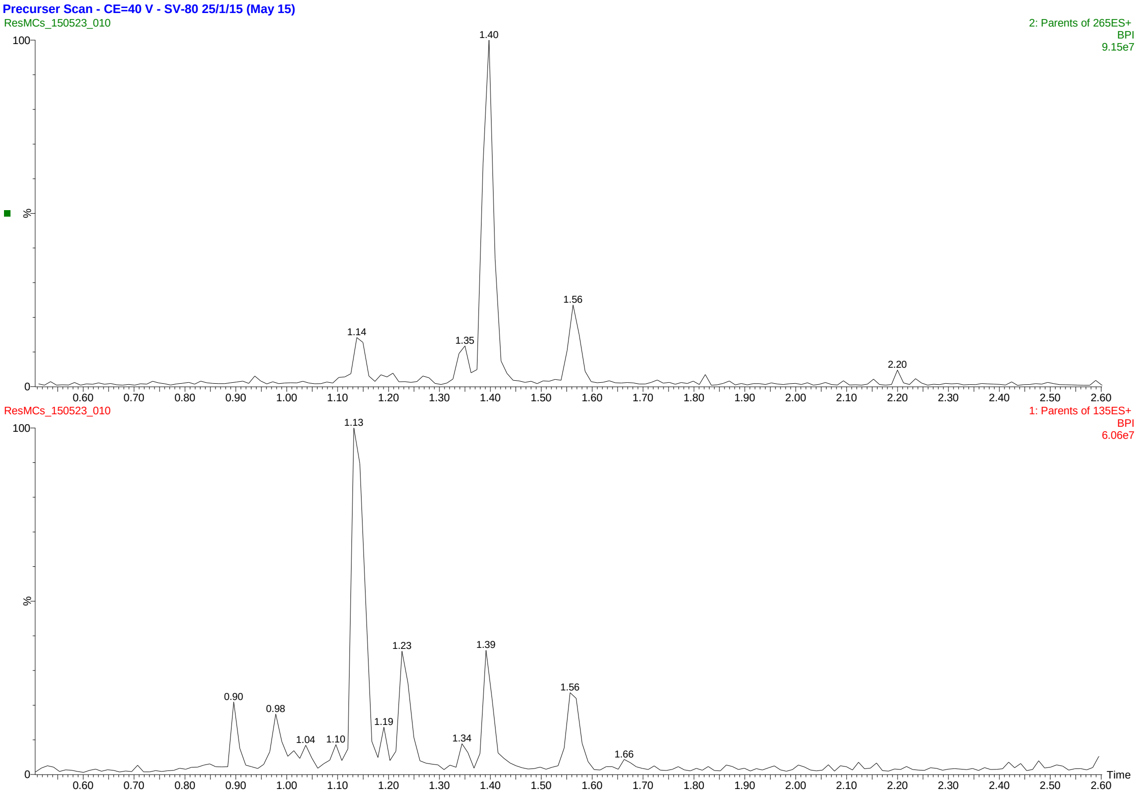

Base-peak chromatogram of the precursor ion scan for SV-80 (CE=40 V).

RT[a] (min)	Prod.[b]	*m*/*z*[c]	M_r[d] (Da)	Identity	Comments
0.90	135	514.6	1027.5	[Asp^3, Ser^7] MC-RR	MS/MS confirmed the identity of the MC.
0.98	135	505.4	1009.5	[Asp^3, Dha^7] MC-RR	MS/MS used to confirm identity. Thiol derivatisation confirmed presence of Dha.
1.13	135/265	526.6/ 1052.5	1051.6	[Asp^3, ADMAdda^5, Dhb^7] MC-RR	MS/MS used to confirm identity. Thiol derivatisation confirmed presence of Dhb.
1.23	135	550.2	-	Unknown	Tandem MS indicated that it was not a MC.
1.35	265	1027.7	1026.5	[Asp^3, ADMAdda^5, Thr^7] MC-LR	MS/MS confirmed the identity of the MC.
1.39	135/265	505.4/ 1009.6	1008.5	[Asp^3, ADMAdda^5, Dhb^7] MC-LR	MS/MS used to confirm identity. Thiol derivatisation confirmed presence of Dhb.
1.56	135/265	1114.6	-	Unknown	Tandem MS indicated that it was not a MC.

[a] RT = Retention time on a Waters Acquity BEH-C_{18} UPLC column (2.1×50 mm; 1.7-µm). [b] Product ion trace in which the precursor ion was identified (*m*/*z* 135 for Adda-containing compounds or *m*/*z* 265 for ADMAdda-containing compounds). [c] *m*/*z* = Mass-to-charge ratio of precursor ion. [d] M_r = Molecular weight of the identified precursor ion.

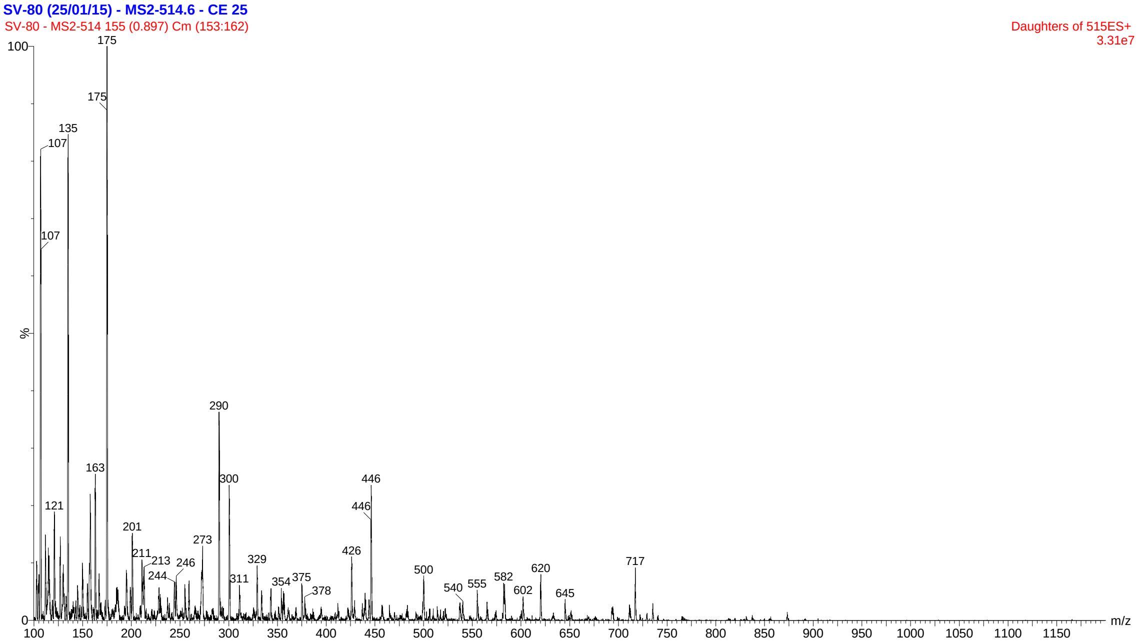

MS/MS spectrum of the 514.6 $[M+2H]^{2+}$ ion at 0.90 min from SV-80 (CE=25-30 V).

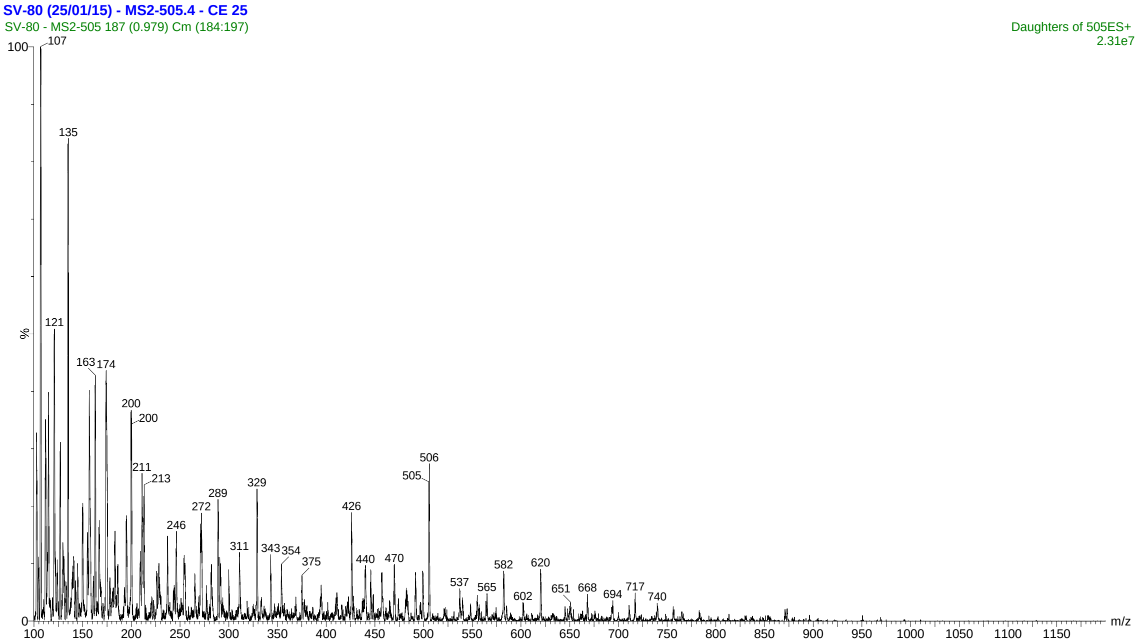

MS/MS spectrum of the *m/z* 505.4 $[M+2H]^{2+}$ ion at 0.98 min from SV-80 (CE=25-30 V).

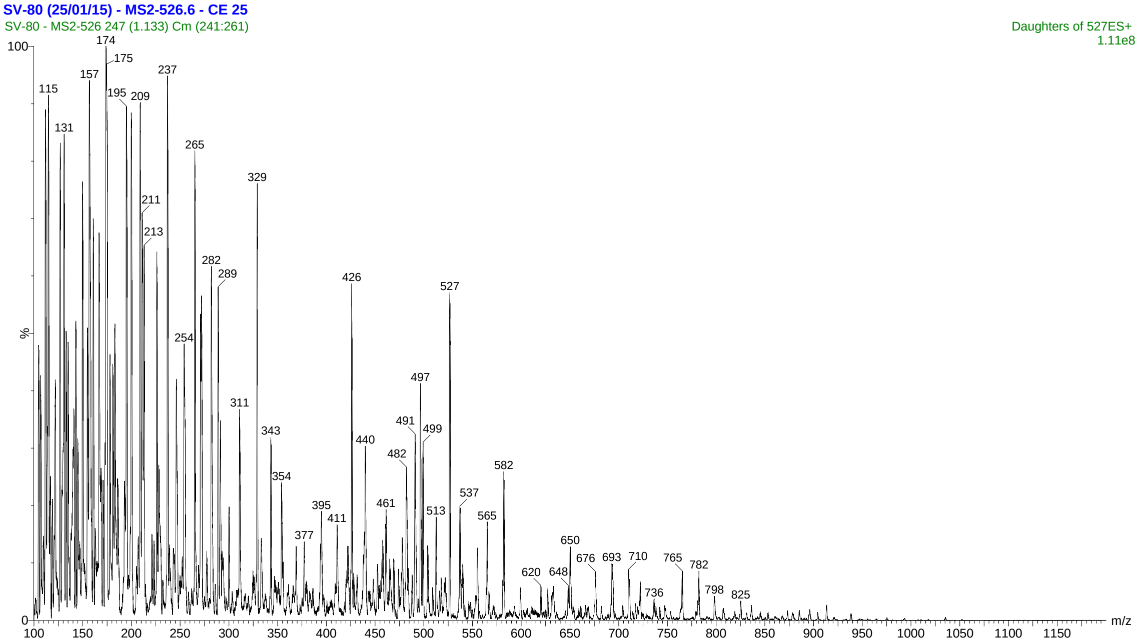

MS/MS spectrum of the *m/z* 526.6 [M+2H]$^{2+}$ ion at 1.13 min from SV-80 (CE=25-30 V).

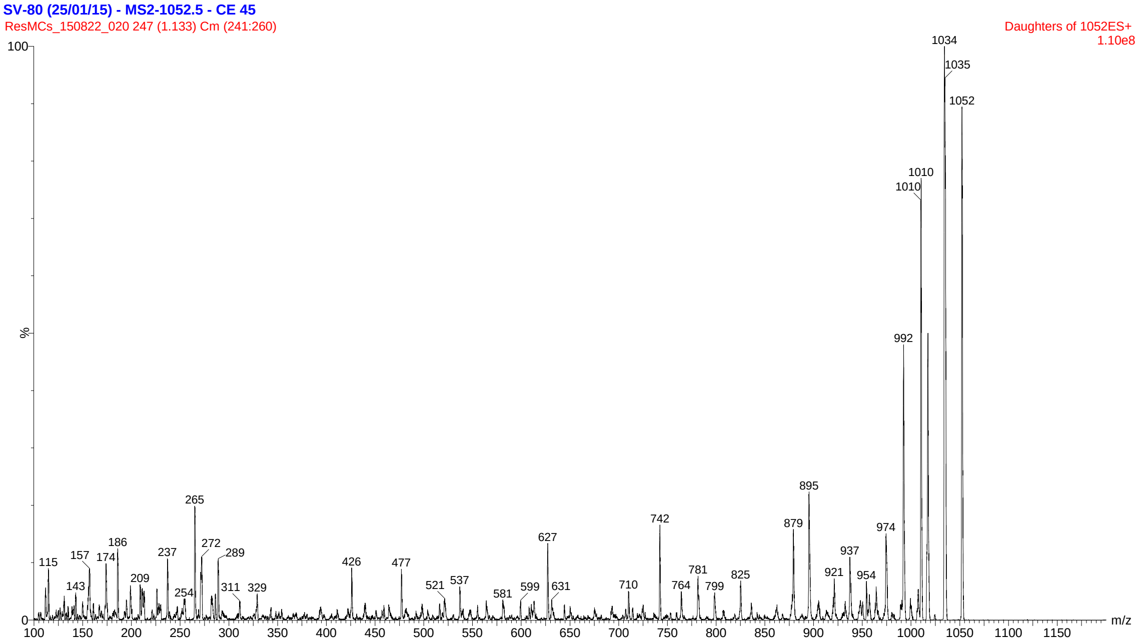

MS/MS spectrum of the *m/z* 1052.5 [M+H]$^{+}$ ion at 1.13 min from SV-80 (CE=45 V).

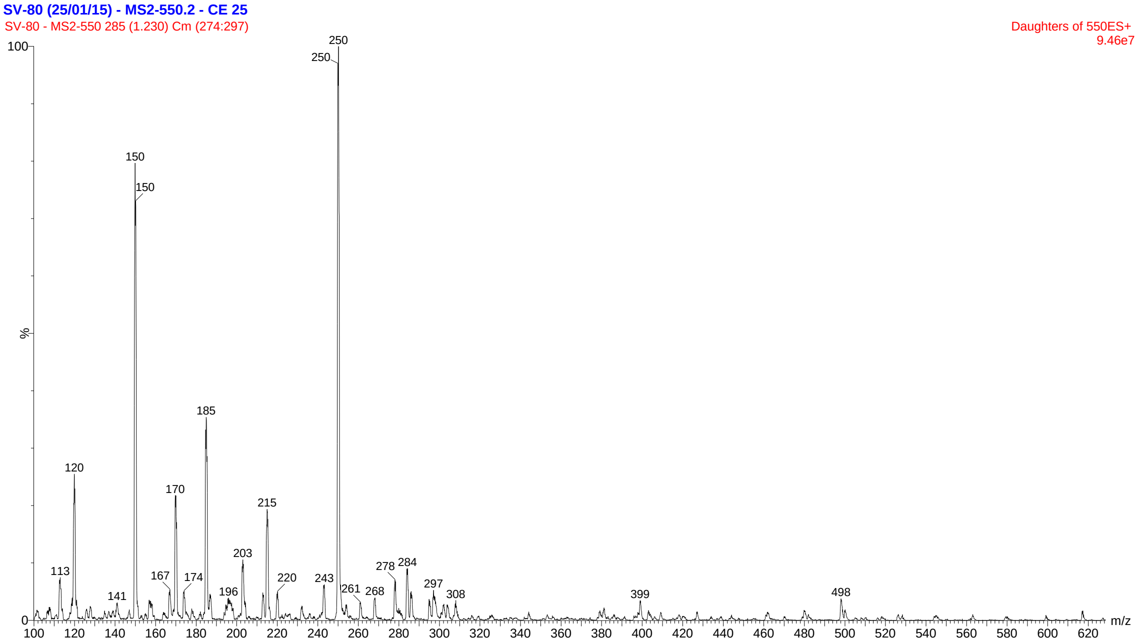

MS/MS spectrum of the *m/z* 550.2 ion at 1.23 min from SV-80 (CE=25-30 V).

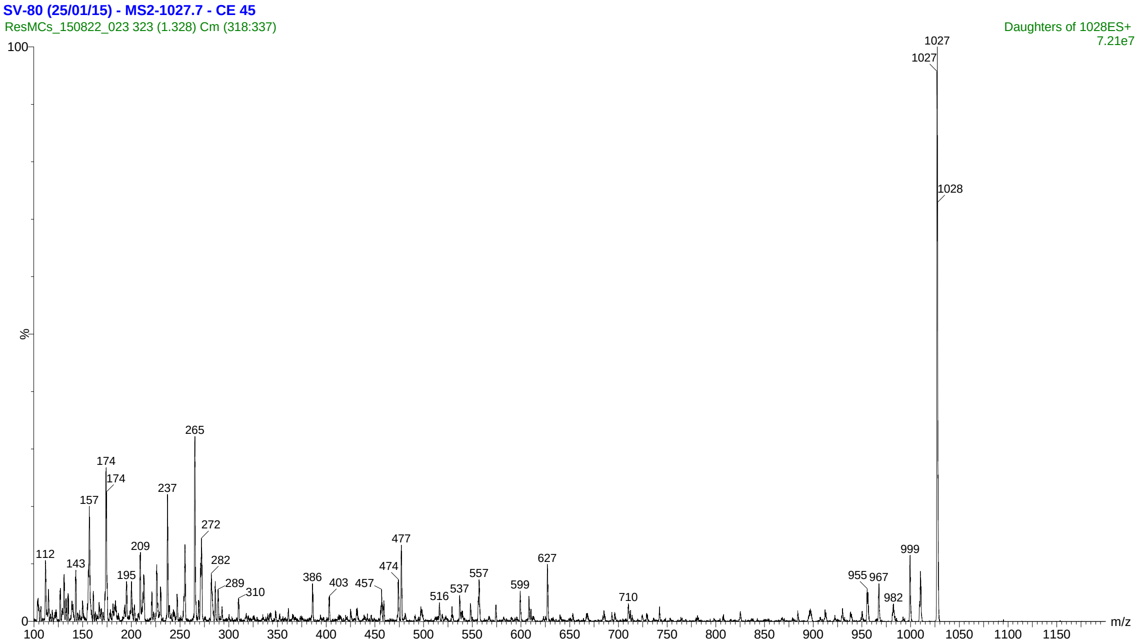

MS/MS spectrum of the *m/z* 1027.7 $[M+H]^+$ ion at 1.35 min from SV-80 (CE=45 V).

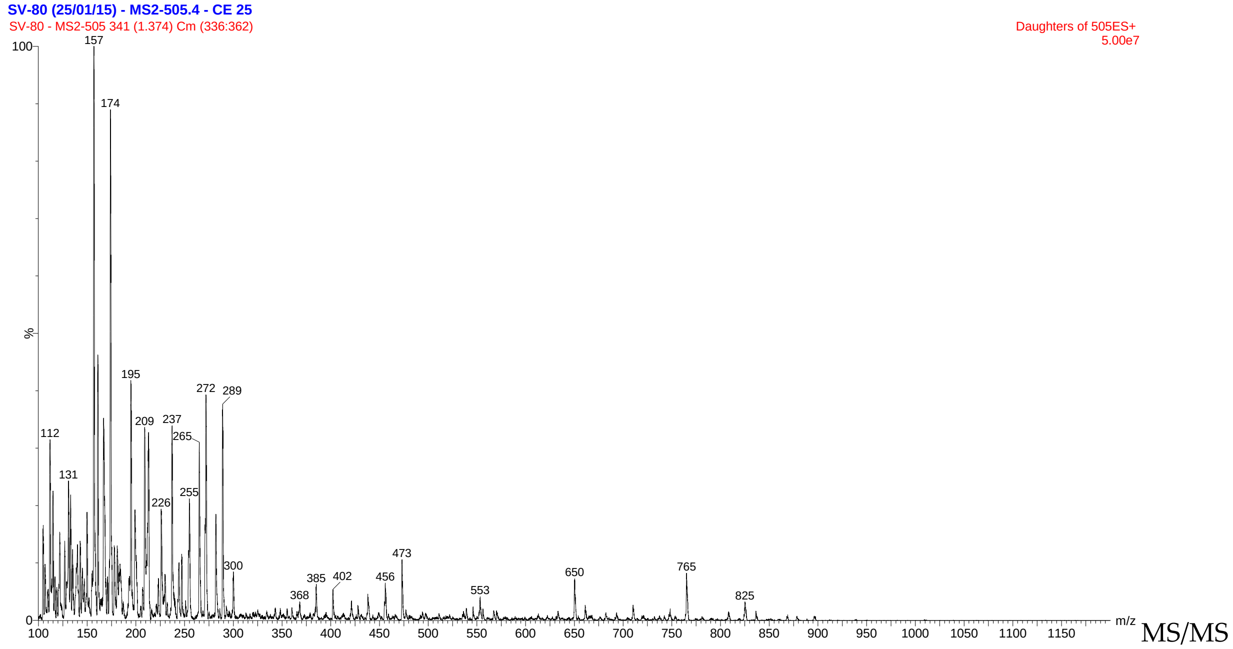

MS/MS spectrum of the *m/z* 505.4 $[M+2H]^{2+}$ ion at 1.39 min from SV-80 (CE=25-30 V).

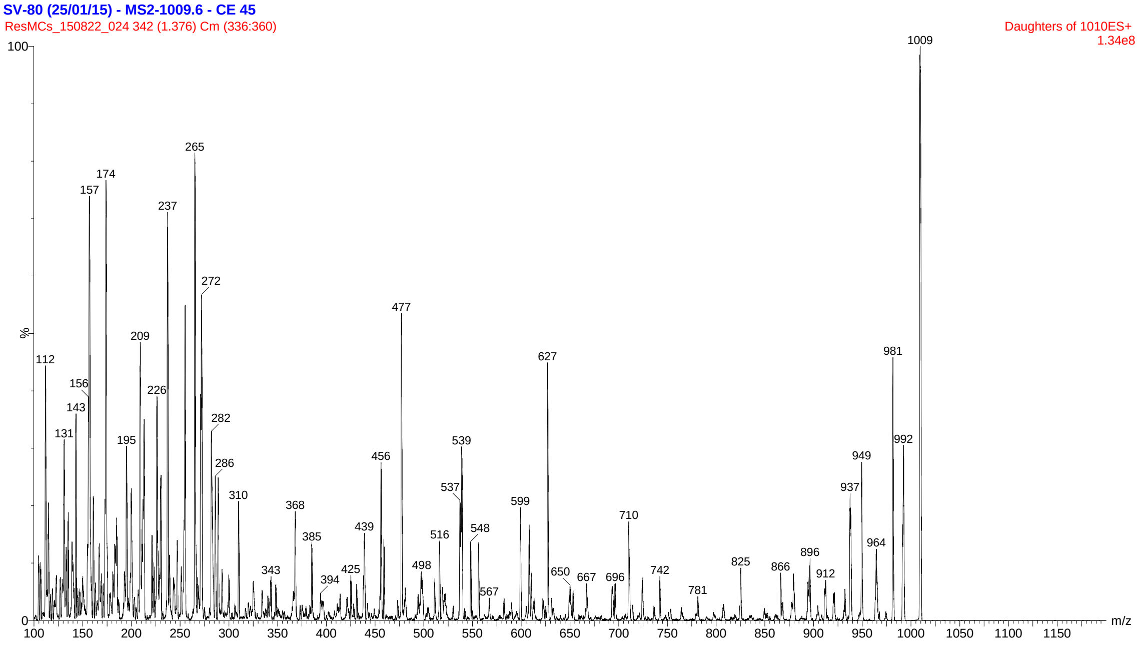

MS/MS spectrum of the *m/z* 1009.6 $[M+H]^+$ ion at 1.39 min from SV-80 (CE=45 V).

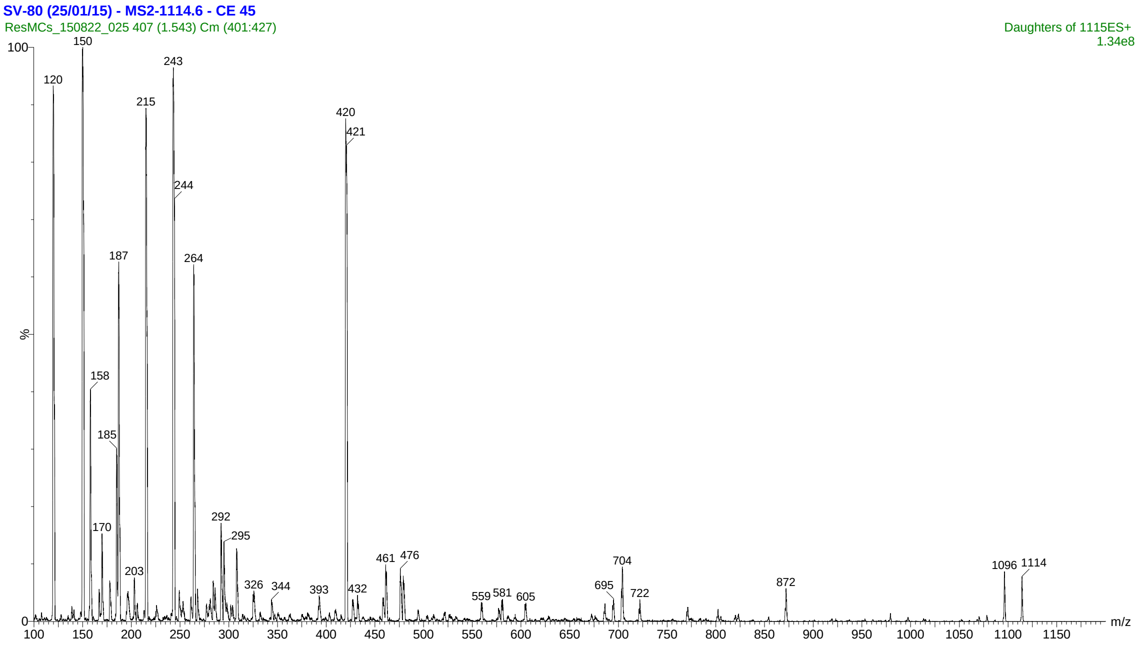

MS/MS spectrum of the *m*/*z* 1114.6 ion at 1.56 min from SV-80 (CE=45 V).

Environmental Sample SV-81

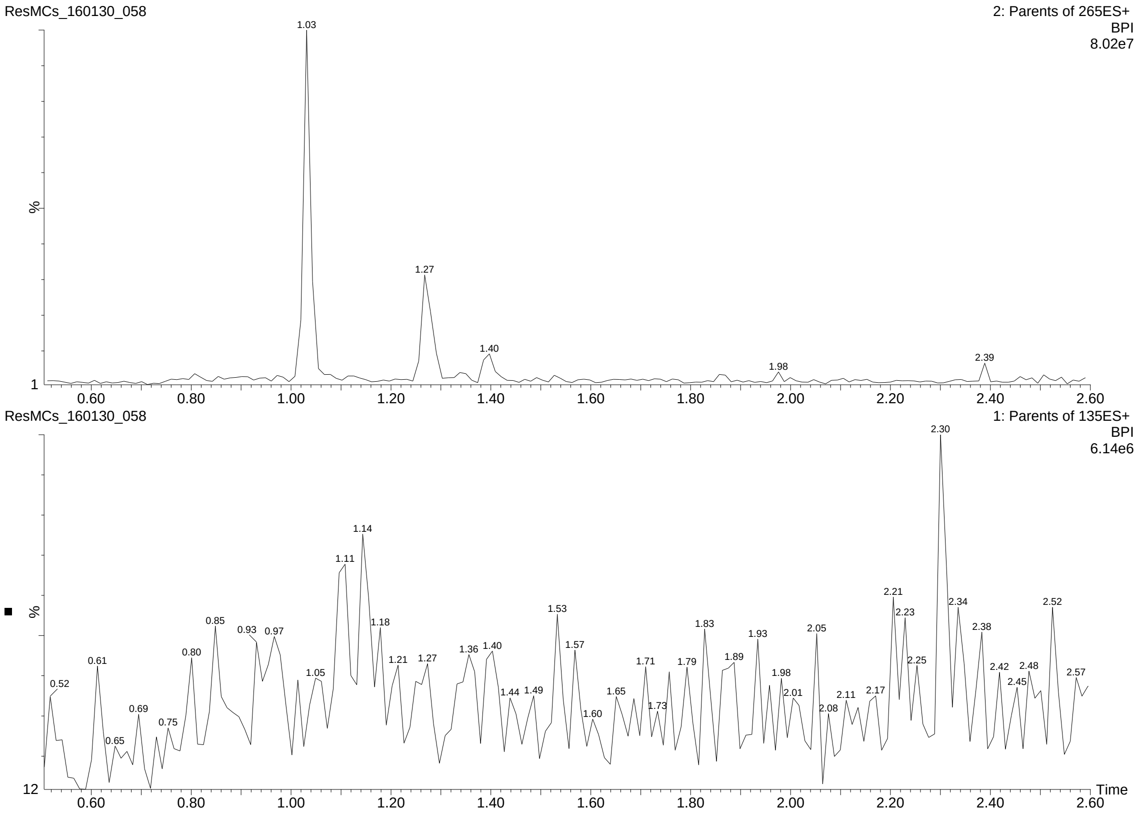

Base-peak chromatogram of the precursor ion scan for SV-81 (CE=40 V).

RT[a] (min)	Prod.[b]	*m*/*z*[c]	M_r[d] (Da)	Identity	Comments
1.03	265	733.2	-	Unknown	Unlikely to be a microcystin because of retention time in conjunction with *m*/*z*.
1.27	265	801.3	-	Unknown	Unlikely to be a microcystin because of retention time in conjunction with *m*/*z*.
1.40	265	1009.7	1008.5	[Asp[3], ADMAdda[5], Dhb[7]] MC-LR	MS/MS used to confirm identity. Thiol derivatisation confirmed presence of Dhb.
1.98	265	674.3	-	Unknown	Unlikely to be a microcystin because of retention time in conjunction with *m*/*z*.
2.30	135	819.6	-	Unknown	Unlikely to be a microcystin because of retention time in conjunction with *m*/*z*.
2.39	265	551.9	-	Unknown	Unlikely to be a microcystin because of retention time in conjunction with *m*/*z*.

[a] RT = Retention time on a Waters Acquity BEH-C_{18} UPLC column (2.1×50 mm; 1.7-µm). [b] Product ion trace in which the precursor ion was identified (*m*/*z* 135 for Adda-containing compounds or *m*/*z* 265 for ADMAdda-containing compounds). [c] *m*/*z* = Mass-to-charge ratio of precursor ion. [d] M_r = Molecular weight of the identified precursor ion.

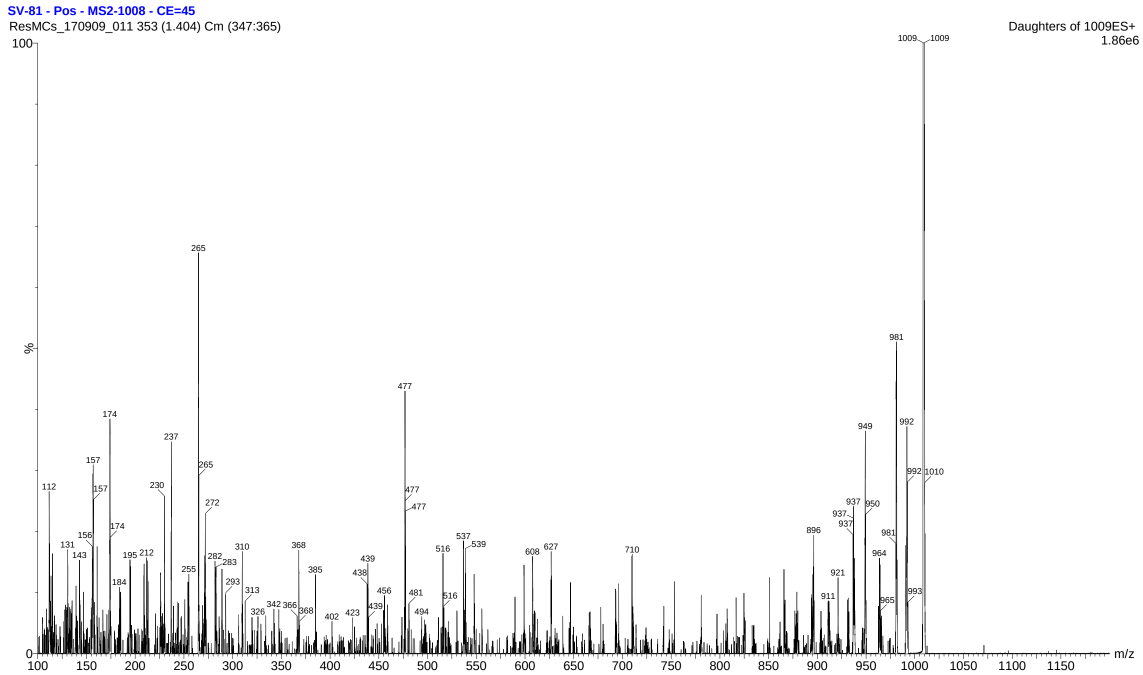

MS/MS spectrum of the *m*/*z* 1009.7 [M+H] + ion at 1.40 min from SV-81 (CE=45 V).

Environmental Sample SV-83

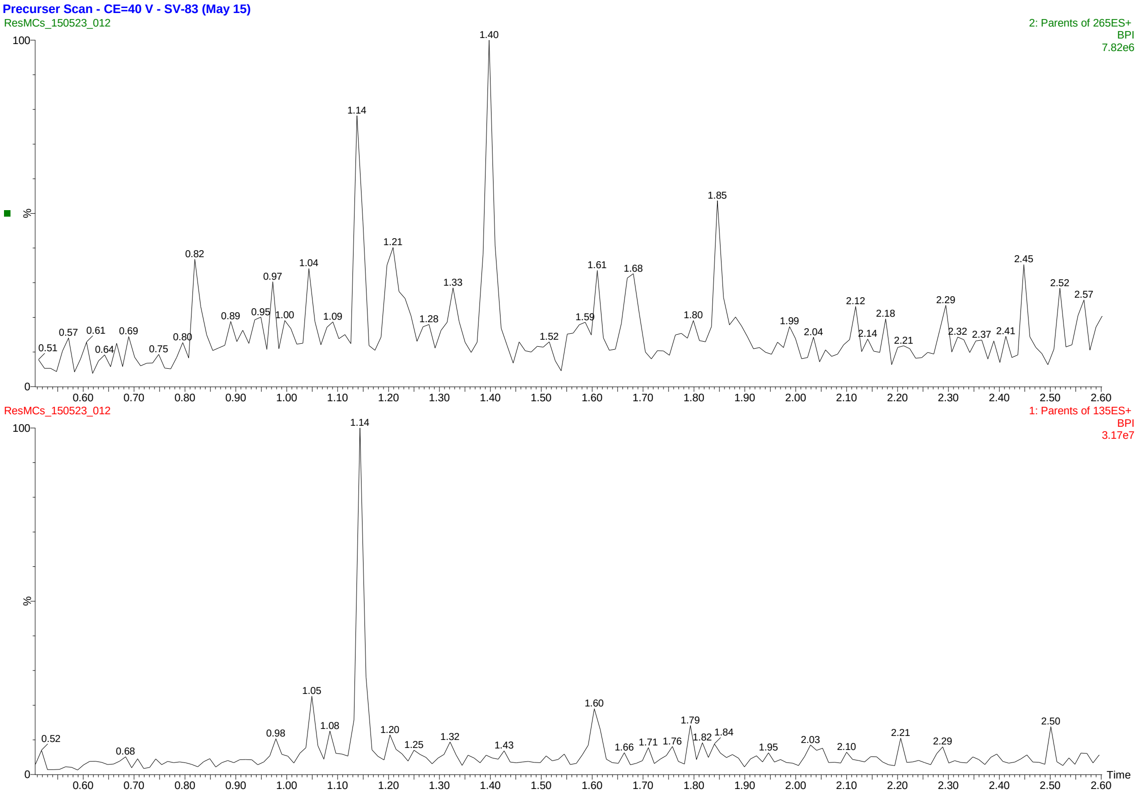

Base-peak chromatogram of the precursor ion scan for SV-83 (CE=40 V).

RT[a] (min)	Prod.[b]	*m*/*z*[c]	M_r[d] (Da)	Identity	Comments
0.82	265	589.3	-	Unknown	Unlikely to be a microcystin because of retention time in conjunction with *m*/*z*.
1.04	265	823.1	-	Unknown	Unlikely to be a microcystin because of retention time in conjunction with *m*/*z*.
1.05	135	825.1	-	Unknown	Unlikely to be a microcystin because of retention time in conjunction with *m*/*z*.
1.14	135/265	526.6	1051.5	[Asp^3, $ADMAdda^5$, Dhb^7] MC-RR	MS/MS used to confirm identity. Thiol derivatisation confirmed presence of Dhb.
1.20	265	978.5	-	Unknown	Unlikely to be a microcystin because of retention time in conjunction with *m*/*z*.
1.21	265	1053.3	1052	Unknown microcystin	MS/MS indicates it is a microcystin. Thiol derivatisation confirmed presence of Dhb.
1.33	265	995.3	-	Unknown	Insufficient signal in MS/MS spectrum.
1.40	265	1009.8	1008.5	[Asp^3, $ADMAdda^5$, Dhb^7] MC-LR	MS/MS used to confirm identity. Thiol derivatisation confirmed presence of Dhb.
1.60	135	809.3	-	Unknown	Unlikely to be a microcystin because of retention time in conjunction with *m*/*z*.
1.61	265	1043.5	-	Unknown	Tandem MS indicates it is not a microcystin.
1.61	265	1025.2	-	Unknown	Tandem MS indicates it is not a microcystin.
1.68	265	1084.6	-	Unknown	Tandem MS indicates it is not a microcystin.
1.85	265	814.9	-	Unknown	Unlikely to be a microcystin because of retention time in conjunction with *m*/*z*.

[a] RT = Retention time on a Waters Acquity BEH-C_{18} UPLC column (2.1×50 mm; 1.7-µm). [b] Product ion trace in which the precursor ion was identified (*m*/*z* 135 for Adda-containing compounds or *m*/*z* 265 for ADMAdda-containing compounds). [c] *m*/*z* = Mass-to-charge ratio of precursor ion. [d] M_r = Molecular weight of the identified precursor ion.

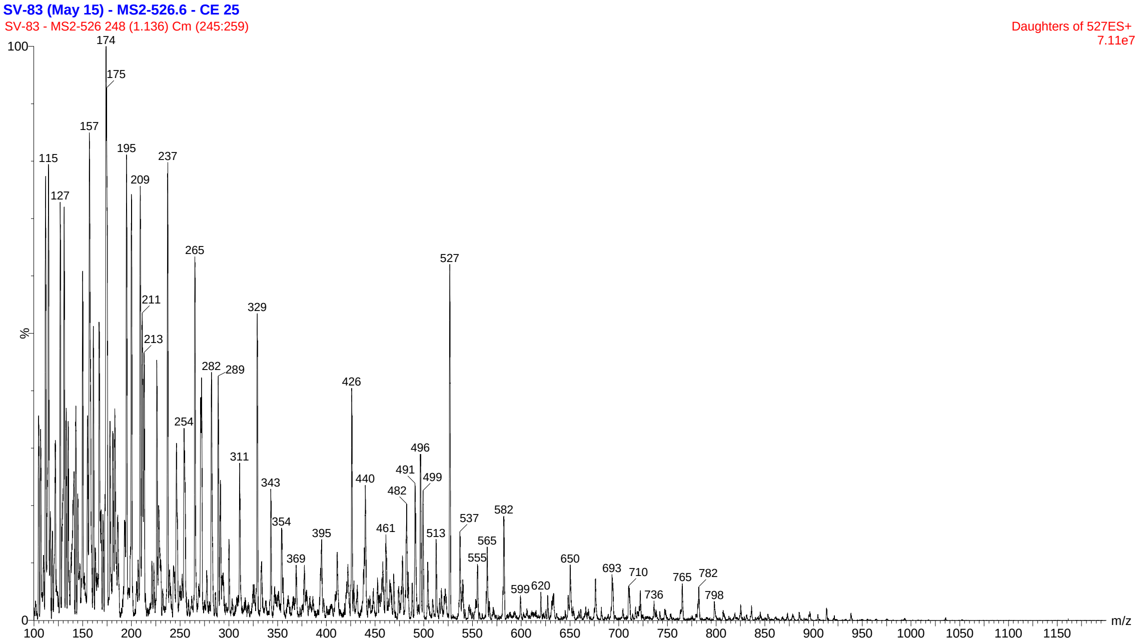

MS/MS spectrum of the *m/z* 526.4 $[M+2H]^{2+}$ ion at 1.14 min from SV-83 (CE=25-30 V).

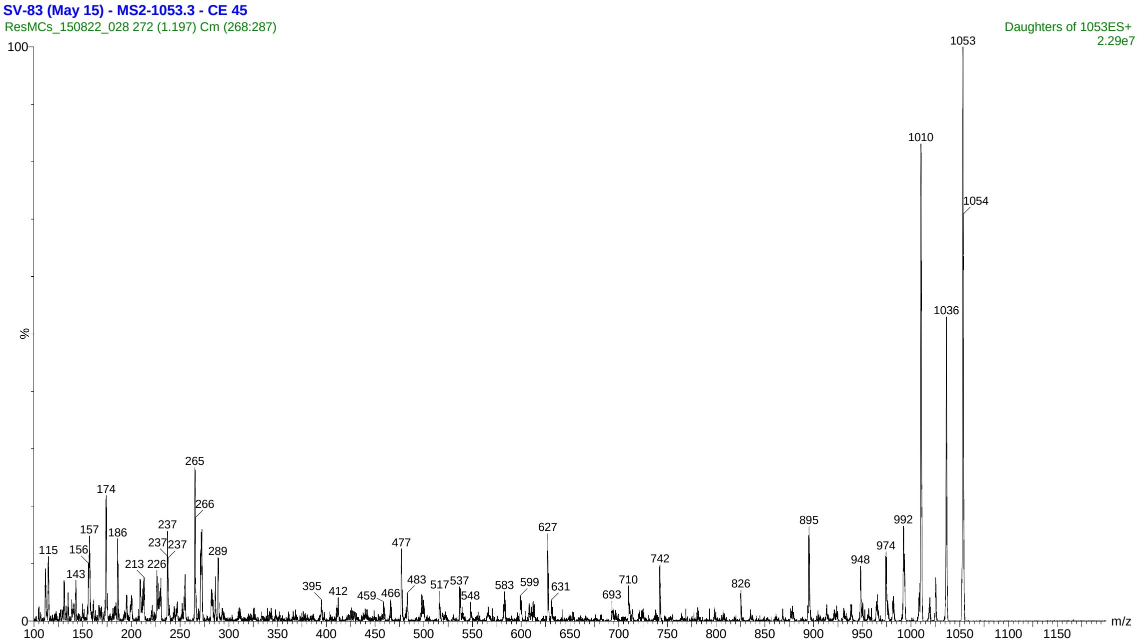

MS/MS spectrum of the *m/z* 1053.3 $[M+H]^{+}$ ion at 1.21 min from SV-83 (CE=45 V).

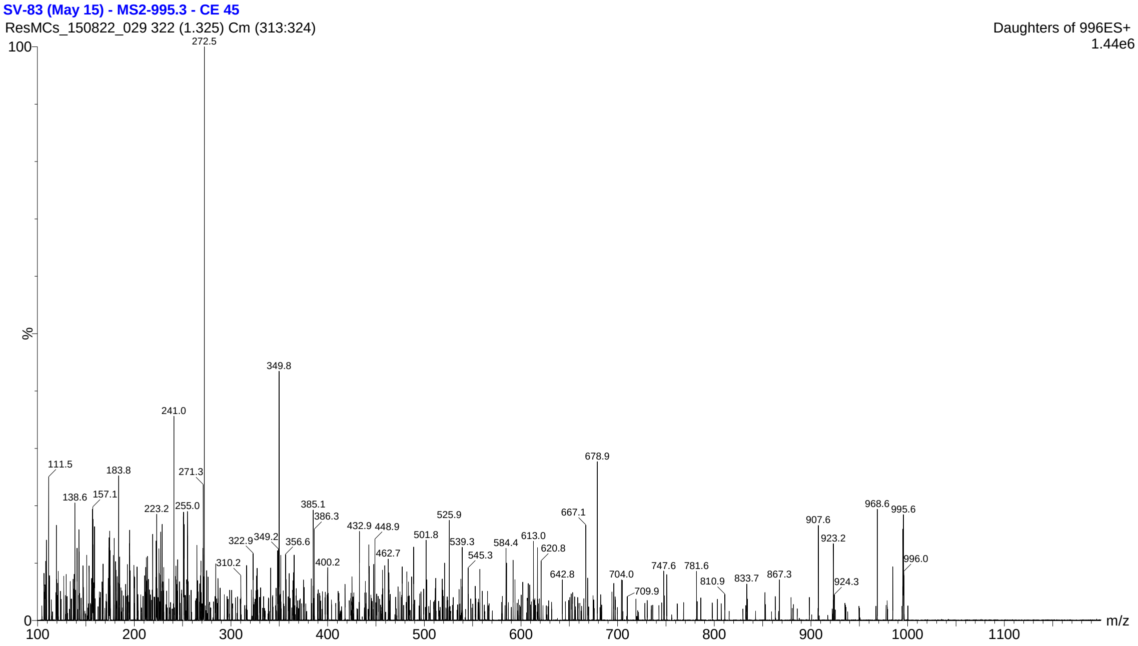

MS/MS spectrum of the *m/z* 995.5 ion at 1.33 min from SV-83 (CE=45 V).

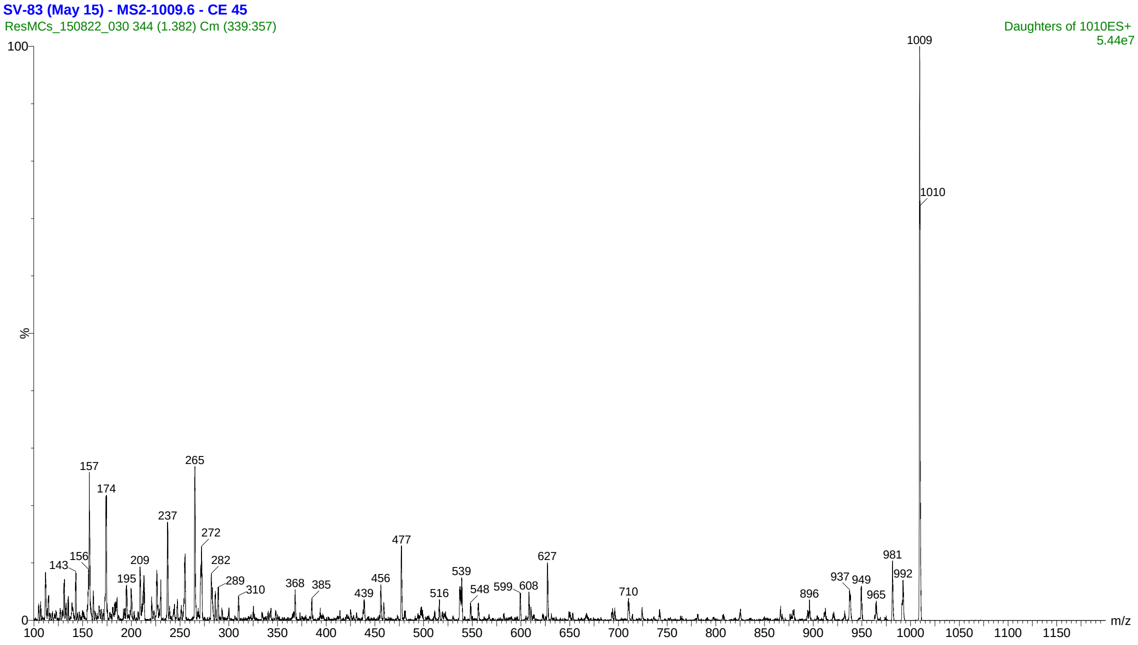

MS/MS spectrum of the *m/z* 1009.6 $[M+H]^+$ ion at 1.40 min from SV-83 (CE=45 V).

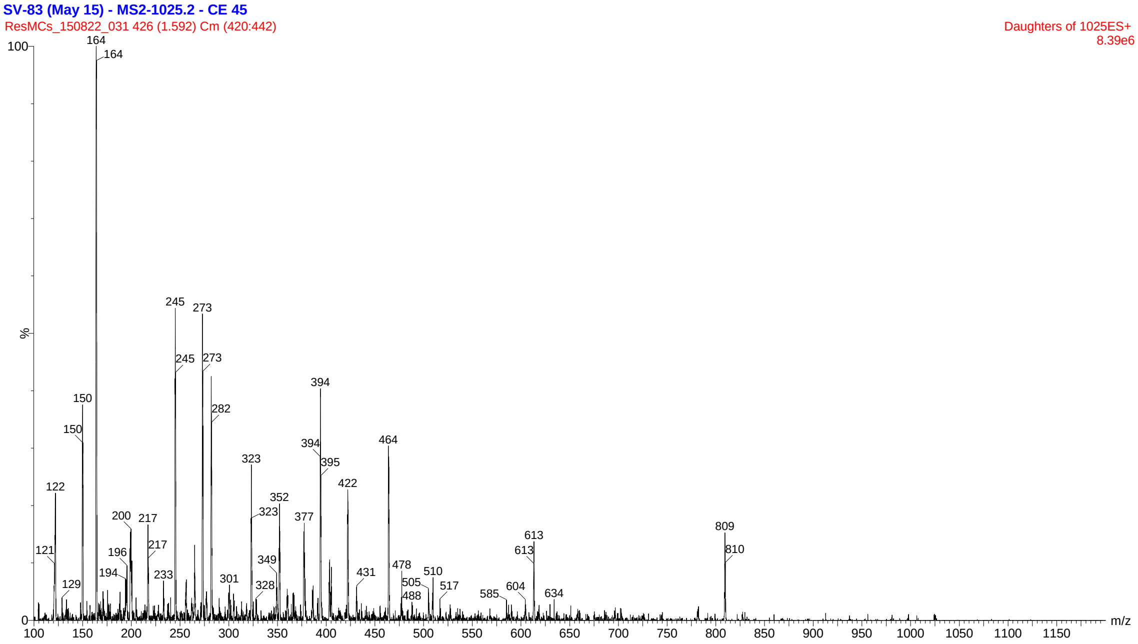

MS/MS spectrum of the *m/z* 1025.2 ion at 1.61 min from SV-83 (CE=45 V).

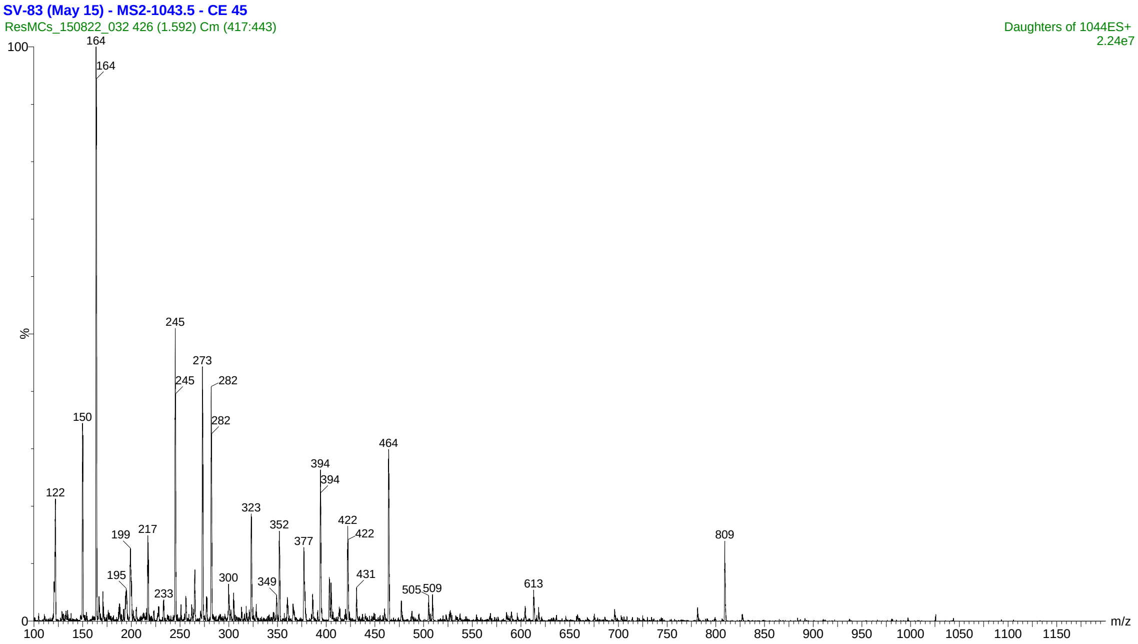

MS/MS spectrum of the *m/z* 1043.5 ion at 1.61 min from SV-83 (CE=45 V).

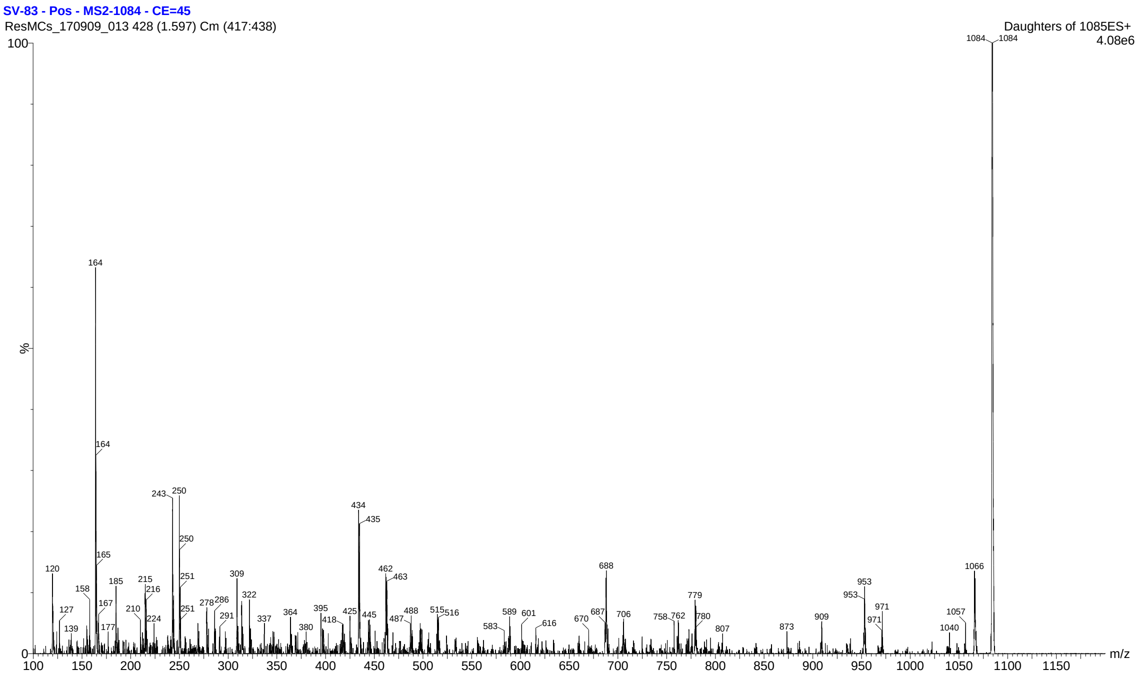

MS/MS spectrum of the *m*/*z* 1084.6 ion at 1.68 min from SV-83 (CE=45 V).
